# Supplementary material for: Structural basis of glycan276-dependent recognition by HIV-1 broadly neutralizing antibodies
Source: Cell Rep. Author manuscript; Available in PMC 2022 May 2. (PMC9058982; doi:10.1016/j.celrep.2021.109922)
Supplement: 1 [file NIHMS1798347-supplement-1.pdf]

Cell Reports, Volume *37*

Supplemental information

Structural basis of glycan276-dependent recognition by HIV-1 broadly neutralizing antibodies

Christopher A. Cottrell, Kartik Manne, Rui Kong, Shuishu Wang, Tongqing Zhou, Gwo-Yu Chuang, Robert J. Edwards, Rory Henderson, Katarzyna Janowska, Megan Kopp, Bob C. Lin, Mark K. Louder, Adam S. Olia, Reda Rawi, Chen-Hsiang Shen, Justin D. Taft, Jonathan L. Torres, Nelson R. Wu, Baoshan Zhang, Nicole A. Doria-Rose, Myron S. Cohen, Barton F. Haynes, Lawrence Shapiro, Andrew B. Ward, Priyamvada Acharya, John R. Mascola, and Peter D. Kwong

Cell Reports, Volume *37*

Supplemental information

Structural basis of glycan276-dependent recognition by HIV-1 broadly neutralizing antibodies

Christopher A. Cottrell, Kartik Manne, Rui Kong, Shuishu Wang, Tongqing Zhou, Gwo-Yu Chuang, Robert J. Edwards, Rory Henderson, Katarzyna Janowska, Megan Kopp, Bob C. Lin, Mark K. Louder, Adam S. Olia, Reda Rawi, Chen-Hsiang Shen, Justin D. Taft, Jonathan L. Torres, Nelson R. Wu, Baoshan Zhang, Nicole A. Doria-Rose, Myron S. Cohen, Barton F. Haynes, Lawrence Shapiro, Andrew B. Ward, Priyamvada Acharya, John R. Mascola, and Peter D. Kwong

Table S1. Heavy and light chain DNA sequences of CH540-VRC33 and CH314-VRC40 antibodies, related to Figure 1. For heavy chain, the sequence of VDJ region was shown. For light chain, the sequence of VJ region was shown.

Antibody	Heavy chain	Light chain
VRC33.01	CAGGTGCAGCTACAACAGTGGGGCGCAGGACTGTTGAAGCC<br>TTCGGAGACCCTGTCCCTCACGTGCGCTCTTTATGGTCGGT<br>CCCTCAATGGCAACTATTGGAGTTGGATCCGCCAGTCCCCA<br>GGGAAGGGGCTGGAGTGGATTGGGGAGATCAATCATAGTGG<br>AAGCACCTACTTCAACCCGTCCTTCAAGAGTCGCGTCGCCA<br>TGTCAGTGGACACGTCTAAGAGTCAATTCTCCCTGAAGCTG<br>AATTCTGTGACCGCCGCGGACACGGGTATTTATTTCTGTGC<br>GCGAGGCAAAAGATACAGCGCCAGCTACTCTAATTACTTTG<br>GCGTCTGGGGCCAGGGAACCCAGGTCACCGTCTCCTCAG	GACATCCAGATGACCCAGTCTCCTTCCACCCTGT<br>CTGCATCTGTAGGAGACAGAGTCGACATCACTTG<br>CCGGGCCAGTCAGAGTATTAGTCGCTGGTTGGCC<br>TGGTATCAGCAGAAGCCAGGAAAAGCCCCTAAAG<br>TCCTCATCTATGAGGCGTCTCTTTTAGCAAATGG<br>AGTCCCATCCAGGTTCAGCGGACACTTCAACGGC<br>AGAGAGTCTGCGACAGACTTCACACTCACCATCA<br>GCAGCCTGCAGCCTGACGATGTTGCAACTTATTA<br>CTGCCAACATTATATGGCTGATCCCCGTTTTGGC<br>CAGGGGACCAAGCTGGAGATCAAAC
VRC40.01	CAGGTGCAGTTGATACAGTCCGGGCCTCAGTTTAAGACGCC<br>TGGGGCCTCAGTGACGGTCTCGTGCAAGGCCTCAGGATACA<br>TCTTCACCGACTACCTCATACACTGGGTGCGGCTGGTCCCC<br>GGAAAGGGCCTTGAGTGGCTGGGCCGAATCAACACCAATGC<br>TGGTCTTATGTATCTTTCACATAAATTTGAGGGTCGCCTCA<br>TTCTGAGGAGAGTAGTTGACTGGAGGACACCGTCCCTGGGT<br>ACAGTCAACATGGAATTAAGGAACGTCAGATCTGACGACTC<br>GGCCATATATTTTTGTGGGCGGGTCGTCGACGGCTTTAACG<br>CGGCGGGCCCCCTTGAGTTTTGGGGCCAGGGAAGTCCAGTC<br>ATCGTTTCTTCA	CAAGTGGTGATGACGCAGTCTCCAGCCACCCTGT<br>CTCTGTCTCCGGGGGAGACGGCCGCCGTCTCCTG<br>CAGGGCCAGCCAATATGTTGACCGCTCTATATCT<br>TGGTATCAACTAAAAACTGGCCGGGCTCCCAGAC<br>TCCTCGTCTATGCTGCATCGTCCAGGTCCATTGG<br>TGTCCCAGACAGGTTCAGTGGCAGTGGGTCTGGG<br>AGAGACTTCACTCTCACCATCAGAGGCGTCCAGT<br>CTGACGACTTTGCACTTTATTACTGTCAACAAGA<br>TTACTACTGGCCGGTCACCTTCGGCCAAGGGACA<br>CGCCTGGACATGAAA
VRC40.02	CAGGTGCGATTGATGCAATCCGGGCCTCAACTTAAGACGCC<br>TGGGGCCTCAGTGACGGTCTCGTGCAAGGCCTCAGGATACA<br>TCTTCACCGACTACCTCATACACTGGGTGCGGCTAGTCCCC<br>GGAAAGGGCCTTGAGTGGCTGGGCCGAATCAACACCAATGG<br>TGGTCTTATGTACCTTTCATATAAATTTGAGGGTCGCCTCA<br>TTCTGAGGAGAGACGTTGACTGGAGGACACCGTCCCTGGGC<br>ACAGTCTACATGGAATTAAAGAACCTCAGATCTGACGACTC<br>GGCCATATATTTTTGTGGGCGAGTCGTCGACGGCTTTAACG<br>CGGCGGGCCCCCTTGAGTTTTGGGGCCAGGGAAGTCCAGTC<br>ATCGTTTCTTCA	CAAGTGGTGATGACGCAGTCTCCAGTCACCCTGT<br>CTGTGTCTCCGGGGGAGACGGCCGCCGTCTCCTG<br>CAGGGCCAGCCAATATGTTGACCGCTCTATATCT<br>TGGTATCAACTAAAAACTGGCCGGGCTCCCAGAC<br>TCCTCGTCTATGCTGCATCGTCCAGGTCCATTGG<br>TGTCCCAGACAGGTTCAGTGGCAGTGGGTCTGGG<br>AGAGACTTCACTCTCACCATCAGAGGCGTCCAGT<br>CTGACGACTTTGCAGTTTATTACTGTCAACAAGA<br>TTACTACTGGCCGGTCACCTTCGGCCAAGGGACA<br>CGCCTGGACATGAAA
VRC40.03	CAGGTGCAGTTGATACAGTCTGGGCCTCAACTTAAGACGCC<br>TGGGGCCTCAGTGACTGTCTCGTGCAAGGCCTCAGGATACG<br>TGTTCGCCGACTACCTCATACACTGGGTGCGACTGGTCCCC<br>GGAAAGGGCCTTGAGTGGCTGGGCCGAATCAACACCAATGC<br>TGGTCTTATGTACCTTTCACATAAATTTGAAGGTCGCCTCA<br>TTTTGAGGAGAGACCGTGACTGGAGGACACCGTCCCTGGGC<br>ACACTCTACATGGAATTAAGGAACCTAAAATCTGACGATTC<br>GGCCATATATTTTTGTGGGCGGGTCGTCGACGGCTTTAACG<br>CGGCGGGCCCCCTTGAGTTTTGGGGCCAGGGAAGTCCGGTC<br>ATCGTTTCTTCAG	CAAGTGTTGATGACGCAGTCTCCAGCCACCCTGT<br>CTGTGTCTCCGGGGGAGACGGCCGCCGTCTCCTG<br>CAGGGCCAGCCAGTATGTTGACCGCTCTATATCT<br>TGGTATCAGGTAAAAAGTGGCCGGGCTCCCAGAC<br>TCCTCGTCTATGCTGCATCGTCCAGGTCCATTGG<br>TGTCCCGGACAGGTTCAGTGGCAGTGGGTCTGGG<br>ACAGACTTCACTCTCACCATCAGAGGCGTCCAGT<br>CTGACGACTTTGCACTTTATTACTGTCAACAAGA<br>TTACGGCTGGCCGGTCACCTTCGGCCAAGGGACA<br>CGCCTGGACATGAAAG
VRC40.04	CAAGTGCGGTTGATGCAGTCTGGGACTGAATTTAAGACGCC<br>TGGGGCCTCAGTGAAGGTCTCGTGCAAGACCTCAGGATACA<br>TCTTCAGCGACTACTTAATACACTGGGTGCGACTAGTCCCC<br>GGAAAGGGCCTTGAGTGGCTGGGGCGGATCAACACTAACGC<br>TGGTCTTATGTACCTTTCACCGAGATTTGAGGGTCGCGTCA<br>TTCTGAGGAGAGAGAGTTCCTTCAGGACACCATCCCTGGGC<br>ACAGTCTACATGGAATTAAGGAACCTAAAATTTGACGACTC<br>GGCCGTCTACTTTTGTGGACGAGTCGTCGACGGATTTAACG<br>CGGCGGGCCCCCTTGAATTTTGGGGCCAGGGGAGCCTGGTC<br>ATCGTCTCCTCCG	GAAGTGGTGATGACGCAGTCTCCAGCCACCCTGT<br>CTGTGTCTCCGGGGGAAGAAGCCGCCCTCTCCTG<br>TGGGGCCAGCGACTATATTGACCGGTCTGTGTCC<br>TGGTATCAACTAAAACCTGGCCGGGCTCCCAGAC<br>TCCTCGTCTATGCTGCGTCCTCCAGGTCCATTGG<br>TATCCCAGACAGGTTCAGTGGCAGTGGGTCTGGG<br>ACAGCCTTCACTCTCACCATCAGAGGCGTCCAGT<br>CTGACGACTTTGCCCTCTATTACTGTCAACAAGA<br>CAAATACTGGCCGGTCACCTTCGGCCAAGGGACA<br>CGCCTGGACATGAAAC

Table S3. Cryo-EM data collection, refinement and validation statistics, related to Figures 2, 3 and 5.

	VRC40.01 and RM19R with BG505 SOSIPv5.2	VRC40.01 with BG505 DS-SOSIP	VRC33.01 with BG505 DS-SOSIP	179NC75 with Q23.17 RnS-SOSIP
Access codes	PDB: 7LG6<br>EMD-23312	PDB: 7LL1<br>EMD-23411	PDB: 7LL2<br>EMD-23412	PDB: 7LLK<br>EMD-23424
**Data collection and processing**				
Magnification	29,000	92,000	92,000	92,000
Voltage (kV)	300	300	300	300
Electron exposure ($e^-/Å^2$)	51.5	58.5	58.5	45
Defocus range (μm)	-0.8 to -3.6	-0.75 to -2.5	-0.75 to -2.5	-0.75 to -2.5
Pixel size (Å)	1.03	1.08	1.08	1.00
Symmetry imposed	C3	C3	C3	C3
Final particle number	32,186	327,299	220,262	41,476
Map resolution (Å)	3.28	3.73	3.73	4.8
FSC threshold	0.143	0.143	0.143	0.143
**Refinement**				
Map sharpening B factor ($Å^2$)	-81.8	-74.1	-61.9	-77.8
Model composition				
Non-hydrogen atoms	26565	25320	24852	20163
Protein residues	3153	3135	3060	2400
Ligands BMA	9	9	6	21
FUC	3	0	0	0
MAN	24	12	15	18
NAG	99	51	78	66
B factors ($Å^2$) (min/max/mean)				
Protein	0.0/321.3/44.5	1.0/255.1/13.8	0.5/204.4/4.4	0.0/113.4/9.4
Ligand	21.3/119.8/66.6	30.0/30.0/30.0	30.0/30.0/30.0	30.0/30.0/30.0
R.m.s. deviations				
Bond lengths (Å)	0.022	0.013	0.013	0.003
Bond angles (°)	1.75	1.90	1.79	0.713
Validation				
MolProbity score	1.00	1.25	1.07	2.13
Clash score	0.61	0.53	0.38	1.76
Rotamer outliers (%)	0.33	1.43	1.14	5.09
Ramachandran plot				
Favored (%)	95.75	93.02	93.43	85.51
Allowed (%)	3.57	6.62	6.11	11.54
Disallowed (%)	0.68	0.36	0.46	2.95
EMRinger score	3.99	2.56	2.70	1.15

Table S4. Crystallographic data and refinement statistics, related to Figures 2 and 3.

**Protein complex**	VRC33.01 Fab	VRC40.01 Fab
**PDB accession code**	7L77	7L79
**Data collection**		
Growth condition		
Space group	P 1 2_1 1	P2_1 2_1 2_1
Cell constants		
a, b, c (Å)	49.2, 70.8, 65.4	69.8, 136.0, 136.9
α, β, γ (°)	90.0, 99.8, 90.0	90, 90, 90
Unique reflections	62495 (5581)	27995 (2392)
Wavelength (Å)	1.0000	1.0000
Resolution (Å)	28.59 - 1.54 (1.60 - 1.54)	35.63 - 2.83 (2.93 - 2.83)
R_{sym}	7.9 (52.0)	20.6 (79.6)
R_{pim}	5.0 (36.0)	12.4 (50.8)
$CC_{1/2}$	0.966 (0.674)	0.970 (0.895)
I / σI	27.8 (2.5)	10.3 (1.5)
Completeness (%)	96.2 (86.0)	97.0 (84.7)
Redundancy	3.5 (2.8)	3.7 (3.3)
Wilson B-Factor (Å^2)	17	55
**Refinement**		
Resolution (Å)	29.21 - 1.54 (1.58 -1.54)	36.92 - 2.83 (2.93-2.83)
Reflections used in refinement	62487 (5579)	27954 (2388)
R_{work} / R_{free} (%)	15.2 (23.2) / 18.7 (25.4)	22.0 (32.7) / 28.0 (37.8)
No. atoms		
Protein	3380	6776
Ligand/ion	0	9
Water	516	100
B-factors (Å^2)		
Protein	24	50
Ligand/ion	-	62
Water	37.6	42
R.m.s. deviations		
Bond lengths (Å)	0.010	0.007
Bond angles (°)	1.19	0.90
Ramachandran		
Favored regions (%)	97.94	92.37
Allowed regions (%)	2.06	6.95
Disallowed regions (%)	0.00	0.68

Values in parentheses are for the highest-resolution shells.

$R_{sym} = \Sigma|I - \langle I\rangle|/\Sigma\langle I\rangle$, where I is the observed intensity, and $\langle I\rangle$ is the average intensity of multiple observations of symmetry-related reflections.

$R = \Sigma_{hkl}||F_{obs}| - |F_{calc}||/\Sigma_{hkl}|F_{obs}|$.

R_{free} is calculated from 5% of the reflections excluded from refinement.

A

Serum	Date	VRC01-like	b12-like	CD4-like	HJ16-like	8ANC195-like	PG9-like	PGT128-like	2G12-like	2F5-like	10E8-like	Target function
CH314	5/12/08	0.00	0.00	0.00	0.46	0.31	0.00	0.00	0.23	0.00	0.00	24.8
CH540	1/24/08	0.23	0.21	0.00	0.36	0.20	0.00	0.00	0.00	0.00	0.00	21.1

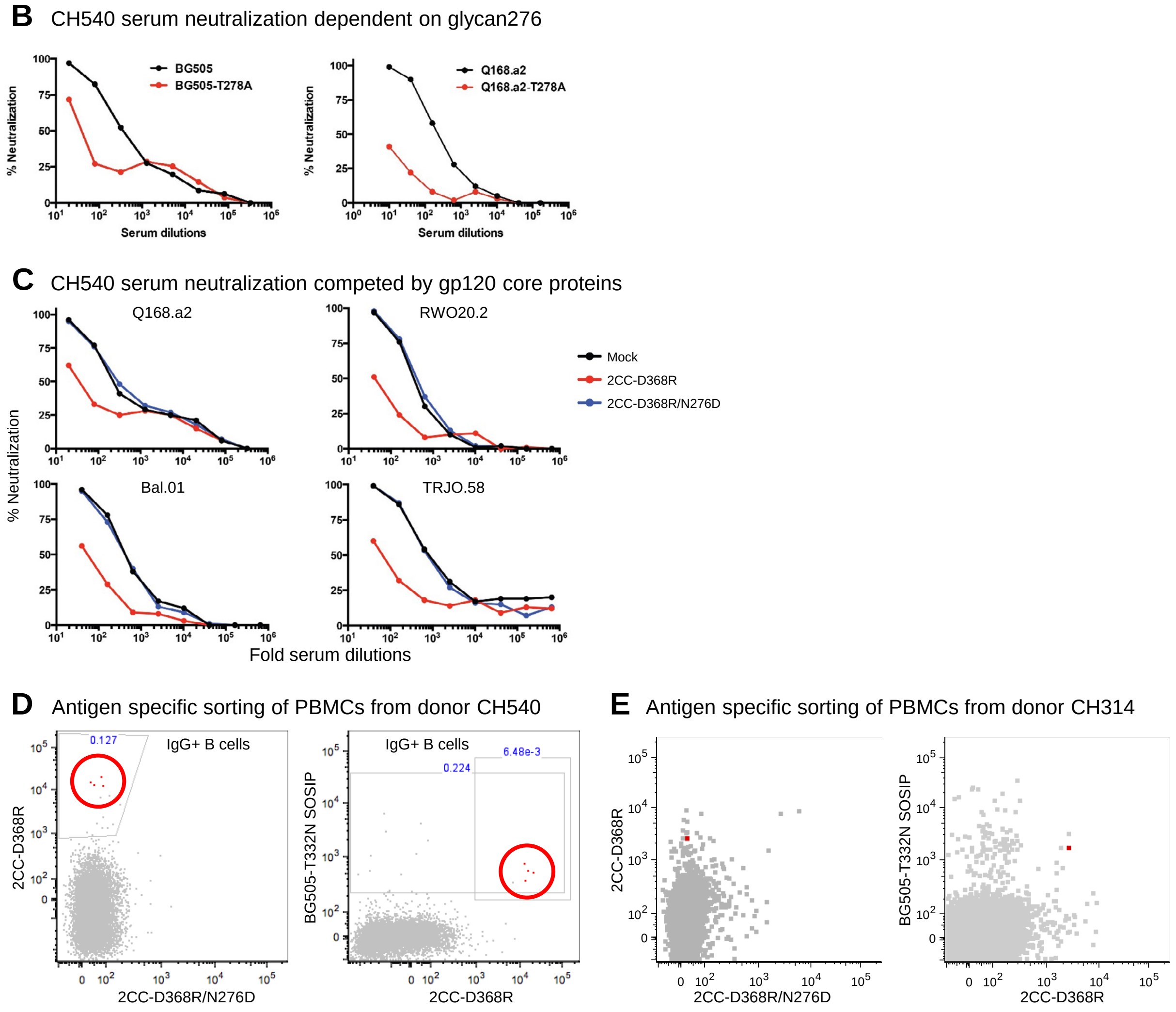

Figure S1. Serum neutralization analyses indicate Donor CH540 and CH314 sera to contain CD4bs-targeting antibodies that are glycan276 dependent, related to Figure 1.

(**A**) Neutralization fingerprint analysis of serum samples from donors CH540 indicated presence of neutralizing antibodies similar to VRC01 and b12, as well as glycan276-dependent antibodies HJ16 and 8ANC195. (**B**) CH540 serum neutralization of HIV-1 strains is dependent on the presence of glycan276. (**C**) CH540 serum neutralization of strains Q168.a2, RWO20.2, Bal.01, and TRJO.58 was competed by gp120 core protein 2CC-D368R but not by the glycan276-knockout mutant 2CC-D368R/N276D. (**D**) Antigen-specific B cell sorting identified four PBMCs from donor CH540 that bound to gp120 core only when glycan276 was present. $CD3^{-}CD8^{-}CD14^{-}CD19^{+}IgG^{+}$ memory B cells were stained with probes 2CC-D368R, 2CC-D368R/N276D, and gp140 trimer BG505 T332N.SOSIP. (**E**) Antigen-specific B cell sorting identified one PBMC from donor CH314 that bound to gp120 core only when glycan276 was present.

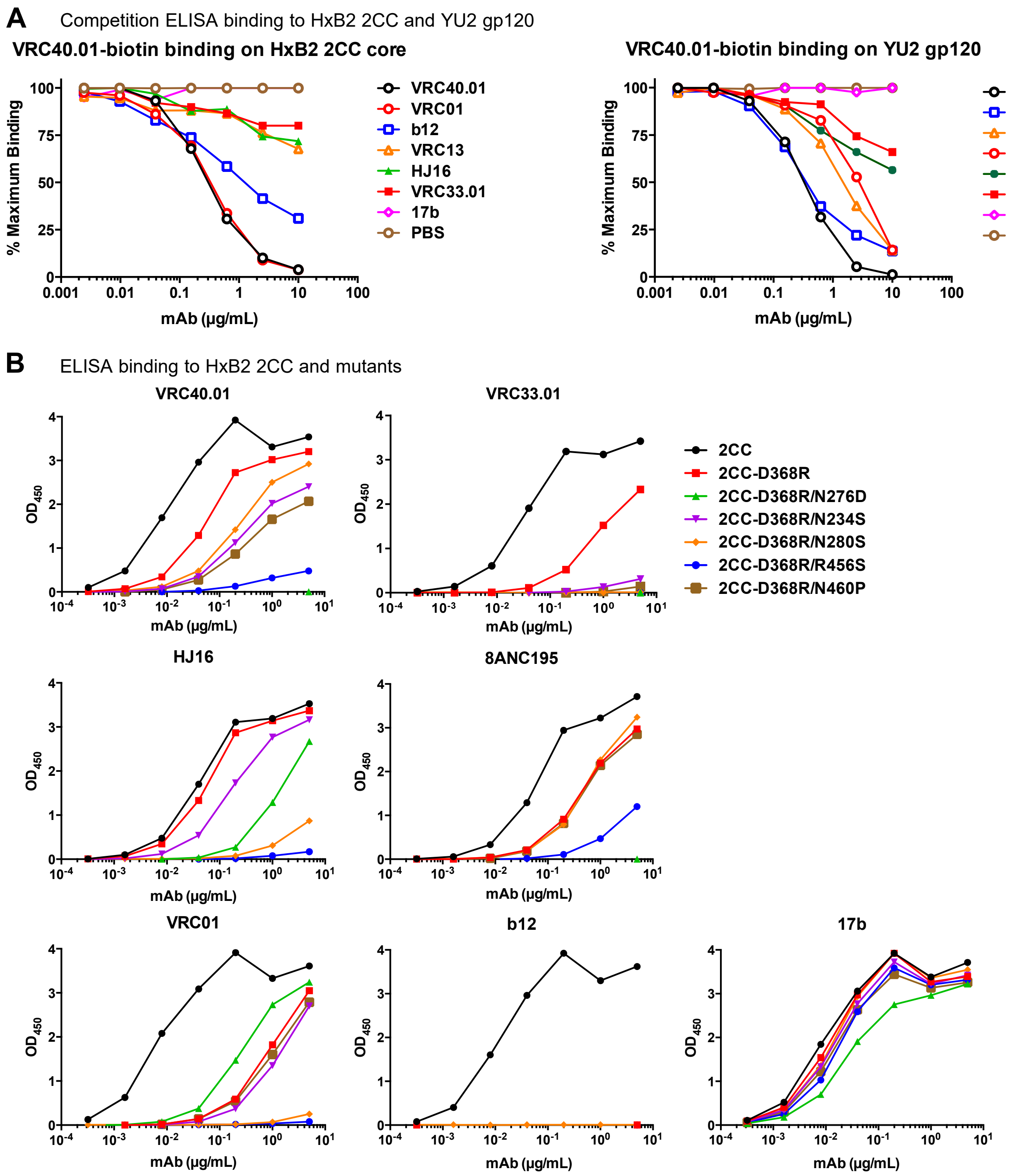

Figure S2. Binding analyses indicate CH540-VRC40.01 and CH314-VRC33.01 to target the CD4bs and glycan276, related to Figure 1.

(**A**) Competition ELISA for binding of biotinylated CH540-VRC40.01 to HxB2 2CC core (left) or YU2 gp120 (right) by CH314-VRC33.01 and various CD4bs antibodies. Percent maximum binding is plotted against competing antibody concentration. (**B**) ELISA binding of VRC40.01and VRC33.01 to HxB2 gp120 2CC core and its various mutants. Parallel assays were performed for glycan276-dependent antibodies HJ16 and 8ANC195, CD4bs Abs VRC01 and b12, and CD4i antibody 17b for comparison.

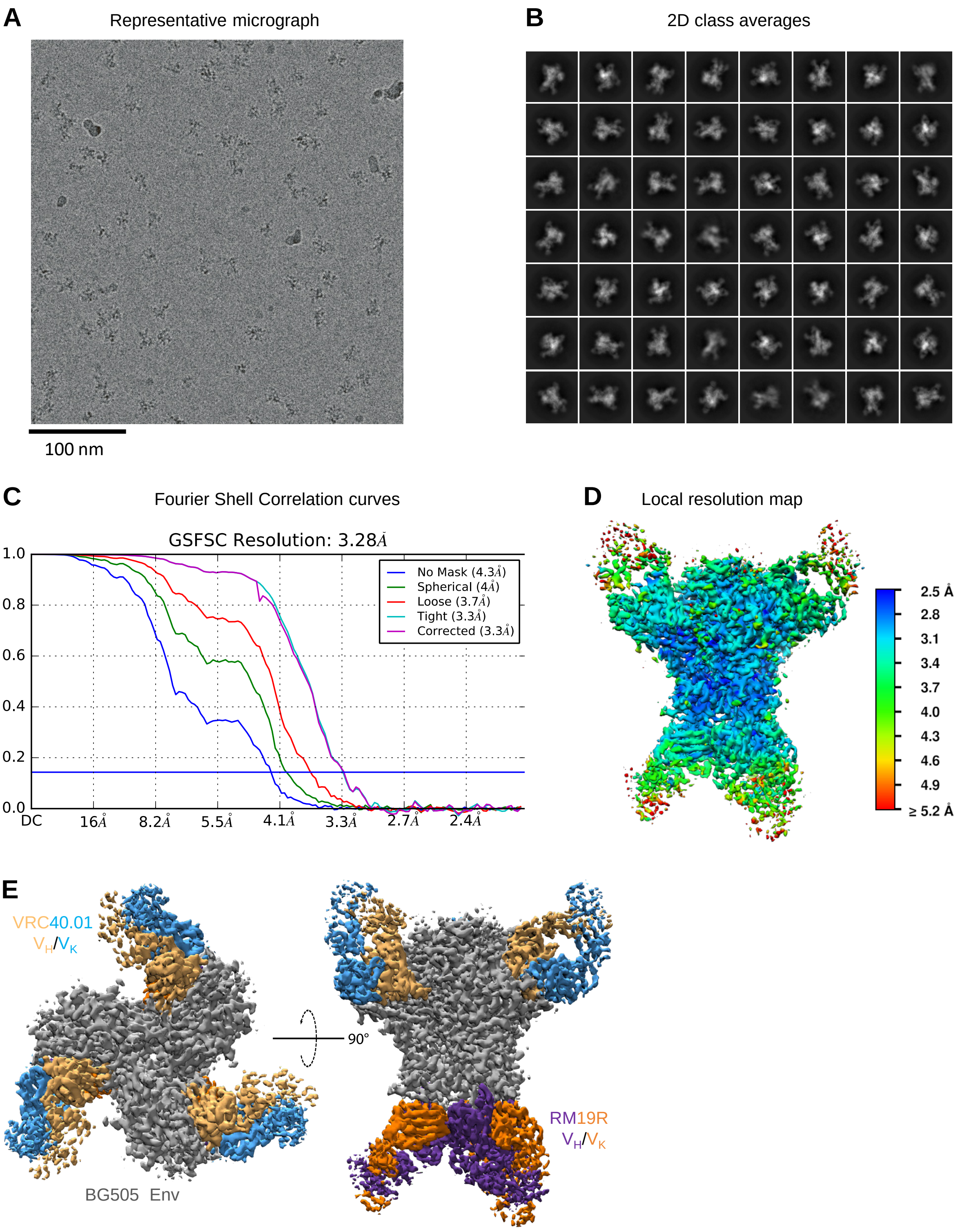

Figure S3. Cryo-EM data and structure validation for VRC40.01 and RM19R Fabs in complex with BG505 SOSIP.v5.2, related to Figure 2.
(**A**) Representative aligned and dose weighted micrograph collected on a Gatan K2 Summit. (**B**) Representative 2D class averages calculated from selected particles. (**C**) Gold-standard FSC curves of the final 3D reconstruction. (**D**) Refined 3D density map colored by local resolution. (**E**) 3.3-Å resolution cryo-EM reconstruction of VRC40.01 bound to BG505 SOSIP.v5.2. A base-binding monoclonal Fab RM19R was added to the complex to improve particle orientations.

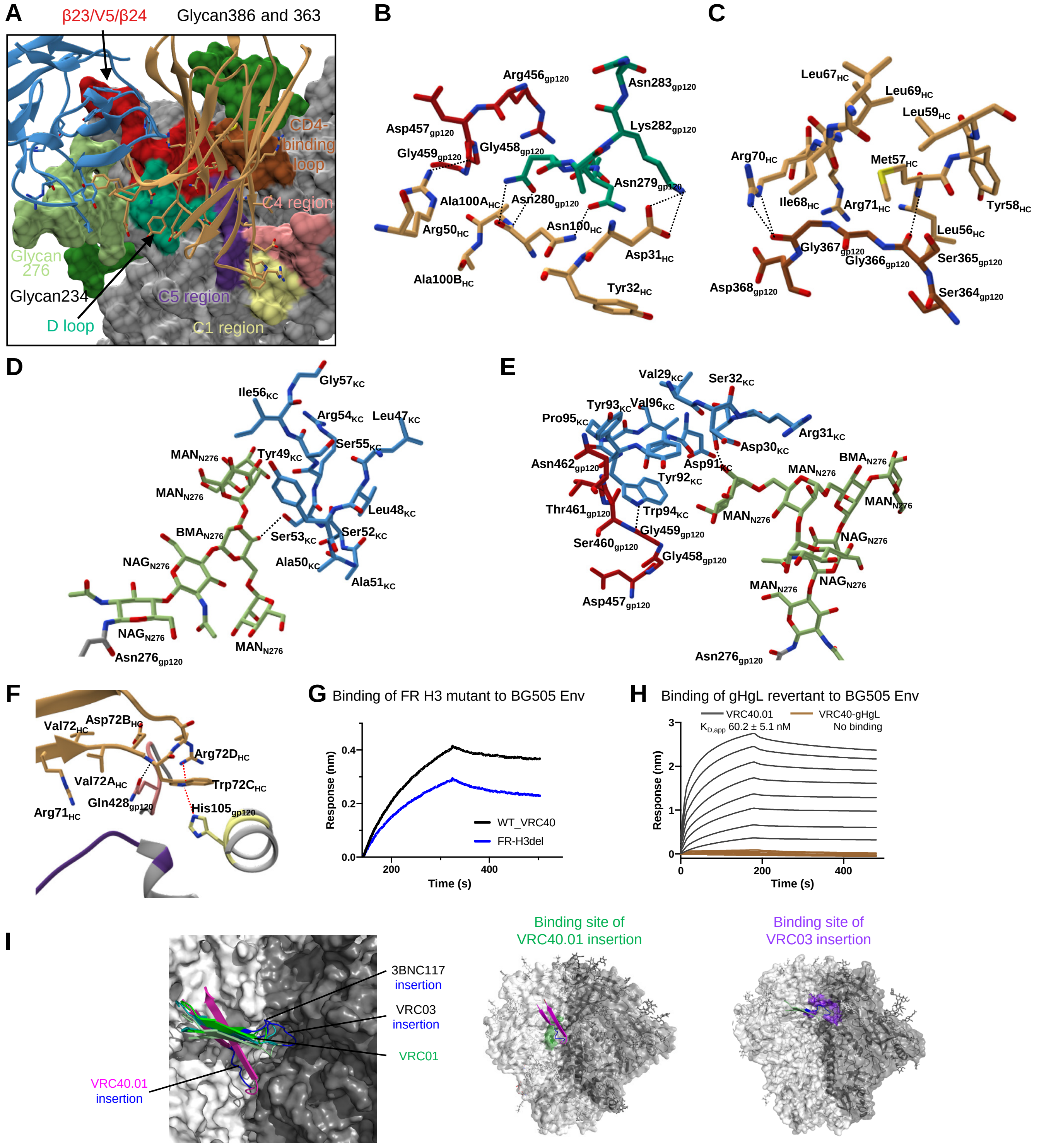

Figure S4. Structural and biochemical analyses of VRC40.01 binding to BG505 Env, related to Figure 2.
(**A**) Env gp120 regions at or near VRC40.01 epitope: glycan276 (light green), D loop (turquoise), $\beta 23/V5/\beta 24$ (red), CD4-binding loop (brown), C1 region (yellow), C4 region (pink), and C5 (purple). Glycans flanking the VRC40.01 epitope are shown in dark green. (**B**) Molecular interactions between VRC40.01 and the D loop and $\beta 23/V5/\beta 24$ regions. (**C**) Molecular interactions between VRC40.01 and the CD4-binding loop. (**D-E**) Interactions of the light chain of VRC40.01 with glycan276 and the $\beta 23/V5/\beta 24$ region of BG505. (**F**) VRC40.01 FR-H3 insertion makes contact with the C1, C4, C5 regions of BG505. Hydrogen bonds are shown as black dotted lines and π-π interactions are depicted as red dotted lines in (B) through (F). (**G**) BLI assays of BG505 SOSIP.664 binding to either the wildtype VRC40.01 Fab or a mutant of VRC40.01 Fab with the 5-residue FR-H3 insertion removed. (**H**) BLI assays of BG505 Env binding to either the wildtype or gHgL-reverted VRC40.01. BG505 Env trimer was captured on Streptavidin sensor tips and dipped into the antibody solutions at 0.05-6.5 μM. (**I**) Comparison of binding mode of FR-H3 insertions in VRC40.01 and VRC01-class antibodies. Contact surface of FR-H3 insertions are colored green and purple blue for VRC40.01 and VRC03, respectively.

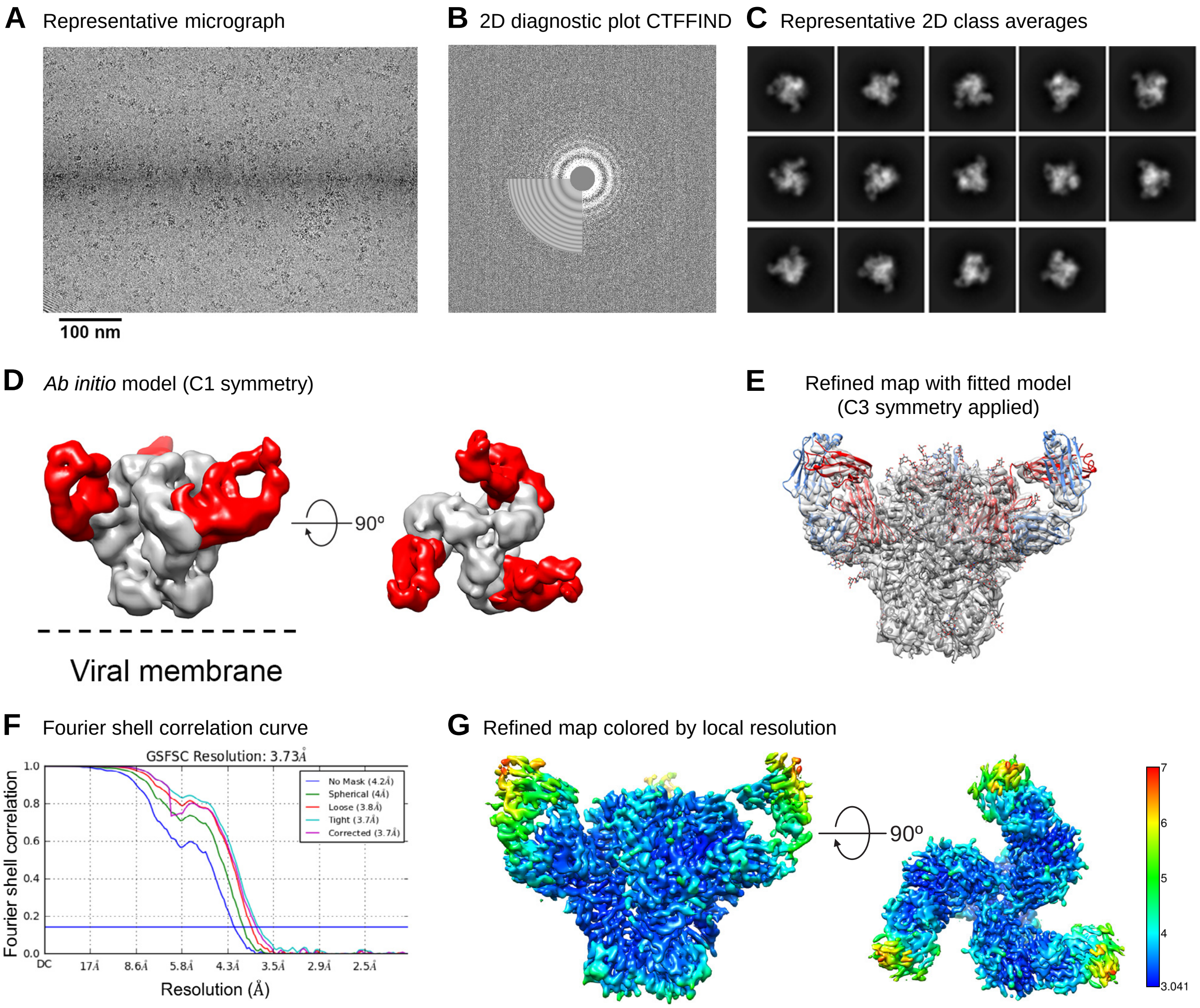

Figure S5. Cryo-EM data and structure validation for VRC40.01 Fab in complex with BG505 DS-SOSIP Env trimer, related to Figure 2.

(**A**) Representative micrograph of VRC40.01 Fab in complex with BG505 DS-SOSIP Env trimer. (**B**) 2D diagnostic plot of the micrographs using CTFFIND4. (**C**) Representative 2D class averages calculated from selected particles. (**D**) *Ab initio* model showing BG505 Env (grey) and VRC40.01 Fab (red). (**E**) C3 symmetry applied refined map of *ab initio* generated model refined against cleaned-up particles. (**F**) FSC curves of the 3D reconstructions with horizontal blue line indicating $FSC_{0.143}$. (**G**) Refined 3D density map colored by local resolution with side and top views.

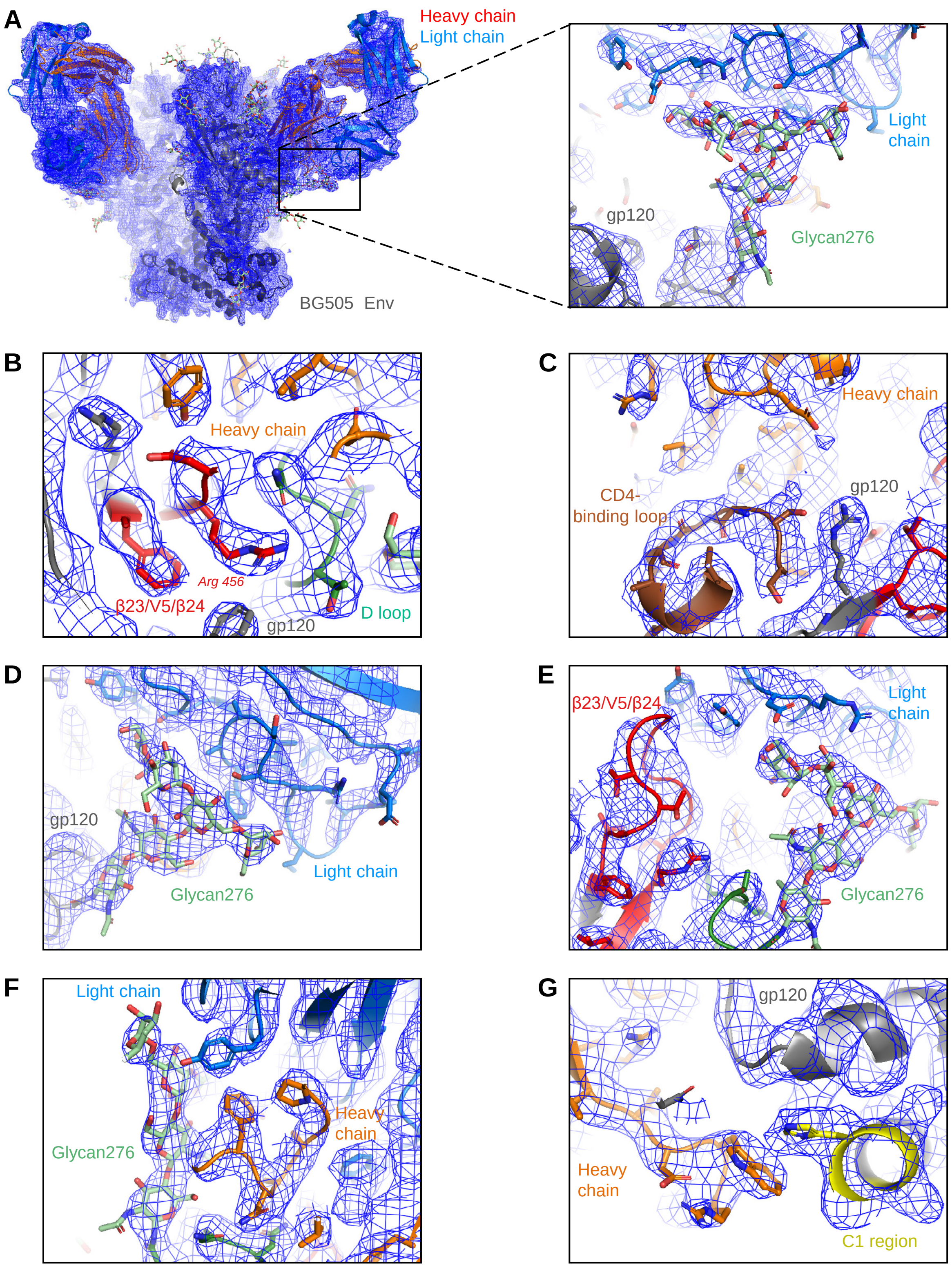

Figure S6. Cryo-EM density of VRC40.01 bound to BG505 DS-SOSIP Env trimer, related to Figure 2.
(**A**) Side view of VRC40.01 bound to BG505 DS-SOSIP structure fitted in cryo-EM density (blue mesh). Right panel shows a zoomed-in view of glycan276 fitted into the density. (**B-G**) Zoomed-in views of interactions between key regions of gp120 at or near the VRC40.01 epitope. Blue mesh indicates experimental cryo-EM density.

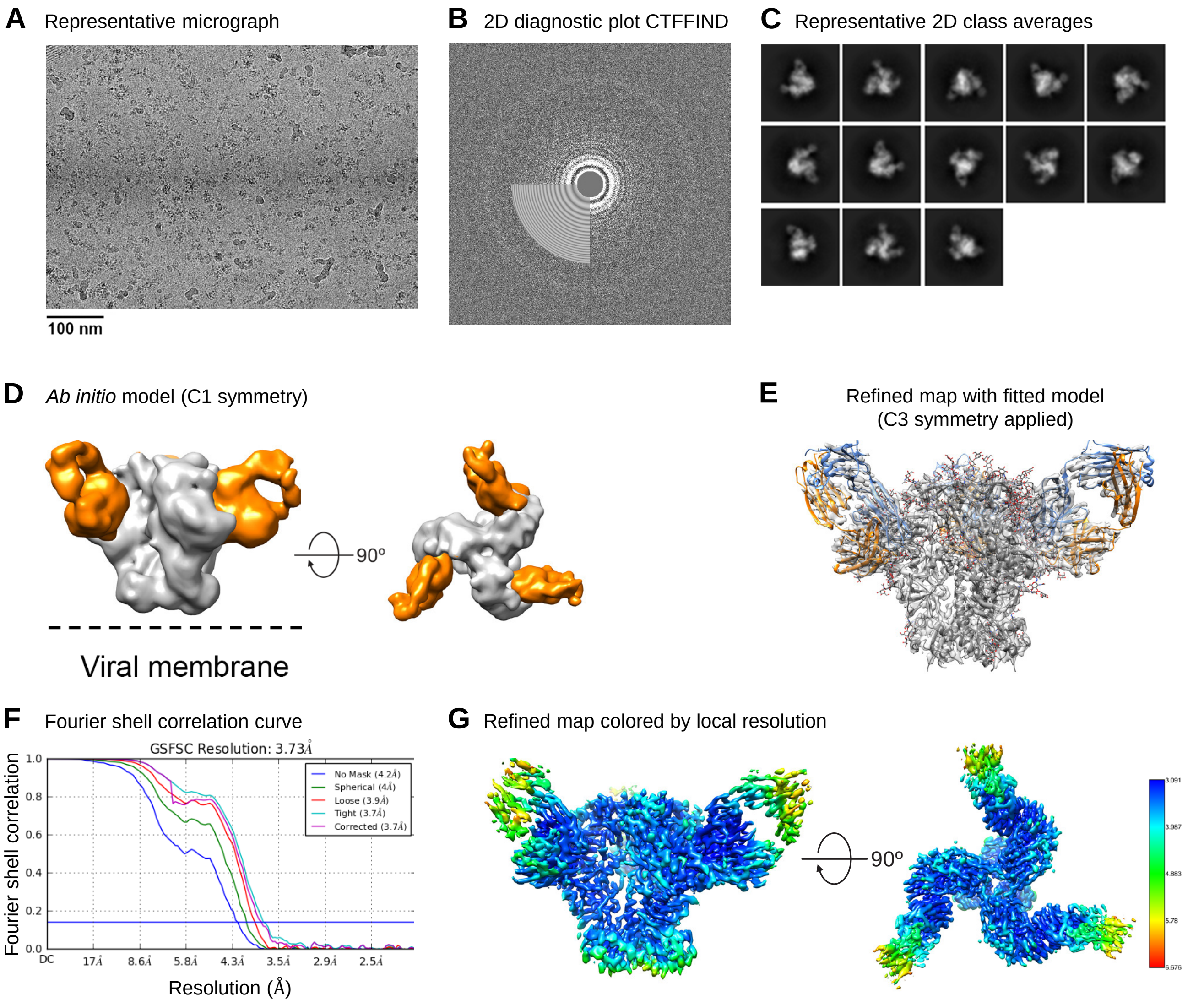

Figure S7. Cryo-EM data and structure validation for VRC33.01 Fab in complex with BG505 DS-SOSIP Env trimer, related to Figure 3.

(**A**) Representative micrograph of VRC33.01 Fab in complex with BG505 DS-SOSIP Env trimer. (**B**) 2D diagnostic plot of the micrographs using CTFFIND4. (**C**) Representative 2D class averages calculated from selected particles. (**D**) *Ab initio* model showing BG505 Env (grey) and VRC33.01 Fab (orange). (**E**) C3 symmetry applied refined map of *ab initio* generated model refined against cleaned-up particles. (**F**) FSC curves of the 3D reconstructions with horizontal blue line indicating $FSC_{0.143}$. (**G**) Refined 3D density map colored by local resolution with side and top views.

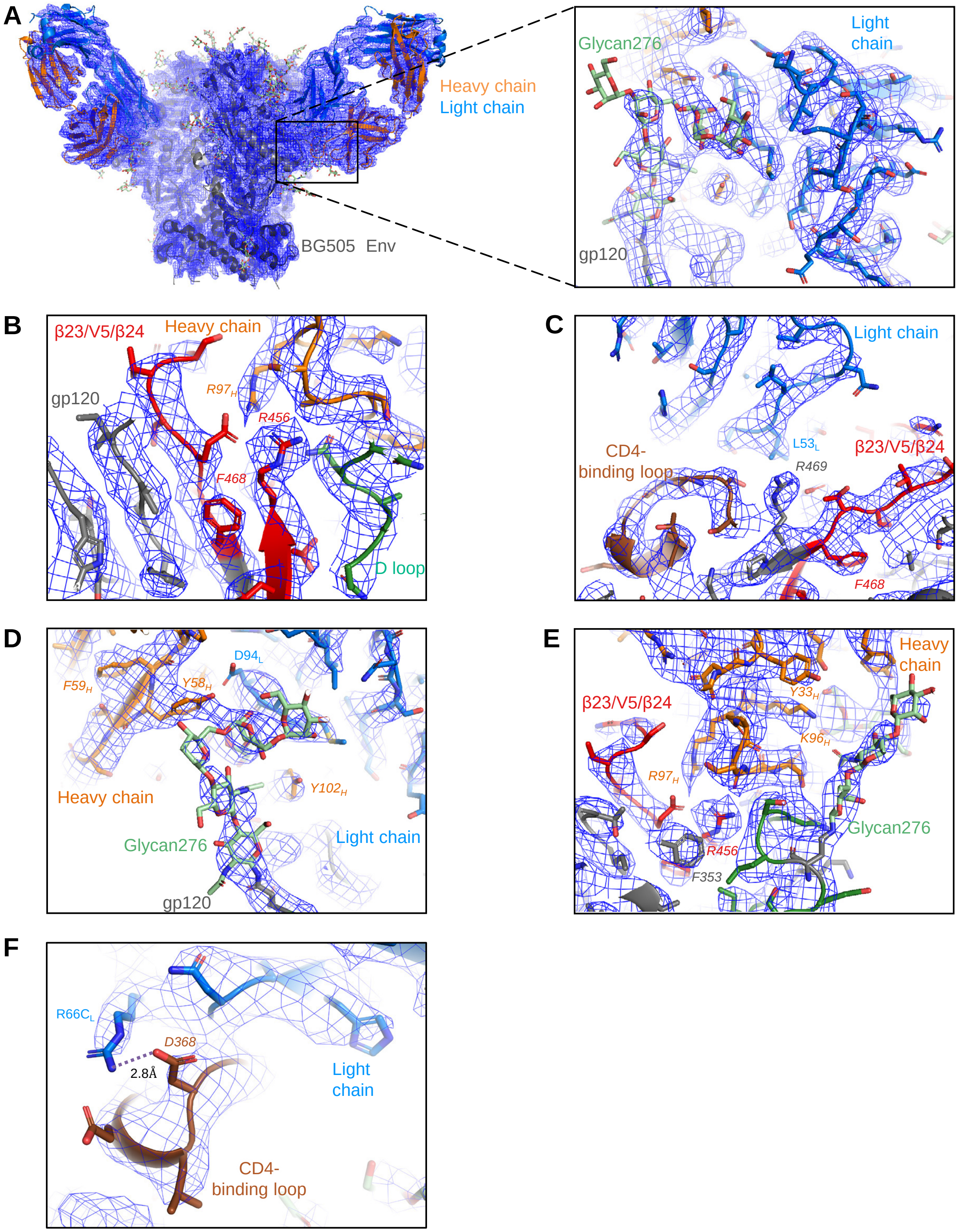

Figure S8. Cryo-EM density of VRC33.01 bound to BG505 DS-SOSIP Env trimer, related to Figure 3.
(**A**) Side view of VRC33.01 bound to BG505 DS-SOSIP structure fitted in cryo-EM density (blue mesh). Right panel shows a zoomed-in view of glycan276 fitting into the density. (**B-E**) Zoomed-in views of interactions between key regions of gp120 at or near the VRC33.01 epitope. Blue mesh indicates experimental cryo-EM density. (**F**) Asp368 of BG505 DS-SOSIP makes a salt bridge with Arg66C of VRC33.01 light chain.

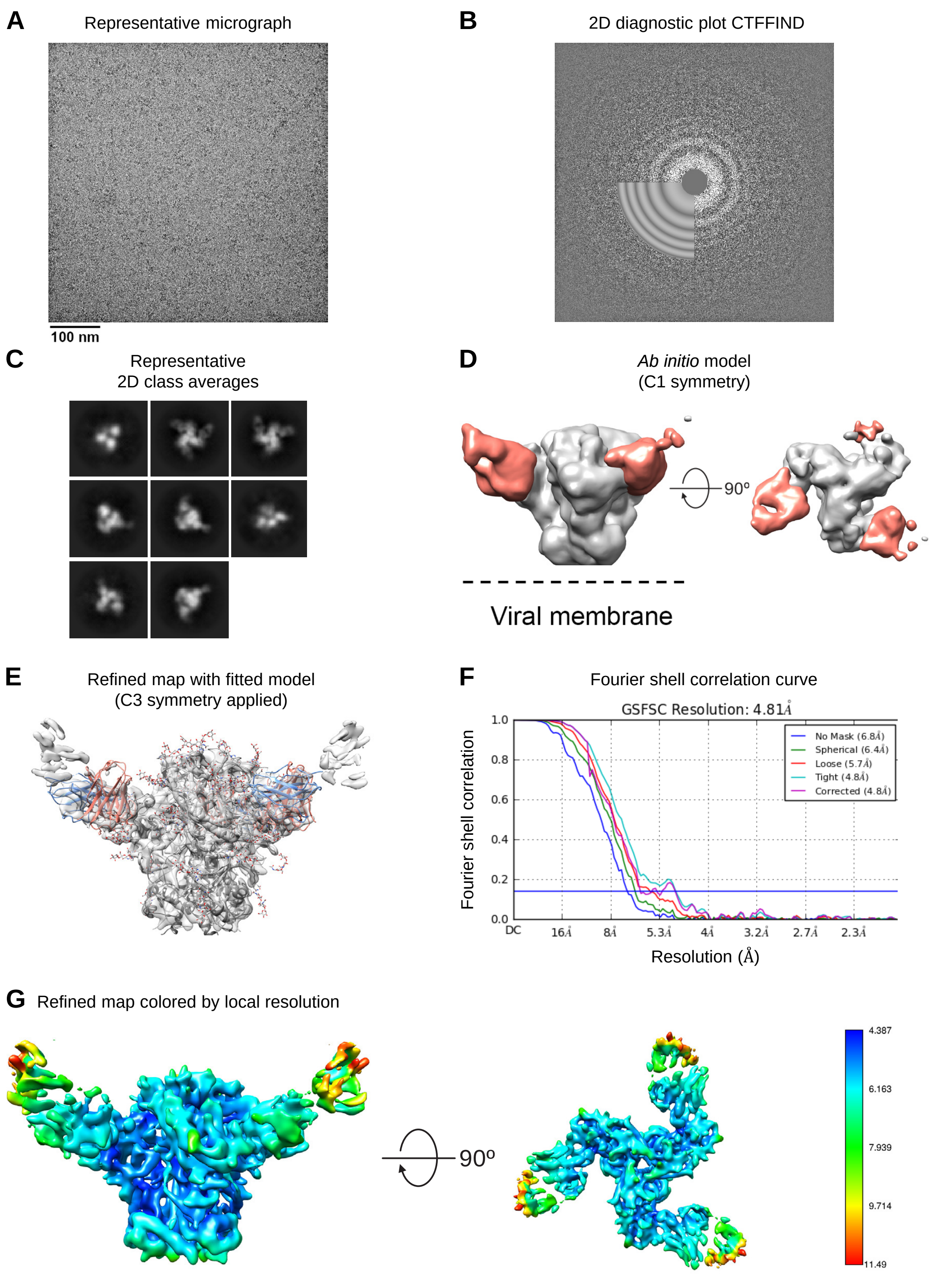

Figure S9. Cryo-EM data and structure validation for 179NC75 Fab in complex with Q23.17_DS-SOSIP_RnS_V1V2-V3-fixed Env trimer, related to Figure 5.

(**A**) Representative micrograph of 179NC75 Fab in complex with Q23.17_DS-SOSIP_RnS_V1V2-V3-fixed Env trimer. (**B**) 2D diagnostic plot of the micrographs using CTFFIND4. (**C**) Representative 2D class averages calculated from selected particles. (**D**) *Ab initio* model showing Q23.17_DS-SOSIP_RnS_V1V2-V3-fixed Env (grey) and 179NC751 Fab (salmon). (**E**) C3 symmetry applied refined map of *ab initio* generated model refined against cleaned-up particles. (**F**) FSC curves of the 3D reconstructions with horizontal blue line indicating $FSC_{0.143}$. (**G**) Refined 3D density map colored by local resolution with side and top views.

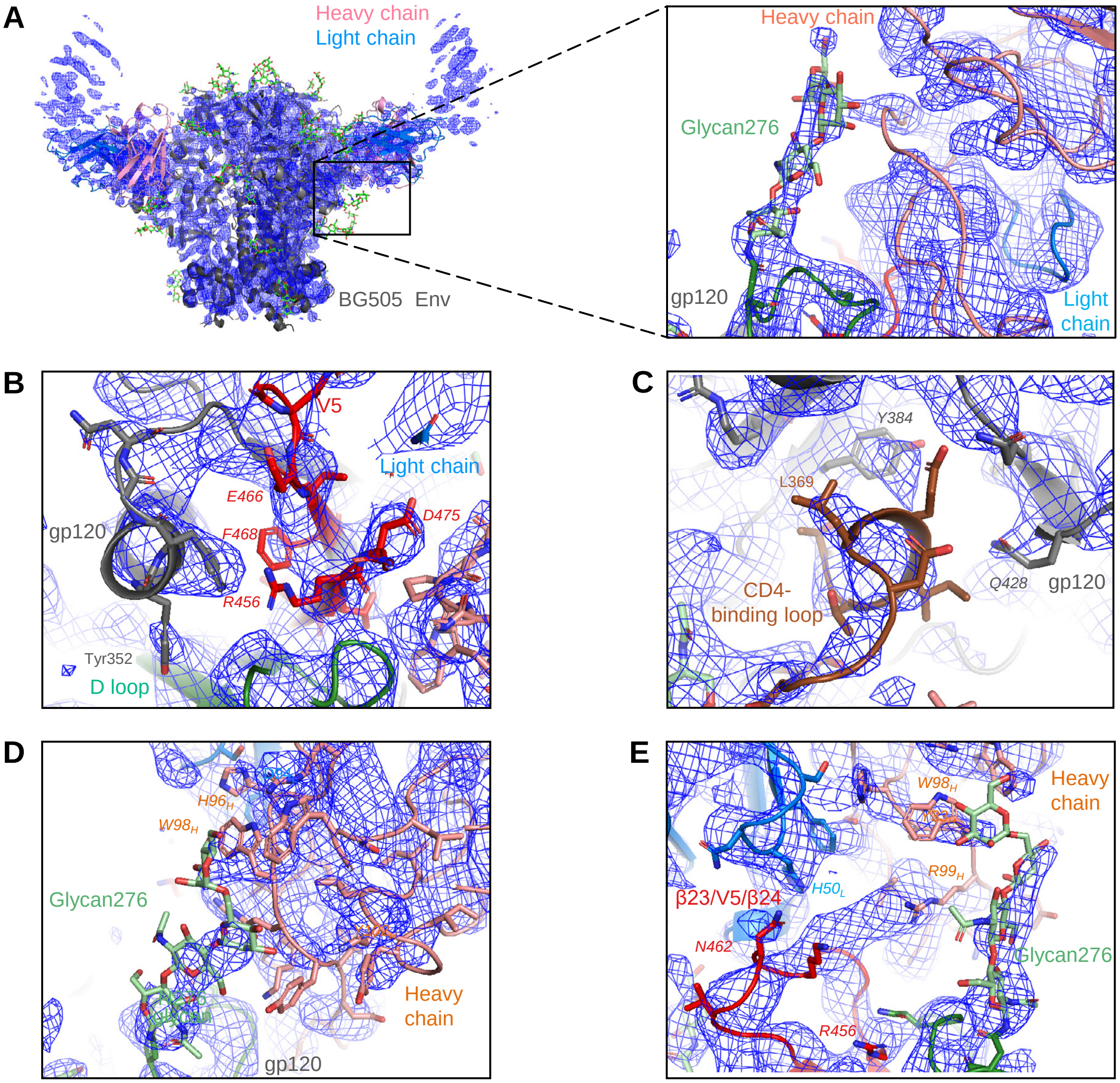

Figure S10. Cryo-EM density of 179NC75 Fab in complex with Q23.17_DS-SOSIP_RnS_V1V2-V3-fixed Env trimer, related to Figure 5.

(**A**) Side view of 179NC75 bound to Q23.17_DS-SOSIP_RnS_V1V2-V3-fixed structure fitted in cryo-EM density (blue mesh). Right panel shows a zoomed-in view of glycan276 fit into the density. (**B-E**) Zoomed-in views of interactions between key regions of Env gp120 regions at or near 179NC75 epitope. Blue mesh indicates experimental cryo-EM density.

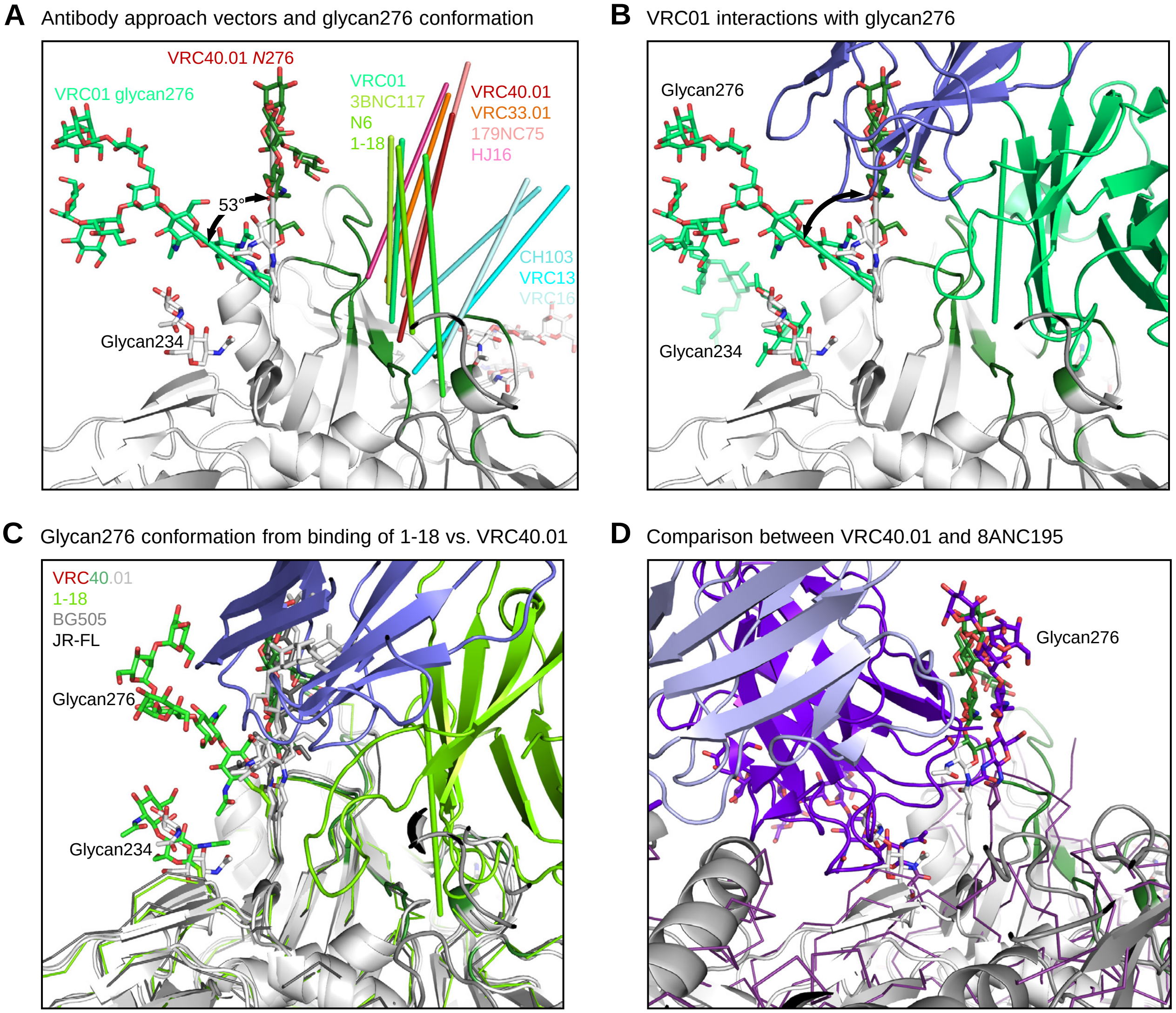

Figure S11. Antibody approach angles and glycan276 conformations, related to Figure 6.
(**A**) The angles and positions that antibodies approach Env trimer affect their interaction with glycan276. VRC01 binding tilts toward glycan276 relative to VRC40.01, and VRC01 binding pushes glycan276 53º away from CD4bs. BG505 Env is shown in light gray with VRC40.01 epitope residues in forest green. Glycan276 of the VRC01-Env complex is shown in lime green. Antibody approach vectors are shown as in Figure 6B. (**B**) VRC01 light chain interacts strongly with glycan276, which tilts away from the CD4bs to avoid clashes. (**C**) Effect on glycan276 conformation by binding of 1-18 in comparison with binding of VRC40.01 and glycan276 conformation free of glycan276-interacting antibodies. 1-18 (PDB: 6UDJ) is shown in yellow green with light chain in light blue. Only glycans at N276 and N234 are shown for other structures for clarity. Env structure without glycan276-binding antibodies (PDB: 5FYL, 5FUU) are colored as labeled. Env trimer in the VRC40.01 complex is shown as in (A). Binding of 1-18 pushes glycan276 away by 35º from that in VRC40.01, which maintains the orientation of glycan276 free of interacting antibody. (**D**) Glycan276 maintains the same conformation in 8ANC195 as in VRC40.01, despite their different binding locations on Env and binding glycan276 on opposite sides. 1-18 has strong interactions with glycan234.
