# Supplementary material for: Structural basis of glycan276-dependent recognition by HIV-1 broadly neutralizing antibodies
Source: Cell Rep. Author manuscript; Available in PMC 2022 May 2. (PMC9058982; doi:10.1016/j.celrep.2021.109922)
Supplement: 3 [file NIHMS1798347-supplement-3.pdf]

Cell Reports

Article

Structural basis of glycan276-dependent recognition by HIV-1 broadly neutralizing antibodies

Graphical abstract

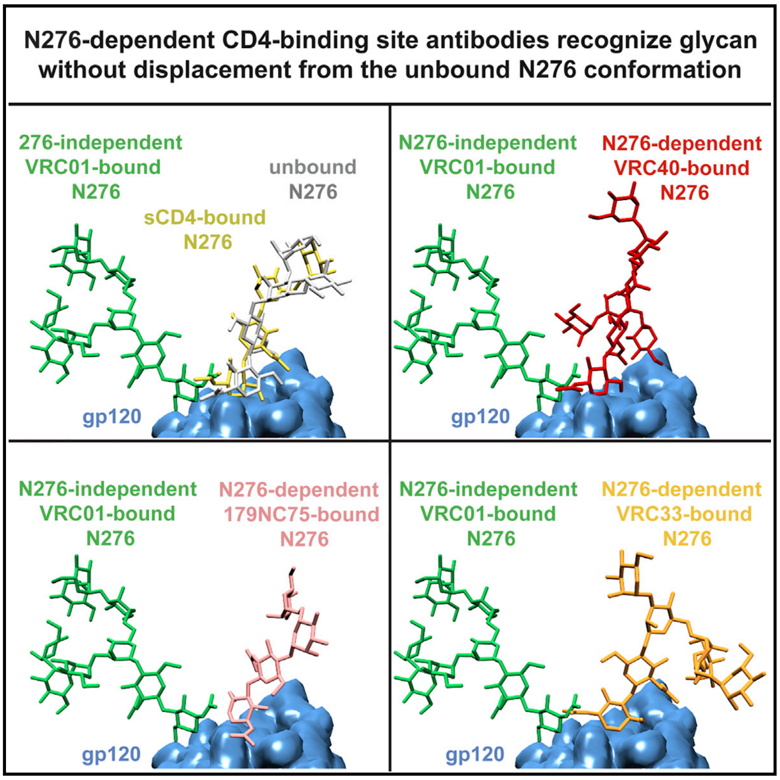

Highlights

- Glycan276-dependent antibodies with up to 81% neutralization breadth are isolated
- Structure for three glycan276-dependent antibodies with envelope trimer is determined
- Glycan276-dependent antibodies bind glycan276 with diverse modes of recognition
- Glycan276-dependent antibodies maintain ligand-free orientation of glycan276

Authors

Christopher A. Cottrell, Kartik Manne, Rui Kong, ..., Priyamvada Acharya, John R. Mascola, Peter D. Kwong

Correspondence

jmascola@nih.gov (J.R.M.), pdkwong@nih.gov (P.D.K.)

In brief

CD4 binding site-directed antibodies can broadly neutralize circulating HIV strains but often need to overcome glycan shielding, especially from glycan276. Cottrell et al. isolate two lineages of antibodies that depend on glycan276 for neutralization and elucidate the mechanistic difference between glycan276-dependent and glycan276-independent antibodies, with implications for HIV vaccine design.

Cottrell et al., 2021, Cell Reports *37*, 109922
November 2, 2021
https://doi.org/10.1016/j.celrep.2021.109922

CellPress

Cell Reports

CellPress
OPEN ACCESS

Article

Structural basis of glycan276-dependent recognition by HIV-1 broadly neutralizing antibodies

Christopher A. Cottrell,[1,7] Kartik Manne,[2,3,7] Rui Kong,[4,7] Shuishu Wang,[4,7] Tongqing Zhou,[4,7] Gwo-Yu Chuang,[4] Robert J. Edwards,[2,3] Rory Henderson,[2,3] Katarzyna Janowska,[2,3] Megan Kopp,[2,3] Bob C. Lin,[4] Mark K. Louder,[4] Adam S. Olia,[4] Reda Rawi,[4] Chen-Hsiang Shen,[4] Justin D. Taft,[4] Jonathan L. Torres,[1] Nelson R. Wu,[1] Baoshan Zhang,[4] Nicole A. Doria-Rose,[4] Myron S. Cohen,[5] Barton F. Haynes,[2,3] Lawrence Shapiro,[4,6] Andrew B. Ward,[1] Priyamvada Acharya,[2,3,4] John R. Mascola,[4,*] and Peter D. Kwong[4,6,8,*]

[1]IAVI Neutralizing Antibody Center, Consortium for HIV/AIDS Vaccine Development (CHAVD), Department of Integrative Structural and Computational Biology, The Scripps Research Institute, La Jolla, CA 92037, USA

[2]Duke University Human Vaccine Institute, Departments of Medicine and Surgery, Duke University School of Medicine, Durham, NC 27710, USA

[3]Center for HIV/AIDS Vaccine Immunology-Immunogen Discovery at Duke University, Durham, NC 27710, USA

[4]Vaccine Research Center, National Institute of Allergy and Infectious Diseases, National Institutes of Health, Bethesda, MD 20892, USA

[5]Departments of Medicine, Epidemiology, and Microbiology, University of North Carolina-Chapel Hill, Chapel Hill, NC 27599, USA

[6]Department of Biochemistry and Molecular Biophysics, Columbia University, New York, NY 10032, USA

[7]These authors contributed equally

[8]Lead contact

*Correspondence: jmascola@nih.gov (J.R.M.), pdkwong@nih.gov (P.D.K.)

https://doi.org/10.1016/j.celrep.2021.109922

SUMMARY

Recognition of N-linked glycan at residue N276 (glycan276) at the periphery of the CD4-binding site (CD4bs) on the HIV-envelope trimer is a formidable challenge for many CD4bs-directed antibodies. To understand how this glycan can be recognized, here we isolate two lineages of glycan276-dependent CD4bs antibodies. Antibody CH540-VRC40.01 (named for donor-lineage.clone) neutralizes 81% of a panel of 208 diverse strains, while antibody CH314-VRC33.01 neutralizes 45%. Cryo-electron microscopy (cryo-EM) structures of these two antibodies and 179NC75, a previously identified glycan276-dependent CD4bs antibody, in complex with HIV-envelope trimer reveal substantially different modes of glycan276 recognition. Despite these differences, binding of glycan276-dependent antibodies maintains a glycan276 conformation similar to that observed in the absence of glycan276-binding antibodies. By contrast, glycan276-independent CD4bs antibodies, such as VRC01, displace glycan276 upon binding. These results provide a foundation for understanding antibody recognition of glycan276 and suggest its presence may be crucial for priming immunogens seeking to initiate broad CD4bs recognition.

INTRODUCTION

Antibody-antigen interactions generally involve binding by the complementary-determining regions (CDRs) of the antibody to exposed protein surfaces on the antigen. To evade such immune recognition, many viruses evolve glycosylation to cover exposed antigenic protein surfaces (Go et al., 2008; Helle et al., 2011; Lennemann et al., 2014; Li et al., 2008; Pancera et al., 2014; Sommerstein et al., 2015; Vu et al., 2011). Viral glycosylation is often N-linked (versus O-linked) and shields potential neutralizing epitopes from immune recognition. One example is the site for CD4 binding on the HIV-1 envelope (Env) glycoprotein. The CD4 binding site (CD4bs) is surrounded by N-linked glycans at residues N197, N276, N363, and N462, as well as at N301 on an adjacent protomer in the prefusion-closed state. Immunization with Env trimer variants with these glycans deleted leads to high-titer neutralization of the viruses, in which these glycans have also been deleted; however, these high-titer responses do not neutralize viruses with these glycans present (Zhou et al., 2017).

The glycan at residue N276 (glycan276), in particular, has been found to restrict antibody access to the CD4bs, an important site of vulnerability targeted by many broadly neutralizing antibodies isolated from chronically HIV-infected patients (Scheid et al., 2011; Wu et al., 2010, 2011; Zhou et al., 2015). Glycan276 is mostly conserved and is present in over 90% of circulating HIV-1 strains. Neutralization of strains containing glycan276 has been a challenge for VRC01-class broadly neutralizing antibodies that are being considered as templates for HIV-1 vaccine (Briney et al., 2016; Tian et al., 2016). Broadly neutralizing VRC01-class antibodies elicited during the course of natural infection do evolve to overcome obstruction by glycan276,

This is an open access article under the CC BY license (http://creativecommons.org/licenses/by/4.0/).

although they generally have improved neutralization against viral strains missing this glycan.

However, a few antibodies isolated from HIV-infected patients are dependent on glycan276 for neutralization. Examples are CD4bs antibodies CAP257-RH1 (Wibmer et al., 2016), 179NC75 (Freund et al., 2015), and HJ16 (Balla-Jhagjhoorsingh et al., 2013). N276D mutation on Env trimer confers HJ16 resistance to the otherwise HJ16-sensitive viruses. In addition to CD4bs broadly neutralizing antibodies, the glycoprotein 120 (gp120)/gp41 interface antibody 8ANC195 also depends on glycan276 for neutralization (Scharf et al., 2014). The mechanism by which these antibodies recognize glycan276 and depend on the binding of glycan276 for neutralization is still unclear.

Here we report the isolation and characterization of two lineages of glycan276-dependent CD4bs antibodies from HIV-positive donors. We determined structures of a representative member from each of these lineages and also the structure of 179NC75, a previously identified glycan276-dependent CD4bs antibody, in complexes with Env trimer using single-particle cryo-electron microscopy (cryo-EM). Analysis of these structures, in comparison with those of other glycan276-dependent and -independent antibodies, provided insight into both the structural basis for glycan276 recognition and a mechanism for glycan276-antibody dependency.

RESULTS

Identification of glycan276-dependent CD4bs broadly neutralizing antibodies

To identify a donor with glycan276-dependent broadly neutralizing antibodies, we screened donor sera from a Center for HIV/AIDS Vaccine Immunology (CHAVI) cohort by neutralization fingerprint analysis (Georgiev et al., 2013). Two donors, CH314 and CH540, showed signatures that correlated strongly with previously identified glycan276-dependent antibodies: HJ16 (Balla-Jhagjhoorsingh et al., 2013; Corti et al., 2010) and 8ANC195 (Chuang et al., 2013) (Figure S1A). The CH314 sera correlated especially well with these glycan276-dependent antibodies, with a combined total score of 77% (46% for HJ16-like and 31% for 8ANC195-like), while for CH540, the correlation was only 56% (36% for HJ16-like and 20% for 8ANC195-like).

We further tested CH540 serum for neutralization against wild-type BG505 and Q168.a2 viruses and their corresponding glycan276-knockout mutants (Figure S1B). Mutant viruses with glycan276 knockout had substantially reduced sensitivity to serum neutralization. In addition, serum neutralization activity was depleted when the serum was preincubated with the HxB2 gp120 core protein (2CC) containing the CD4bs-knockout mutation D368R (2CC-D368R) (Dey et al., 2009; Zhou et al., 2007), but not when preincubated with the protein with an additional mutation knocking out glycan276 (2CC-D368R/N276D) (Figure S1C). These results confirmed the presence of glycan276-dependent antibodies in the CH540 serum.

To isolate the glycan276-dependent antibodies from the sera, we carried out single-cell sorting of peripheral blood mononuclear cells (PBMCs) for cells that bound to BG505 SOSIP.664 and 2CC-D368R, but not to 2CC-D368R/N276D (Figures S1D and S1E). From donor CH314, a single heavy- and light-chain pair was amplified, cloned, and expressed as the monoclonal antibody CH314-VRC33.01 (named by donor-lineage.clone and referred to as VRC33.01 from this point on). VRC33.01 used the VH4-34*02 and VK1-5*03 alleles and showed moderate somatic hypermutation (SHM) (~10% at the nucleotide level) (Figures 1A and 1B; Table S1). VRC33.01 neutralized 45% of the strains in a 208-pseudovirus panel representing all major circulating clades of HIV-1 (Chuang et al., 2019) with geometric mean half-maximum inhibitory concentration (IC_{50}) of 1.65 μg/mL (Figure 1C, left; Table S2).

From donor CH540, four heavy- and light-chain pairs were amplified from the collected single B cells, cloned, and expressed as monoclonal antibodies CH540-VRC40.01, CH540-VRC40.02, CH540-VRC40.03, and CH540-VRC40.04 (named for donor-lineage.clone and referred to as VRC40.clone from this point on). All antibodies belonged to the same clonal family and used the VH1-2*06 immunoglobulin heavy-chain variable (IGHV) allele and the VK3-15*01 immunoglobulin variable kappa (IGKV) allele (Figure 1A; Table S1). In addition, all had a five-residue insertion in the heavy-chain framework 3 region (FR H3) (Figure 1B) and were more somatically mutated compared with VRC33.01 (Figures 1A and 1B). The most potent VRC40-lineage antibody VRC40.01 neutralized 81% of viruses on the 208-pseudovirus panel with a geometric mean IC_{50} of 0.073 μg/mL (Figure 1C, right; Table S2).

To confirm neutralization dependence on glycan276 and to map the neutralization profiles of the isolated antibodies, we compared VRC33.01 and VRC40.01 with HJ16, 8ANC195, and VRC01 for neutralization on a panel of Q168.a2 mutants (Figure 1D). Both VRC33.01 and VRC40.01 were unable to neutralize glycan276-knockout mutants (mutations N276A and T278A), similar to HJ16. 8ANC195 also showed greatly reduced neutralization against glycan276-knockout mutants. By contrast, VRC01 exhibited ~3-fold improved neutralization against glycan276-knockout mutants. Overall, VRC33.01 had a neutralization profile most similar to HJ16, with sensitivity to mutations N280A, R456W, and N462A, whereas VRC40.01 was only slightly sensitive to the N462A mutation, but not to other mutations (Figure 1D). Binding of VRC40.01 to HxB2 2CC core or YU2 gp120 could be competed by known CD4bs antibodies, as well as by VRC33.01, suggesting that VRC40.01 and VRC33.01 had epitopes overlapping with each other and with those of CD4bs antibodies (Figure S2A). Deletion of glycan276 (N276D mutation) from HxB2 2CC abolished binding to VRC33.01 and VRC40.01, as did the V5 mutation R456S (Figure S2B). VRC33.01 binding was sensitive to deletion of glycan234 (N234S) or the V5 mutation N460P (Figure S2B), although its neutralization of Q168.a2 was not affected by glycan234 or N460A mutations (Figure 1D). Both VRC33.01 and VRC40.01 were sensitive to the D368R mutation, with VRC33.01 being more sensitive to the mutation (Figure S2B). This observation suggested that selection for positive binding to 2CC-D368R is perhaps not optimal for isolating glycan276-dependent CD4bs antibodies, even though we were successful in isolating VRC33 and VRC40 lineage antibodies.

A Genetic information of antibody clones identified from antigen-specific sorting of PBMCs from donors CH540 and CH314

Donor	Antibody	Heavy chain IGHV	IGHJ	CDR H3	VH mutation frequency (nt)	Light chain IGKV	IGKJ	CDR L3	VK mutation frequency (nt)
CH314	VRC33.01	4-34*02	4*02	GKRYSASYSNYFGV	10%	1-5*03	2*03	QHYMADPR	11%
CH540	VRC40.01	1-2*06	4*02	VVDGFNAAGPLEF	26%	3-15*01	5*01	QQDYYWPVT	19%
	VRC40.02	1-2*06	4*02	VVDGFNAAGPLEF	26%	3-15*01	5*01	QQDYYWPVT	19%
	VRC40.03	1-2*06	4*02	VVDGFNAAGPLEF	28%	3-15*01	5*01	QQDYGWPVT	19%
	VRC40.04	1-2*06	4*02	VVDGFNAAGPLEF	25%	3-15*01	5*01	QQDKYWPVT	19%

B Heavy-chain amino-acid sequences of CH314-VRC33 and CH540-VRC40 lineage antibodies

```
              ---------------FR1------------_CDR1-----FR2------_____CDR2______--------------FR3---------------____CDR3_____----FR4----
              1                    22       31  36             5052A          66                      82ABC        92   100ABCDEF103        113
              |                    |        |   |              | ||           |                       ||||         |      ||||||   |          |
IGHV4-34*02   QVQLQQWGAGLLKPSETLSLTCAVYGGSFSGYYWSWIRQPPGKGLEWIGEINHSGSTNYNPSLKSRVTISVDTSKNQFSLKLSSVTAADTAVYYCAR
IGHJ4*02                                                                                                              YFDYWGQGTLVTVSS
CH314-VRC33.01 ......................L..R.LN.N.......S................YF...F....AM......S......N.......GI.F...GKRYSASYSN..GV.....Q.....

              ---------------FR1------------_CDR1-----FR2------_____CDR2______---------------FR3-------------------____CDR3_____----FR4----
              1                    22       31  36            5052A          66    72ABCDE        82ABC        92   100ABCDE103        113
              |                    |        |   |             | ||           |     ||||||         ||||         |      ||||||   |          |
IGHV1-2*06    QVQLVQSGAEVKKPGASVKVSCKASGYTFTGYYMHWVRQAPGQGLEWMGRINPNSGGTNYAQKFQGRVTMTRD.....TSISTAYMELSRLRSDDTAVYYCAR
IGHJ4*02                                                                                                                  YFDYWGQGTLVTVSS
CH540-VRC40.01 ....I...PQF.T.....T........I..D.LI....LV..K....L....T.A.LMYLSH..E..LILR.VVDWRTP.LG.VN...RNV....S.I.F.G.VVDGFNAAGPLEF....SP.I...
CH540-VRC40.02 ..R.M...PQL.T.....T........I..D.LI....LV..K....L....T.G.LMYLSY..E..LILR..VDWRTP.LG.V....KN.....S.I.F.G.VVDGFNAAGPLEF....SP.I...
CH540-VRC40.03 ....I...PQL.T.....T........V.AD.LI....LV..K....L....T.A.LMYLSH..E..LILR..RDWRTP.LG.L....RN.K...S.I.F.G.VVDGFNAAGPLEF....SP.I...
CH540-VRC40.04 ..R.M...T.F.T..........T...I.SD.LI....LV..K....L....T.A.LMYLSPR.E...ILR.ESSFRTP.LG.V....RN.KF..S...F.G.VVDGFNAAGPLEF....SL.I...
                                                                                   5 AA insertion
```

C Neutralization activity on a 208-strain panel

	VRC33.01	VRC40.01
Breadth IC_{50}<50 μg/ml	45%	81%
Geometric mean IC_{50}	1.65 μg/ml	0.073 μg/ml

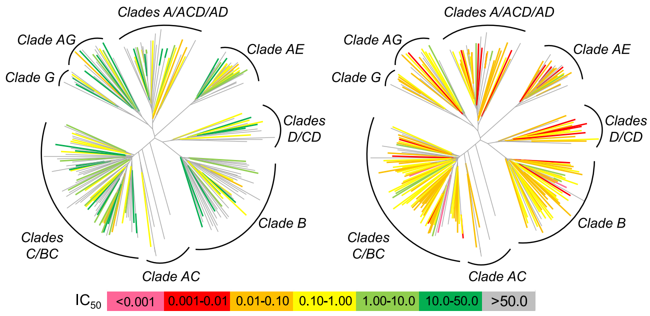

D Neutralization dependence on certain glycans and residues

	VRC33.01	VRC40.01	HJ16	8ANC195	VRC01
Q168.a2 IC_{50} (μg/ml)	0.117	0.087	0.032	0.46	0.09
Mutation	Fold change of IC_{50} relative to Q168.a2 wildtype				
N234A	1.69	1.09	1.38	>108	0.87
T236A	1.46	1.14	1.56	>108	0.80
N276A	>400	>574	>1562	22.51	0.27
T278A	>400	>574	>1562	92.42	0.30
N280A	>400	0.70	>1562	1.44	0.94
S365A	1.36	1.05	1.25	1.20	1.99
R456W	>400	1.17	15.78	0.97	1.08
G459A	0.68	0.57	>1562	1.74	0.89
N460A	1.78	0.83	6.78	1.47	1.24
N461A	1.51	0.92	4.91	1.52	1.33
N462A	50.16	8.70	38.75	1.49	3.38
S463A	1.56	1.11	2.00	1.18	1.54
G471A	1.16	0.80	1.72	1.32	1.67

Figure 1. Isolation of the glycan276-dependent antibody lineages

(A) One antibody was isolated from donor CH314, and four antibodies were isolated from donor CH540 by sorting PBMCs for 2CC-D368R+/BG505 T332N.SOSIP+/2CC-D368R-N276D− B cells. The four antibodies from CH540 belonged to the same antibody lineage.

(B) Analyses of heavy-chain amino acid sequences of CH314-VRC33 and CH540-VRC40 lineage antibodies. Framework regions (FRs) and CDRs were determined using Kabat's definition (https://www.ncbi.nlm.nih.gov/igblast/). Residue numbering was based on the Kabat numbering scheme. A 5-amino acid insertion was observed in FR3 in VRC40 lineage.

(C) Neutralization analysis. VRC33.01 neutralized 45% of the 208-strain panel and VRC40.01 neutralized 81%.

(D) Both VRC33.01 and VRC40.01 were dependent on the presence of glycan276 for neutralization. VRC33.01 neutralization was sensitive to N280A, R456W, and N462A mutations, similar to HJ16. VRC40.01 neutralization was sensitive to N462A, but not to other V5 mutations, such as R456W, G459A, N460A, and N461A, or mutations of glycan234 (N234A and T236A). VRC01 neutralized better with removal of the glycan at N276.

See also Figures S1 and S2 and Tables S1 and S2.

Structure of VRC40.01 Fab in complex with Env trimer reveals basis for glycan276 recognition

Neutralization assessment indicated VRC40.01 to neutralize most of the glycan276-bearing strains of the 208-isolate panel; to visualize how VRC40.01 interacts with glycan276, we determined a cryo-EM structure of Env trimer BG505 SOSIPv5.2 (Havenar-Daughton et al., 2016) in complex with the antigen-binding fragment (Fab) of VRC40.01 and an Env base-binding RM19R Fab (Martin et al., 2020) at 3.3 Å (Figures 2, S3, and S4; Table S3). We also determined a cryo-EM structure of BG505 DS-SOSIP (Gulla et al., 2021) in complex with VRC40.01 Fab at 3.7 Å (Figures 2, S5, and S6; Table S3). Modeling of the VRC40.01 Fab in the cryo-EM density was facilitated by a 2.83-Å crystal structure of VRC40.01 Fab (Table S4). The two cryo-EM structures revealed nearly identical interactions between the antibody and Env. The density for the constant domains of VRC40.01 in the BG505 SOSIPv5.2 complex tapered off quickly, and only the variable domains were modeled in the refined structure (Figures S3D and S3E; Table S3). Hereafter, except for figures illustrating both variable and constant domains of VRC40.01, we used the BG505

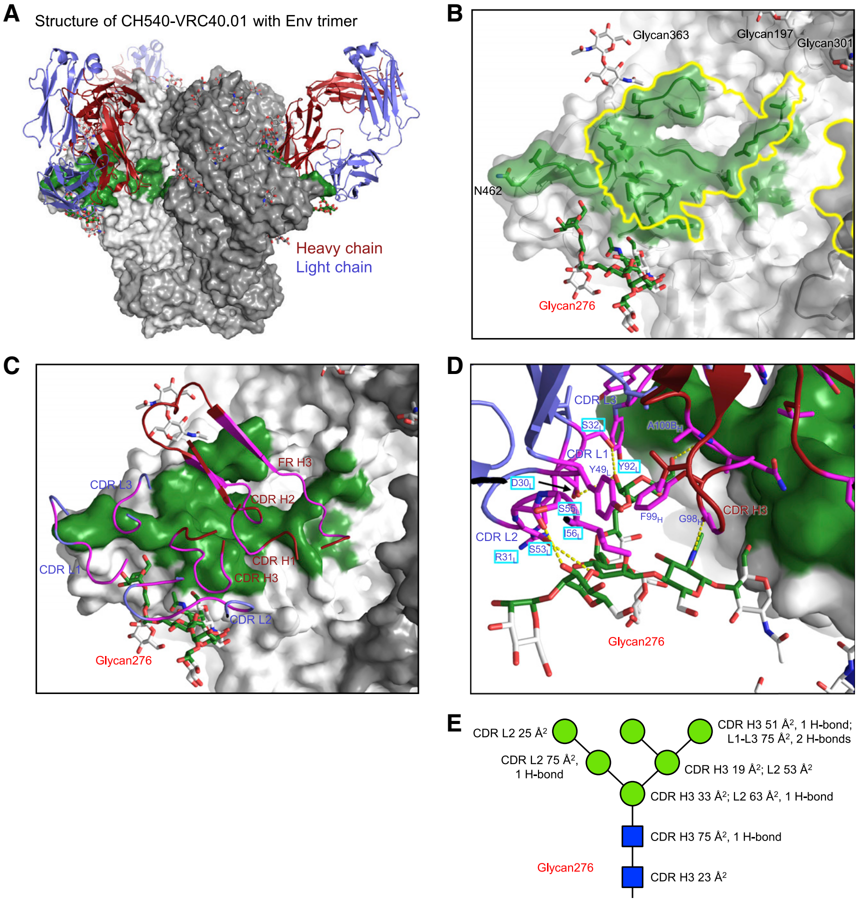

Figure 2. Structure of VRC40.01 Fab in complex with Env trimer reveals basis for its glycan276 recognition

(A) Overall structure of VRC40.01 Fab in complex with Env trimer. The antibody Fab is shown in cartoon representation with the heavy chain colored red and light chain blue, and the trimer is shown as gray surface with the VRC40.01-binding epitope colored green and the glycans in stick representation.

(B) Zoom-in of the VRC40.01-binding epitope (green) reveals substantial overlap with CD4bs (yellow outline). The epitope was defined by atoms within 5.5 Å from VRC40.01. Glycans surrounding the epitope are shown as sticks, except for the glycan at N462, which is not modeled in the cryo-EM structures. The side chain of N462 is labeled.

(C) VRC40.01 paratope binding Env trimer. Antibody residues involved in binding are colored magenta. Both heavy and light chains are involved in binding glycan276.

(D) Detailed binding interactions between VRC40.01 and glycan276. Antibody residues contacting glycan276 are labeled, with SHM residues in cyan boxes. Hydrogen bonds are shown as yellow dashed lines.

(E) Schematic showing ordered saccharide units of glycan276 in standard representation, with antibody buried surface area and hydrogen bonds for each saccharide unit delineated.

See also Figures S3–S6 and Tables S3 and S4.

SOSIP.v5.2 complex to analyze VRC40.01-Env binding because its higher resolution provided greater definition of interactions.

VRC40.01 bound an epitope overlapping the CD4bs and used both heavy and light chains to interact with glycan276 (Figures 2A and 2B). Heavy-chain interactions accounted for ~70% of the epitope. The VRC40.01 heavy chain bound at virtually the same location as the N-terminal domain of CD4 D1D2 and made extensive contact with the CD4-binding loop, the D loop, and the β23/V5/β24 region (Figures 2C and S4A). Although all three CDRs of the heavy chain interacted with gp120, interactions with CDR H2 were the most extensive. All three CDRs of the heavy chain hydrogen-bonded with residues in the D loop, with Arg50 forming a salt bridge with Asp457 in the gp120 V5 loop (Figures S4A and S4B). Residues in CDR H2 and FR H3 also contacted the CD4-binding loop, forming three hydrogen bonds (Figure S4C).

Glycan276 was recognized about equally by both heavy and light chains of VRC40.01, interacting with CDR H3 and CDR L1-L3 (Figures 2C–2E and S4D–S4F). Specifically, the CDR H3 formed interactions with the two protein-proximal *N*-acetylglucosamines and some of the mannoses, while CDR L1-L3 formed contacts with protein-distal mannoses (Figures 2D and 2E).

The five-residue FR H3 insertion contacted C1, C4, and C5 regions of gp120, with interactions including a hydrogen bond between the backbone nitrogen of $Trp72C_{HC}$ and the amide oxygen of $Gln428_{gp120}$ and a cation-π sandwich of $Trp72C_{HC}$ side chain between $Arg72D_{HC}$ and $His105_{gp120}$ (Figure S4F). The role of this FR-H3 insertion in binding was confirmed by mutating VRC40.01 Fab to remove this insertion; when compared with the wild-type VRC40.01 Fab, the deletion mutant showed reduced binding to BG505 SOSIP.664 (Figure S4G). Similar FR H3 insertions are present in some VRC01-class antibodies, such as VRC03, VRC06, and 3BNC117. Due to a difference in binding modes, the VRC01-class insertions primarily contribute to increased interactions with V2 loop in the main binding protomer and the V1V2 and V3 loops in the neighboring protomer (Figure S4I). Overall, VRC40.01 binding was dominated by its heavy chain, with CDR H2 making the largest contribution and both heavy and light chains making extensive interactions with glycan276.

Structure of VRC33.01 Fab in complex with Env trimer reveals another mode of glycan276 recognition

We next obtained a cryo-EM structure of BG505 DS-SOSIP (Gulla et al., 2021) in complex with VRC33.01 Fab at 3.7 Å resolution (Figures 3, S7, and S8; Table S3). Modeling of the VRC33.01 Fab in the cryo-EM density was facilitated by a crystal structure of unliganded VRC33.01 Fab, which we determined at 1.54 Å resolution (Table S4).

Similar to VRC40.01, VRC33.01 bound to an epitope that overlapped the CD4bs (Figures 3A and 3B). Relative to the VRC40.01 epitope, the VRC33.01 epitope was smaller and shifted toward the outer edge of gp120, with less overlap with the CD4bs. Both heavy

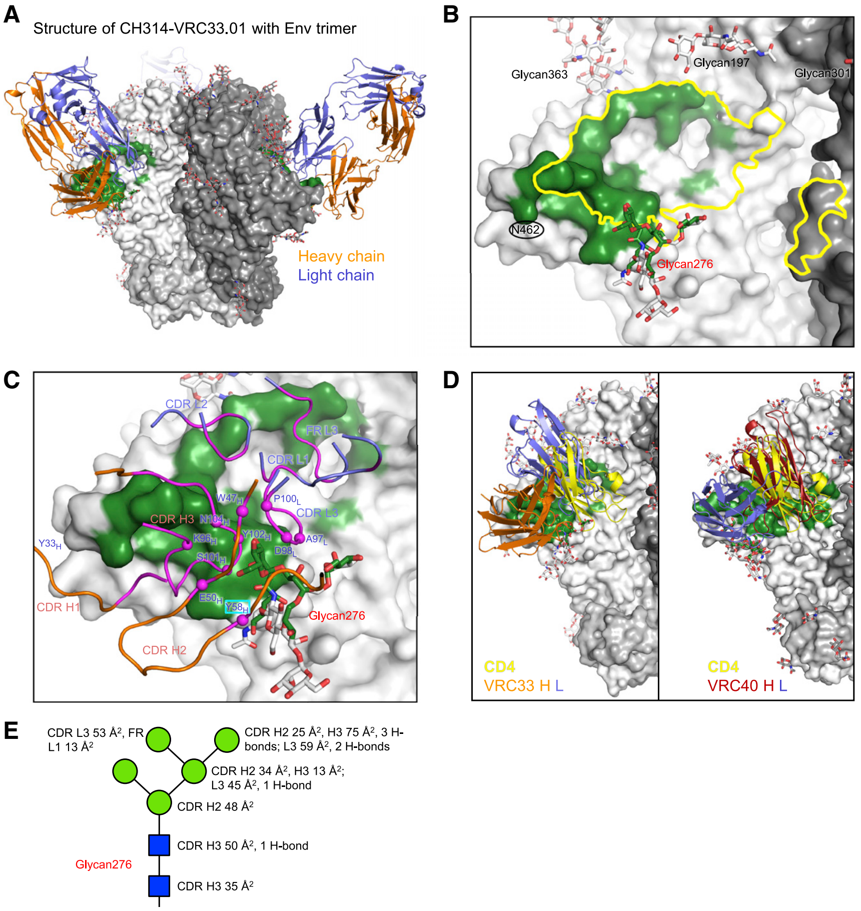

Figure 3. Structure of VRC33.01 Fab in complex with Env trimer reveals another mode of glycan276 recognition

(A) Overall structure of VRC33.01 in complex with Env trimer. VRC33.01 Fab is shown in cartoon representation in orange (heavy chain) and blue (light chain). The trimer is shown as gray surface with the antibody binding surface colored green. Each Fab interacted exclusively with a single gp120 subunit.

(B) Zoom-in view of the VRC33 epitope (green surface) revealed partial overlap with CD4bs (yellow outline). The epitope is defined by atoms within 5.5 Å from VRC33.01. N-linked glycans surrounding CD4bs are labeled. The glycan at N462 is not modeled because of lack of density.

(C) VRC33.01 paratope binding Env trimer. Glycan276 binding involved both heavy and light chains but was dominated by the heavy chain. Antibody residues involved in binding are colored magenta; those contacting glycan276 are marked with magenta spheres and labeled, with non-CDR3 SHM residues in cyan boxes.

(D) Comparison of the overall orientation of VRC40.01 and VRC33.01. Both bind in a similar site overlapping the CD4 binding site, but with heavy- and light-chain swapping positions. CD4 D1 domain is shown in yellow. Antibody constant domains are not shown for clarity.

(E) Schematic showing ordered saccharide units of glycan276 in standard representation, with antibody buried surface areas for each saccharide unit delineated.

See also Figures S7 and S8 and Tables S3 and S4.

and light chains contributed about equally to the binding interface with gp120. Heavy-chain interactions involved CDRs H1 and H3, with CDR H3 dominating the binding interaction. Light-chain interactions involved CDRs L1 and L2 and FR L3, with CDR L3 and FR L3 dominating and making contacts with the D loop and CD4-binding loop, respectively (Figure 3C). We observed a salt bridge from Arg66C of light chain to Asp368 of CD4-binding loop (Figure S8F). This salt bridge was not observed in the VRC40.01 complex, although the electron density for the Asp368 side chain was not well defined, and an alternative rotamer of Asp368 could potentially allow for a salt bridge to an arginine.

Glycan276 interacted with both heavy chain (CDR H2 and H3) and light chain (CDR L3), with the heavy chain dominating binding (Figure 3C). Notably, VRC33.01 differed considerably from VRC40.01 in the relative positions of heavy and light chains. The orientation of the heavy and light chains of VRC33.01 were approximately 180° from the orientation of the heavy and light chains of VRC40.01. As a result, the light chain of VRC33.01, instead of the heavy chain as in the case of VRC40.01, occupied the position of CD4 N-terminal domain in binding the Env trimer (Figure 3D), and recognition of glycan276 utilized a different set of atomic interactions, with the CDR H3 interacting with both *N*-acetylglucosamines and mannoses, and CDR H2 and L3 interacting mainly with mannoses (Figure 3E).

Neutralization fingerprint analysis reveals glycan276-dependent antibodies to cluster as a subgroup

We observed VRC40.01 to be broader and more potent than all other glycan276-dependent HIV-neutralizing antibodies identified thus far, including antibodies VRC33.01, HJ16, and 179NC75, as well as the gp120-gp41 interface antibody 8ANC195 (Figure 4A; Table S2). Overall, VRC40.01 was comparable in potency, although not in breadth, to the broadest VRC01-class antibodies, such as antibody 1-18 and N6 (Figure 4A). VRC40.01 was also broader than neutralizing antibodies targeting the glycan-V3 patch, the gp120-gp41 interface, or the silent face, such as PGT128, PGT151, and VRC-PG05, respectively (Figure 5B). Notably, neutralizing fingerprint analysis indicated that all glycan276-dependent broadly neutralizing antibodies clustered together as a subgroup (Figure 4C). Specifically, VRC40-lineage antibodies had nearly identical neutralization fingerprints despite their differences in neutralization breadth; HJ16 and VRC33.01 also clustered closely, consistent with their similarity in mutational analysis (Figure 1D), and these two antibodies, together with the VRC40 antibodies, closely clustered with 179NC75 as a separate branch. Despite having a very different epitope, the gp41-gp120 interface antibody 8ANC195 clustered closely with the glycan276-dependent CD4bs antibodies (Figure 4C). These observations suggested

A Neutralization statistics on 208-strain panel for glycan276-dependent and -independent antibodies

	Recognition dependent on glycan276					Recognition independent of glycan276				
	VRC40.01	VRC33.01	HJ16	179NC75	8ANC195	1-18	b12	N6	VRC01	VRC13
Breadth (IC50 ≤50 μg/ml)	81%	45%	31%	25%	67%	95%	43%	97%	90%	83%
Median IC50 (μg/ml)	0.064	1.19	0.239	1.10	0.791	0.046	1.60	0.086	0.328	0.089
Median IC80 (μg/ml)	0.283	3.26	0.580	1.17	3.24	0.151	4.46	0.238	0.622	0.51

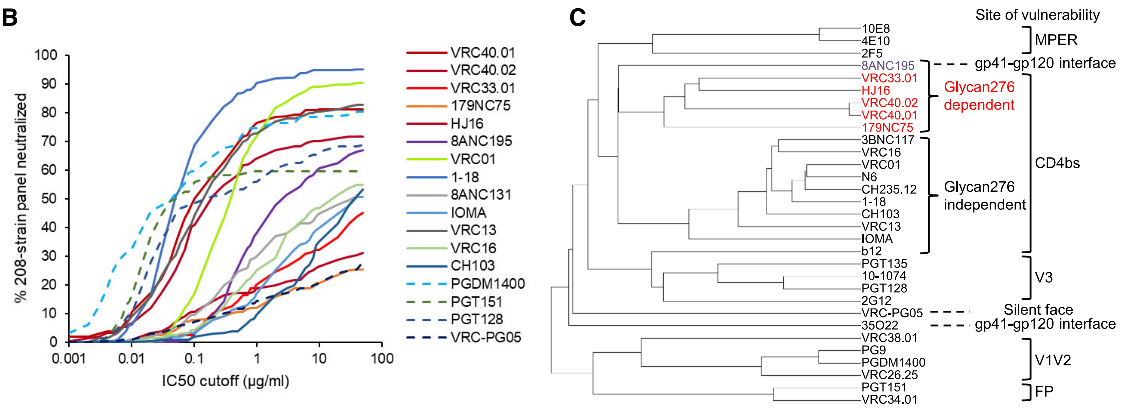

Figure 4. Neutralization fingerprint reveals glycan276-dependent antibodies, VRC33, VRC40, HJ16, and 179NC75, to cluster as a subgroup
(A) Neutralization statistics on 208-strain panel for glycan276-dependent antibodies and glycan276-independent CD4bs antibodies.
(B) Neutralization breadth-potency curves for CD4bs antibodies. The dashed lines are for non-CD4bs and non-glycan276-interacting antibodies.
(C) Neutralization fingerprint analysis revealed VRC40 and VRC33 antibodies to cluster with HJ16 and 179NC75. MPER: membrane proximal region, FP: fusion peptide.
See also Table S2.

glycan276 dependence to be a dominant feature in this particular neutralization fingerprint.

Structure of 179NC75 Fab in complex with Env trimer reveals a third mode of glycan276 recognition, distinct from that of VRC40.01 or VRC33.01

In light of the similarity in neutralization fingerprint of antibody 179NC75 with antibodies VRC33 and VRC40, we sought to define the interactions of 179NC75 with Env. Antibody 179NC75 was isolated from HIV-infected donor EB179, and in a humanized mouse model, HIV-1 YU2 virus escapes 179NC75 neutralization through a N276 mutation (Freund et al., 2015). We succeeded in determining a 4.8-Å resolution cryo-EM structure of the 179NC75 Fab in complex with a stabilized Env trimer (Rawi et al., 2020) of clade A strain Q23.17 (Figures 5, S9, and S10; Table S3). 179NC75 bound to an epitope that overlapped the CD4bs and interacted with glycan276 at the outer edge of its epitope (Figures 5A and 5B). The 179NC75 epitope was smaller than those of VRC40.01 and VRC33.01, with most of its binding interactions occurring within the outer domain of the CD4bs (Figure 5B). The 179NC75 heavy chain dominated interactions with the trimer, with its CDR H3 contributing ~75% of the paratope-surface area and interacting with loop D, β23-V5-β24, and CD4-binding loop (Figure 5C). The light-chain CDR L1 contributed ~20% of the paratope, with CDR L2 and FR L3 having minor interactions. The relative positions of the heavy and light chains were rotated ~90° from those of VRC40.01 (Figure 5D). Consequently, glycan276 interactions were contributed solely by heavy-chain CDRs H1 and H3 (Figure 5C). Overall, the 179NC75-Env trimer complex structure presents yet another mode of recognition for glycan276-dependent antibodies.

Structural comparison of glycan276-dependent and -independent CD4bs antibodies delineates the mechanism of glycan dependence

As described above, VRC40.01, VRC33.01, and 179NC75 bound the CD4bs with overlapping epitopes, and all showed substantial interactions with glycan276. However, the binding modes of these antibodies were different, with heavy- and light-chain binding at different locations and interacting with glycan276 by using different sets of residues. Could analysis of these glycan276-dependent antibodies nonetheless lend general insight into their dependency?

The epitopes of VRC40.01, VRC33.01, and 179NC75 overlapped substantially, not only with each other but also with other glycan276-dependent CD4bs antibodies, such as CAP257-RH1 and HJ16, as well as glycan276-independent CD4bs antibodies, such as VRC01, VRC13, VRC16, and 1-18 (Figure 6A). Compared with the epitopes of HJ16, VRC33.01, and CAP257-RH1, the VRC40.01 epitope shifted toward the CD4-binding loop and away from the V5 loop (Figure 6A), and we observed a general trend of higher neutralization breadth and potency for CD4bs antibodies with epitopes closer to the neighboring gp120-protomer interface, such as 1-18, VRC01, and VRC40.01, than those with epitopes recognizing regions more

CellPress
OPEN ACCESS

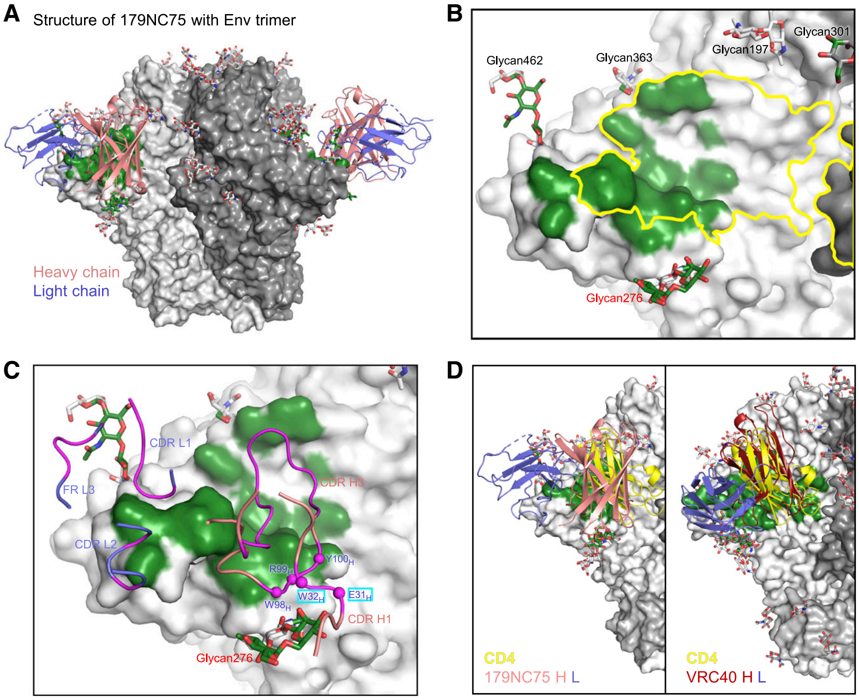

Figure 5. Structure of 179NC75 Fab in complex with Env trimer reveals a third mode of glycan276 recognition

(A) Overall structure of 179NC75 Fab in complex with Env trimer. The antibody is shown as cartoon representation in salmon (heavy chain) and light blue (light chain), and trimer as gray surface with 179NC75-binding surface colored green. Only the variable domains were modeled.

(B) Epitope of 179NC75 mapped on the trimer surface, defined by atoms within 5.5 Å from the antibody. The epitope was relatively small, resided exclusively in one gp120 subunit, and substantially overlapped with CD4bs (yellow outline). N-linked glycans surrounding CD4bs, at residues N197, N276, N301, N363, and N462, are labeled.

(C) 179NC75 paratope binding Env trimer. Antibody residues involved in binding are colored magenta; those contacting glycan276 are marked with magenta spheres at the Cα position and labeled, with non-CDR3 SHM residues in cyan boxes. The heavy chain of 179NC75 dominated the interactions with the trimer and bound glycan276 exclusively.

(D) Comparison of the overall orientation of VRC40.01 and 179NC75. CD4 D1 domain is also shown in yellow for comparison. Only variable domains are shown for clarity. The antibody bound at a site similar to that of VRC40.01, but with the heavy- and light-chain positions rotated ~90° relative to those of VRC40.01.

See also Figures S9 and S10 and Table S3.

distal to the trimer 3-fold axis (and more focused on the gp120 outer domain), such as 179NC75, HJ16, and CAP257-RH1. However, this trend did not apply to antibodies that did not interact with glycan276, such as VRC16. Moreover, although dependent on glycan276 for neutralization, 8ANC195 bound to an epitope on the opposite face of glycan276 recognized by CD4bs antibodies, and its epitope had minimal overlap with the epitope of VRC40.01 (Figure 6A).

To quantify antibody approach orientation, we calculated the antibody approach vector, herein defined by the vector from the centroid of the antibody epitope to the center between the two conserved disulfides in the antibody variable domains. Notably, all approach vectors of the glycan276-dependent antibodies clustered with similar latitudinal angles (the angle between the antibody approach vector and the Env trimer axis) and longitudinal angles (the angles between the antibody approach vector and the protomer axis, defined as a line passing through the centroid of CD4bs, perpendicular and intersecting the trimer axis) (Figures 6B, 6C, and S11). The center of the vector cluster was about halfway between glycans at N276 and N363, extending toward the V5 region.

The approach vectors of glycan276-independent antibodies that utilized CDR H3-dominated modes of recognition, such as VRC16, VRC13, and CH103, clustered together close to the CD4-binding loop (Figures 6B and S11). These antibodies bound Env at locations away from glycan276 and had relatively small latitudinal angles; thus, they did not interact with glycan276, explaining their lack of dependence on this glycan for neutralization.

The approach vectors of glycan276-independent antibodies that utilize specific V-genes for recognition, including those that use VH1-2 (the VRC01-class antibodies, such as VRC01, N6, and 3BNC117) and those that use VH1-46, such as antibody 1-18 (Schommers et al., 2020), also formed a separate cluster, although in this case the cluster was located closer to glycan276 than the other CD4bs antibodies. Structures of these antibodies with glycosylated trimer reveal substantial antibody contact with glycan276. Interestingly, despite this glycan recognition, mutational analysis indicated that removal of glycan276 improved VRC01 neutralization (Figure 1D).

To address why some antibodies, such as VRC01, favor glycan276-free Env trimers, whereas others, such as VRC40.01, depend on glycan276 for neutralization, we analyzed the conformation and binding interactions of glycan276 in these structures of CD4bs antibodies in complex with Env trimer or gp120, by superposing the structures based on the gp120 subunit. We included in the analysis both glycan276-dependent and -independent antibodies, as well as Env structures free of glycan276-binding antibodies (Figures 6D and S11). We observed that the binding of glycan276-dependent antibodies maintained the same orientation of glycan276 as observed in the structures absent of glycan276-interacting antibodies (PDB: 5FYL and 5FUU), as well as in the CD4-bound structure (PDB: 5VN3). A similar observation has been reported from comparison of crystal structures of gp120 in complex with CAP257-RH1, HJ16, and 8ANC195 (Wibmer et al., 2016). In contrast, binding of antibody VRC01 substantially displaced glycan276

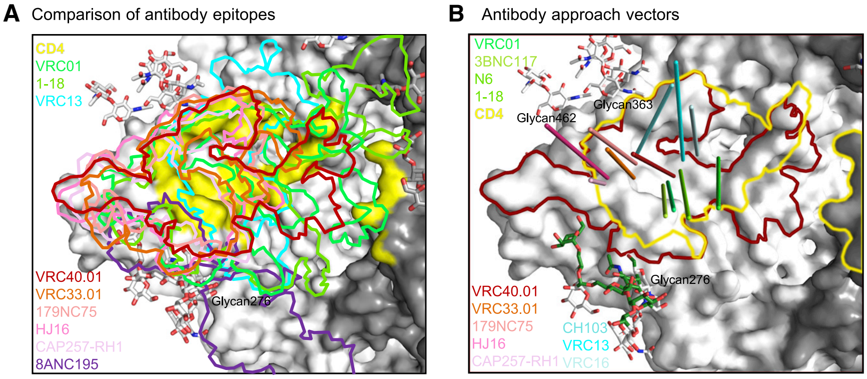

C Antibody approach vector latitudinal and longitudinal angles

	Glycan276-dependent antibodies					Glycan276-independent antibodies						
	VRC40 .01	VRC33 .01	179NC75	HJ16	CAP257 RH1	VRC01	3BNC 117	1-18	N6	VRC13	CH103	VRC16
Latitudinal	76	72	74	63	80	94	92	98	100	50	54	66
Longitudinal	57	63	60	53	67	71	70	66	72	71	90	67

D

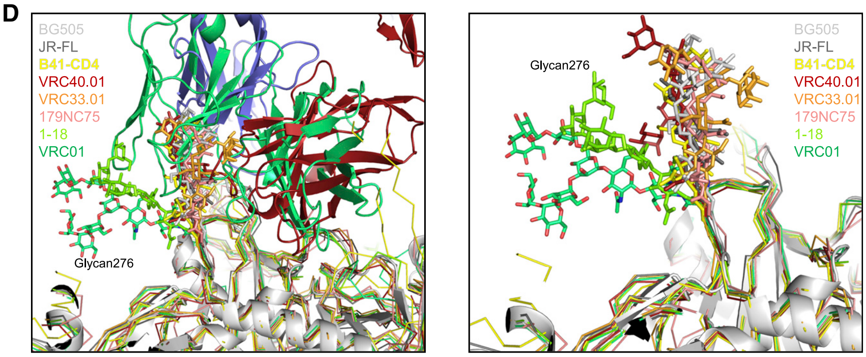

Figure 6. Glycan276-dependent and -independent CD4bs antibodies induce different orientations of glycan276

(A) Mapping of glycan276-dependent and -independent antibody epitopes on the Env trimer surface of the VRC40.01 complex. Glycans are shown in sticks. CD4bs is shown as yellow surface.

(B) Antibody approach vectors of glycan276-dependent and -independent antibodies viewed relative to the VRC40.01 epitope (outlined in red) and CD4bs (outlined in yellow). The approach vector for each antibody is defined as a line from the center of its epitope to the center of the variable domain, which is defined as the center between the two conserved disulfides. The glycan276-dependent antibodies, VRC40.01, VRC33.01, 179NC75, HJ16, and CAP257-RH1, had similar approach angles that allowed them to have optimal interactions with glycan276. VRC01 tilted toward glycan276, whereas some other glycan276-independent antibodies, VRC13, VRC16, and CH103, faced away from the glycan and did not interact with it.

(C) Antibody approach angles calculated as latitudinal angle (to the trimer axis) and longitudinal angle (to the protomer axis, defined as a line passing through the centroid of CD4bs, perpendicular and intersecting the trimer axis).

(D) (Left) Structural alignment revealed glycan276-dependent antibodies to retain the glycan in a conformation similar to those in the absence of glycan276-binding antibodies (BG505, PDB: 5FYL; JR-FL, PDB: 5FUU) and in the B41-CD4 complex (PDB: 5VN3), whereas VRC01 binding pushed glycan276 to a different conformation (PDB: 5FYJ), presumably in an elevated energy state. VRC01 heavy and light chains are shown in green; VRC40 heavy chain is in fire-brick and light chain in light blue. Glycans other than glycan276, other antibodies, and CD4 are not shown for clarity. (Right) Zoom-in view showing only glycan276 for clarity.
See also Figure S11.

away from the CD4bs (PDB: 5FYJ) (Figures S11A and S11B). As a consequence of the displacement of glycan276, glycan234 in the VRC01 complex also moved to avoid clashing with glycan276. Such movements of the glycans from their prevalent positions (their low-energy states) likely engender energetic penalties. Binding of antibody 1-18 also displaced glycan276 (PDB: 6UDJ), but to a lesser extent that did not require additional movement of glycan234, possibly because the center of the 1-18 epitope was farther away from glycan276, extending toward the protomer interface (Figures 6B, S11A, and S11C). For CD4bs antibodies with little to no glycan276-interaction or dependency, such as CH103 (PDB: 4JAN), VRC13 (PDB: 4YDJ), and VRC16 (PDB: 4YDK), the orientation of glycan276 was the same as that in the absence of CD4bs antibodies, including that in the CD4-Env complex.

The results above from integrating antibody approach vectors and glycan276 orientations suggest that glycan276-dependent antibodies interact substantially with glycan276 but in a manner that maintains the orientation of glycan276 in the same conformation as those in the absence of glycan276-binding antibodies. Meanwhile, antibodies that bind better in the absence of glycan276 displace this glycan to bind. Lastly, antibodies with no dependence on glycan276, such as CH103 (PDB: 4JAN), VRC13 (PDB: 4YDJ), and VRC16 (PDB: 4YDK), are far enough away from this glycan to have minimal interaction. In addition, we note that even though not a CD4bs antibody, 8ANC195 binding also maintains the same orientation of glycan276 (Figure S11D). The above analysis suggests that to have positive contributions to the overall binding energy, the antibody binding to a glycan must not displace the glycan conformation from its lowest energy state, i.e., the conformation of the glycan free of interacting ligands. Overall, our results emphasize the importance of accounting for movements of the atoms from their favored, i.e., low-energy state, positions when performing energetic calculations.

Ontogeny of glycan276-dependent antibodies, except for VRC40.01, is similar to those of the CDR H3-dominated subcategory of antibodies

Glycan276-dependent antibodies identified previously showed recognition that was CDR H3 dominated and not VH-gene restricted (Balla-Jhagjhoorsingh et al., 2013; Zhou et al., 2015). Because CDR H3 recognition depends on V(D)J recombination, it is often less reproducible than recognition that is VH-gene

CellPress
OPEN ACCESS

A BSA (Å^2) analyses for CDRs and FRs of CD4bs antibodies in complex with Env trimer

Antibody	Env contacts	H1	H2	H3	FR H	H total	L1	L2	L3	FR L	L total	Ab total
VRC40.01	Protein	74	434	115	342	964	6	0	154	0	160	1124
	Glycan276	0	0	200	0	200	86	183	23	0	292	492
	Protein+276	74	434	315	342	1164	92	183	177	0	452	1616
VRC33.01	Protein	15	0	416	0	431	87	177	5	208	477	908
	Glycan276	0	115	176	0	291	6	0	165	13	184	475
	Protein+276	15	115	592	0	722	93	177	170	221	661	1383
179NC75	Protein	0	0	455	0	455	108	35	0	0	143	598
	Glycan276	72	0	181	0	253	0	0	0	0	0	253
	Protein+276	72	0	636	0	708	108	35	0	0	143	851
HJ16	Protein	31	126	457	0	614	149	0	209	0	358	972
PDB: 4YE4	Glycan276	0	0	0	0	0	60	0	0	0	60	60*
	Protein+276	31	126	457	0	614	209	0	209	0	418	1032
8ANC195	Protein	138	95	595	256	1084	264	57	4	20	346	1429
PDB: 6NQD	Glycan276	175	0	15	190	380	0	0	0	0	0	380
	Protein+276	313	95	609	446	1464	264	57	4	20	346	1809
CH103	Protein	33	105	357	0	495	67	119	72	106	364	859
PDB: 4JAN	Glycan276	0	0	0	0	0	0	0	0	0	0	0*
	Protein+276	33	105	357	0	495	67	119	72	106	364	859
VRC13.01	Protein	343	21	587	7	958	24	0	0	0	24	982
PDB: 4YDJ	Glycan276	0	0	0	0	0	0	0	0	0	0	0*
	Protein+276	343	21	587	7	958	24	0	0	0	24	982
VRC16.01	Protein	0	167	667	77	910	103	41	112	41	296	1207
PDB: 4YDK	Glycan276	0	0	0	0	0	0	0	0	0	0	0*
	Protein+276	0	167	667	77	910	103	41	112	41	296	1207
VRC01	Protein	43	680	145	138	1006	16	0	171	22	209	1215
PDB: 5FYJ	Glycan276	0	0	8	0	8	181	0	14	165	360	368
VH1-2	Protein+276	43	680	153	138	1014	197	0	185	187	569	1583
1-18	Protein	80	656	231	131	1098	5	0	135	39	180	1278
PDB: 6UDJ	Glycan276	0	0	0	0	0	232	0	20	327	580	580
VH1-46	Protein+276	80	656	231	131	1098	238	0	156	366	760	1858

* The crystal structures of HJ16, CH103, VRC13.01, and VRC16.01 have gp120 partially deglycosylated; only one saccharide unit of glycan276 is modeled in each of the structures.

B B-cell ontogenies of glycan276-dependent HIV-1 neutralizing antibodies

Antibody type	Antibody	V_H gene	CDR H3 length	V_L gene	CDR L3 length	CDR with largest BSA	Maturation SHM	Sequence signatures	Frequency
CD4-binding site	HJ16	VH3-30	19	VK4-1	8	H3	High SHM	HC:^{99}HKNGAVDFFS<br>LC:^{91}TRWT	< 1.0E-25
	VRC33.01	VH4-34	14	VK1-5	8	H3	Median SHM	HC:^{94}RxKRxSASYSNY<br>LC:^{93}AD	1.42E-22
	VRC40.01	VH1-2	13	VK3-15	9	H2	High SHM	HC:^{98}GFNA<br>LC:^{92}YxW	7.72E-13
	179NC75	VH3-21	24	VL3-1	10	H3	High SHM	HC:^{96}H,<br>^{100}WxRRSPVEKV	2.69E-21
Interface	8ANC195	VH1-69	20	VK1-5	9	H3	High SHM	HC:^{97}ExRW,^{100E}HD	7.90E-15

C

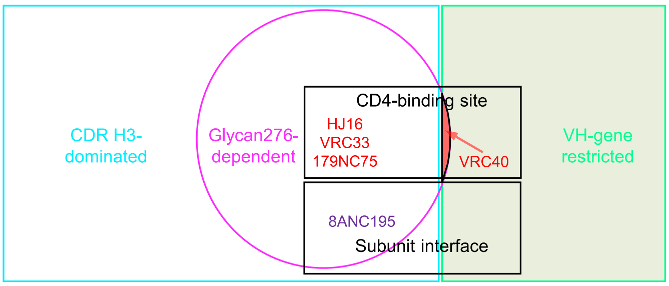

Figure 7. Ontogenies of glycan276-dependent antibodies, except for VRC40, are similar to those of the CDR H3-dominated subcategory of antibodies

(A) Analyses of binding interface for glycan276-dependent and -independent antibodies in complex with Env trimer or gp120 core. Buried surface areas were calculated with PISA, utilizing cryo-EM or crystal structures with accession codes as listed. Entries for which CDR dominates binding interface are highlighted in red. FR BSA is the sum for each chain; in most antibodies, only FR3 contributed.

(B) B cell ontogeny-related characteristics of glycan276-dependent antibodies.

(C) Venn diagram showing relationship of glycan276-dependent antibodies with various antibody categories.

restricted, with the latter being the focus of most vaccine development efforts. To define the overall mode of interaction, we analyzed the CDR contributions of each antibody to the buried surface areas (BSAs) at the antibody-antigen binding interface (Figure 7A). All of the glycan276-dependent antibodies showed CDR H3-dominated BSAs, except for antibody VRC40.01, which exhibited CDR H2-dominated interface. For VRC40.01, CDR H2 accounted for the largest BSA (434 Å^2), and the CDR H3 contributed only 115 Å^2 of 954 Å^2 total BSA for the heavy-chain contacts to the protein portion of the trimer. Including its BSA with glycan276, VRC40.01 CDR H3 accounted for only approximately one-fourth of the heavy-chain total BSA. For all other known glycan276-dependent antibodies, VRC33.01, 179NC75, HJ16, and even the subunit interface antibody 8ANC195, CDR H3 contributed the largest BSA to the antibody paratopes (Figure 7A). This indicated that VRC33.01, 179NC75, HJ16, and 8ANC195 to belong to the CDR H3-dominated category.

By contrast, VRC40.01 was more similar to antibodies VRC01 and 1-18, with interfaces dominated by CDR H2 contributions. Could VRC40 mode of recognition be reproducibly elicited? VRC40 lineage was derived from VH1-2 germline gene, and its recombination frequency was substantially higher than other glycan276-dependent CD4bs broadly neutralizing antibodies (Figure 7B). Also, the germline revertant of VRC40.01 did not bind BG505 Env trimer, whereas the mature antibody bound the trimer at an apparent K_D of 60.2 ± 5.1 nM (Figure S4H). These characteristics indicated that VRC40.01 belonged to the VH-gene restricted category, unique in the known glycan276-dependent antibodies (Figure 7C). Unlike VRC01 and 1-18, however, VRC40.01 CDR H3 contributed substantially to the binding of glycan276 (200 Å^2 BSA), with the total contributions of both CDR L3 and H3 (total of 492 Å^2) similar to that from CDR H2 (434 Å^2). This suggests that in addition to specific VH-gene, the VRC40.01 recognition requires specific recombination-derived elements, including ^{98}GFNA in the heavy chain and ^{92}YXW in the light chain (Figure 7B). Thus, antibody VRC40.01 likely belongs to VH-gene-restricted but with some characteristics of CDR H3-dominated modes of recognition.

DISCUSSION

CD4bs-directed broadly neutralizing antibodies can be divided into two categories, those that bind Env mainly through interaction with their CDR H3 (CDR H3 dominated) or those that use the VH1-2 or VH1-46 immunoglobulin variable heavy genes

(VH-gene restricted) (Zhou et al., 2015). CDR H3-dominated CD4bs antibodies use a variety of VH genes but depend on residues from V(D)J recombination or somatic hypermutation for binding Env, making CDR H3-dominated antibodies less reproducible. Almost all glycan276-dependent broadly neutralizing antibodies identified so far belong to the CDR H3-dominated category (Zhou et al., 2015). However, VRC40.01, the most potent and broadest glycan276-dependent antibody, exhibits characteristics of the VH-gene restricted category, with a recombination frequency higher than other glycan276-dependent antibodies. Therefore, VRC40.01 could be a potential vaccine template antibody to design immunogens targeting CD4bs, complementing VRC01-class antibodies for overcoming the glycan276 barrier.

The VRC01-class antibodies include the highly potent VRC01 (Zhou et al., 2010), 3BNC117 (Scheid et al., 2011), and N6 (Huang et al., 2016) that are under clinical development for HIV treatment and prevention (ClinicalTrials.gov: NCT02716675, NCT02446847, and sNCT03538626). There have been extensive efforts to develop immunogens that can induce and activate the VH1-2 class lineage precursor B cells (Abbott et al., 2018; Havenar-Daughton et al., 2018; Jardine et al., 2015, 2016; McGuire et al., 2013; Medina-Ramírez et al., 2017). By considering features that allow glycan276-dependent broadly neutralizing antibodies to interact with glycan276, it might be possible to induce glycan276-dependent antibodies (Bonsignori et al., 2018; Chen et al., 2021; Kong et al., 2016; Parks et al., 2019; Umotoy et al., 2019) and thus to improve the breadth and potency of the elicited antibody responses.

With a few exceptions, such as IOMA, which has an eight-residue CDR L3 (Gristick et al., 2016), nearly all VH1-2 class HIV-1 neutralizing antibodies have a short five-residue CDR L3 that allows the antibodies to sterically accommodate glycan276 (Zhou et al., 2015). VH1-46-restricted CD4bs antibodies, including CH235.12, 8ANC131, and the recently described 1-18, do not require a short five-residue CDR L3 to accommodate glycan276 (Bonsignori et al., 2016; Scheid et al., 2011; Schommers et al., 2020). As shown in our analysis above, binding of these VH-gene-restricted antibodies pushes glycan276 away from its antibody-free conformation, likely resulting in an energetic penalty, and removal of glycan276 enhances binding and neutralization by these glycan276-accommodating antibodies.

VRC40.01 has most of the characteristics of VH-gene-restricted ontogeny; it is derived from VH1-2 germline gene, and its binding interface to Env is dominated by CDR H2. However, VRC40.01 does not accommodate glycan276 with a short CDR L3, typical of VRC01-class antibodies, to gain access to CD4bs (West et al., 2012; Zhou et al., 2013). Instead, VRC40.01 circumvents the obstruction of glycan276 by approaching Env with a different angle to have favorable interactions with the glycan without displacing its orientation, thereby depending on glycan276 for binding and neutralization. Overall, results from this study suggest that although most glycan276-dependent antibodies are CDR H3 restricted, B cells of other ontogeny categories can evolve to overcome the glycan obstacle and achieve broad and potent neutralization. Our findings confirm the antigenic relevance of the specific glycan conformations observed in cryo-EM and crystal structures, indicating that glycans should be considered as part of the antigenic structure of Env that needs to be mimicked in vaccine immunogens.

STAR★METHODS

Detailed methods are provided in the online version of this paper and include the following:

- KEY RESOURCES TABLE
- RESOURCE AVAILABILITY
  - Lead contact
  - Materials availability
  - Data and code availability
- EXPERIMENTAL MODEL AND SUBJECT DETAILS
  - Serum Samples
  - Cell Lines
- METHOD DETAILS
  - Fluorescence Activated Cell Sorting
  - Single B Cell RT-PCR and Cloning
  - Env Protein Production and Purification
  - Antibody Preparation
  - Env-Pseudovirus Neutralization Assays
  - Neutralization Depletion Assay
  - Neutralization Fingerprinting Analysis
  - Enzyme-Linked Immunosorbent Assay (ELISA)
  - Bio-Layer Interferometry (BLI)
  - Crystallization and Structural Determination of Antibody Fab
  - Cryo-EM Sample Preparation and Data Collection
  - Atomic Model Building and Refinement
  - Analysis of Antibody Approach Vectors and Angles
  - Analysis of the HIV-1 Env Trimer-Antibody Interface
  - Sequence Signature and Recombination Frequency
- QUANTIFICATION AND STATISTICAL ANALYSIS

SUPPLEMENTAL INFORMATION

Supplemental information can be found online at https://doi.org/10.1016/j.celrep.2021.109922.

ACKNOWLEDGMENTS

We thank M.C. Nussenzweig for providing the 179NC75 sequence, J. Stuckey for assistance with figures, members of the VRC Production Program for providing BG505 DS-SOSIP protein, and members of the Virology Laboratory and Vector Core, Vaccine Research Center for discussions and comments on the manuscript. We thank J. Baalwa, D. Ellenberger, F. Gao, B. Hahn, K. Hong, J. Kim, F. McCutchan, D. Montefiori, L. Morris, E. Sanders-Buell, G. Shaw, R. Swanstrom, M. Thomson, S. Tovanabutra, C. Williamson, and L. Zhang for contributing the HIV-1 envelope plasmids used in our neutralization panel, and N. Jean-Baptiste, R. Carroll, B. Flach, K. McKee, C. Moore, G. Padilla, J. Rathmann, S. O'Connell, S. O'Dell, S.D. Schmidt, C. Whittaker, and A.B. McDermott for assistance with neutralization assessments on the 208-strain panel. This work was supported by the Intramural Research Program of the Vaccine Research Center, National Institute of Allergy and Infectious Diseases (NIAID); the Scripps CHAVD (UM1 AI44462) (to A.B.W.); NIH F31 Ruth L. Kirschstein Predoctoral Award AI131873 (to C.A.C.); Achievement Rewards for College Scientists Foundation (to C.A.C.); and NIH grants R01 AI145687 (to P.A.) and UM1-AI144371 (to B.F.H.). Use of sector 22 (Southeast Region Collaborative Access team) at the Advanced Photon Source was supported

by the US Department of Energy, Basic Energy Sciences, Office of Science, under contract no. W-31-109-Eng-38. This work was performed in part at the cryo-EM facility in the Duke University Shared Materials Instrumentation Facility (SMIF), a member of the North Carolina Research Triangle Nanotechnology Network (RTNN), which is supported by the National Science Foundation (award ECCS-2025064) as part of the National Nanotechnology Coordinated Infrastructure (NNCI). This study utilized the computational resources offered by Duke Research Computing (https://rc.duke.edu/; NIH 1S10OD018164-01) at Duke University. We thank C. Kneifel, M. Newton, V. Orlikowski, and T. Milledge from the Duke Office of Information Technology and Research Computing for assisting with setting up and maintaining the computing environment.

AUTHOR CONTRIBUTIONS

C.A.C. determined cryo-EM structure of VRC40.01 in complex with BG505 SOSIP.v5.2 and performed bio-layer interferometry (BLI) binding assays; K.M. determined cryo-EM structures of VRC40.01, VRC33.01, and 179NC75 in complex with BG505 DS-SOSIP; R.K. isolated VRC33 and VRC40 antibodies, performed neutralization and ELISA studies, and provided protein VRC40.01 to C.A.C.; S.W. performed informatica analyses of structural and neutralization data and coordinated manuscript assembly; T.Z. determined crystal structures of VRC40.01 and VRC33.01 and provided proteins VRC40.01, VRC33.01, and 179NC75 to K.M. for cryo-EM; G.-Y.C. carried out neutralization fingerprint analysis; R.J.E., R.H., K.J., and M.K. assisted K.M. with cryo-EM structure determination; R.R. analyzed the 208-virus panel for glycan276 presence; B.C.L. and M.K.L. performed large-panel neutralization; A.S.O. assisted in protein production; C.H.-S. analyzed antibody genomics; J.D.T. assisted R.K. with VRC33 and VRC40 antibody isolation and analysis; J.L.T. and N.R.W. helped C.A.C. with cryo-EM sample preparation; B.Z. produced VRC33.01, VRC40.01, and 179NC75 for structural and neutralization analyses; N.A.D.-R. supervised neutralization assays; M.S.C. and B.F.H. provided CH314 and CH540 sera; L.S. supervised antibodyomics analyses; P.A. supervised cryo-EM structural analyses of BG505 DS-SOSIP complexes; A.B.W. supervised cryo-EM of VRC40.01-BG505-RM19R; J.R.M. supervised antibody isolation and neutralization analysis; P.D.K. oversaw the project and, with C.A.C. and S.W., provided a first draft of the manuscript, with all authors providing revisions and comments.

DECLARATION OF INTERESTS

The authors declare no competing interests.

Received: March 18, 2021
Revised: August 20, 2021
Accepted: October 12, 2021
Published: November 2, 2021

REFERENCES

Abbott, R.K., Lee, J.H., Menis, S., Skog, P., Rossi, M., Ota, T., Kulp, D.W., Bhullar, D., Kalyuzhniy, O., Havenar-Daughton, C., et al. (2018). Precursor Frequency and Affinity Determine B Cell Competitive Fitness in Germinal Centers, Tested with Germline-Targeting HIV Vaccine Immunogens. Immunity *48*, 133–146.e6.

Adams, P.D., Afonine, P.V., Bunkóczi, G., Chen, V.B., Davis, I.W., Echols, N., Headd, J.J., Hung, L.W., Kapral, G.J., Grosse-Kunstleve, R.W., et al. (2010). PHENIX: a comprehensive Python-based system for macromolecular structure solution. Acta Crystallogr. D Biol. Crystallogr. *66*, 213–221.

Agirre, J., Iglesias-Fernández, J., Rovira, C., Davies, G.J., Wilson, K.S., and Cowtan, K.D. (2015). Privateer: software for the conformational validation of carbohydrate structures. Nat. Struct. Mol. Biol. *22*, 833–834.

Balla-Jhagjhoorsingh, S.S., Corti, D., Heyndrickx, L., Willems, E., Vereecken, K., Davis, D., and Vanham, G. (2013). The N276 glycosylation site is required for HIV-1 neutralization by the CD4 binding site specific HJ16 monoclonal antibody. PLoS ONE *8*, e68863.

Barad, B.A., Echols, N., Wang, R.Y., Cheng, Y., DiMaio, F., Adams, P.D., and Fraser, J.S. (2015). EMRinger: side chain-directed model and map validation for 3D cryo-electron microscopy. Nat. Methods *12*, 943–946.

Bonsignori, M., Zhou, T., Sheng, Z., Chen, L., Gao, F., Joyce, M.G., Ozorowski, G., Chuang, G.Y., Schramm, C.A., Wiehe, K., et al.; NISC Comparative Sequencing Program (2016). Maturation Pathway from Germline to Broad HIV-1 Neutralizer of a CD4-Mimic Antibody. Cell *165*, 449–463.

Bonsignori, M., Scott, E., Wiehe, K., Easterhoff, D., Alam, S.M., Hwang, K.K., Cooper, M., Xia, S.M., Zhang, R., Montefiori, D.C., et al. (2018). Inference of the HIV-1 VRC01 Antibody Lineage Unmutated Common Ancestor Reveals Alternative Pathways to Overcome a Key Glycan Barrier. Immunity *49*, 1162–1174.e8.

Bräuninger, A., Goossens, T., Rajewsky, K., and Küppers, R. (2001). Regulation of immunoglobulin light chain gene rearrangements during early B cell development in the human. Eur. J. Immunol. *31*, 3631–3637.

Briney, B., Sok, D., Jardine, J.G., Kulp, D.W., Skog, P., Menis, S., Jacak, R., Kalyuzhniy, O., de Val, N., Sesterhenn, F., et al. (2016). Tailored Immunogens Direct Affinity Maturation toward HIV Neutralizing Antibodies. Cell *166*, 1459–1470.e11.

Casañal, A., Lohkamp, B., and Emsley, P. (2020). Current developments in Coot for macromolecular model building of Electron Cryo-microscopy and Crystallographic Data. Protein Sci. *29*, 1069–1078.

Chen, X., Zhou, T., Schmidt, S.D., Duan, H., Cheng, C., Chuang, G.Y., Gu, Y., Louder, M.K., Lin, B.C., Shen, C.H., et al. (2021). Vaccination induces maturation in a mouse model of diverse unmutated VRC01-class precursors to HIV-neutralizing antibodies with >50% breadth. Immunity *54*, 324–339.e8.

Chuang, G.Y., Acharya, P., Schmidt, S.D., Yang, Y., Louder, M.K., Zhou, T., Kwon, Y.D., Pancera, M., Bailer, R.T., Doria-Rose, N.A., et al. (2013). Residue-level prediction of HIV-1 antibody epitopes based on neutralization of diverse viral strains. J. Virol. *87*, 10047–10058.

Chuang, G.Y., Zhou, J., Acharya, P., Rawi, R., Shen, C.H., Sheng, Z., Zhang, B., Zhou, T., Bailer, R.T., Dandey, V.P., et al. (2019). Structural Survey of Broadly Neutralizing Antibodies Targeting the HIV-1 Env Trimer Delineates Epitope Categories and Characteristics of Recognition. Structure *27*, 196–206.e6.

Corti, D., Langedijk, J.P., Hinz, A., Seaman, M.S., Vanzetta, F., Fernandez-Rodriguez, B.M., Silacci, C., Pinna, D., Jarrossay, D., Balla-Jhagjhoorsingh, S., et al. (2010). Analysis of memory B cell responses and isolation of novel monoclonal antibodies with neutralizing breadth from HIV-1-infected individuals. PLoS ONE *5*, e8805.

Croll, T.I. (2018). ISOLDE: a physically realistic environment for model building into low-resolution electron-density maps. Acta Crystallogr. D Struct. Biol. *74*, 519–530.

Derking, R., Ozorowski, G., Sliepen, K., Yasmeen, A., Cupo, A., Torres, J.L., Julien, J.P., Lee, J.H., van Montfort, T., de Taeye, S.W., et al. (2015). Comprehensive antigenic map of a cleaved soluble HIV-1 envelope trimer. PLoS Pathog. *11*, e1004767.

Dey, B., Svehla, K., Xu, L., Wycuff, D., Zhou, T., Voss, G., Phogat, A., Chakrabarti, B.K., Li, Y., Shaw, G., et al. (2009). Structure-based stabilization of HIV-1 gp120 enhances humoral immune responses to the induced co-receptor binding site. PLoS Pathog. *5*, e1000445.

DiMaio, F., Tyka, M.D., Baker, M.L., Chiu, W., and Baker, D. (2009). Refinement of protein structures into low-resolution density maps using rosetta. J. Mol. Biol. *392*, 181–190.

Emsley, P., and Cowtan, K. (2004). Coot: model-building tools for molecular graphics. Acta Crystallogr. D Biol. Crystallogr. *60*, 2126–2132.

Freund, N.T., Horwitz, J.A., Nogueira, L., Sievers, S.A., Scharf, L., Scheid, J.F., Gazumyan, A., Liu, C., Velinzon, K., Goldenthal, A., et al. (2015). A New Glycan-Dependent CD4-Binding Site Neutralizing Antibody Exerts Pressure on HIV-1 In Vivo. PLoS Pathog. *11*, e1005238.

Georgiev, I.S., Doria-Rose, N.A., Zhou, T., Kwon, Y.D., Staupe, R.P., Moquin, S., Chuang, G.Y., Louder, M.K., Schmidt, S.D., Altae-Tran, H.R., et al. (2013).

Delineating antibody recognition in polyclonal sera from patterns of HIV-1 isolate neutralization. Science *340*, 751–756.

Go, E.P., Irungu, J., Zhang, Y., Dalpathado, D.S., Liao, H.X., Sutherland, L.L., Alam, S.M., Haynes, B.F., and Desaire, H. (2008). Glycosylation site-specific analysis of HIV envelope proteins (JR-FL and CON-S) reveals major differences in glycosylation site occupancy, glycoform profiles, and antigenic epitopes' accessibility. J. Proteome Res. *7*, 1660–1674.

Gristick, H.B., von Boehmer, L., West, A.P., Jr., Schamber, M., Gazumyan, A., Golijanin, J., Seaman, M.S., Fätkenheuer, G., Klein, F., Nussenzweig, M.C., and Bjorkman, P.J. (2016). Natively glycosylated HIV-1 Env structure reveals new mode for antibody recognition of the CD4-binding site. Nat. Struct. Mol. Biol. *23*, 906–915.

Gulla, K., Cibelli, N., Cooper, J.W., Fuller, H.C., Schneiderman, Z., Witter, S., Zhang, Y., Changela, A., Geng, H., Hatcher, C., et al.; Vrc Production Program (2021). A non-affinity purification process for GMP production of prefusion-closed HIV-1 envelope trimers from clades A and C for clinical evaluation. Vaccine *39*, 3379–3387.

Havenar-Daughton, C., Carnathan, D.G., Torrents de la Peña, A., Pauthner, M., Briney, B., Reiss, S.M., Wood, J.S., Kaushik, K., van Gils, M.J., Rosales, S.L., et al. (2016). Direct Probing of Germinal Center Responses Reveals Immunological Features and Bottlenecks for Neutralizing Antibody Responses to HIV Env Trimer. Cell Rep. *17*, 2195–2209.

Havenar-Daughton, C., Sarkar, A., Kulp, D.W., Toy, L., Hu, X., Deresa, I., Kalyuzhniy, O., Kaushik, K., Upadhyay, A.A., Menis, S., et al. (2018). The human naive B cell repertoire contains distinct subclasses for a germline-targeting HIV-1 vaccine immunogen. Sci. Transl. Med. *10*, eaat0381.

Helle, F., Duverlie, G., and Dubuisson, J. (2011). The hepatitis C virus glycan shield and evasion of the humoral immune response. Viruses *3*, 1909–1932.

Huang, J., Kang, B.H., Ishida, E., Zhou, T., Griesman, T., Sheng, Z., Wu, F., Doria-Rose, N.A., Zhang, B., McKee, K., et al. (2016). Identification of a CD4-Binding-Site Antibody to HIV that Evolved Near-Pan Neutralization Breadth. Immunity *45*, 1108–1121.

Jardine, J.G., Ota, T., Sok, D., Pauthner, M., Kulp, D.W., Kalyuzhniy, O., Skog, P.D., Thinnes, T.C., Bhullar, D., Briney, B., et al. (2015). HIV-1 VACCINES. Priming a broadly neutralizing antibody response to HIV-1 using a germline-targeting immunogen. Science *349*, 156–161.

Jardine, J.G., Kulp, D.W., Havenar-Daughton, C., Sarkar, A., Briney, B., Sok, D., Sesterhenn, F., Ereño-Orbea, J., Kalyuzhniy, O., Deresa, I., et al. (2016). HIV-1 broadly neutralizing antibody precursor B cells revealed by germline-targeting immunogen. Science *351*, 1458–1463.

Kabat, E.A. (1991). Sequences of Proteins of Immunological Interest: Tabulation and Analysis of Amino Acid and Nucleic Acid Sequences of Precursors, V-Regions, C-Regions, J-Chain, T-Cell Receptors for Antigen T-Cell Surface Antigens, [beta]2-Microglobulins, Major Histocompatibility Antigens, Thy-1, Complement, C-Reactive Protein, Thymopoietin, Integrins, Post-gamma Globulin, [alpha]2-Macroglobulins, and Other Related Proteins, Fifth Edition (US Department of Health and Human Services, Public Health Service, National Institutes of Health).

Kong, L., Ju, B., Chen, Y., He, L., Ren, L., Liu, J., Hong, K., Su, B., Wang, Z., Ozorowski, G., et al. (2016). Key gp120 Glycans Pose Roadblocks to the Rapid Development of VRC01-Class Antibodies in an HIV-1-Infected Chinese Donor. Immunity *44*, 939–950.

Krissinel, E., and Henrick, K. (2007). Inference of macromolecular assemblies from crystalline state. J. Mol. Biol. *372*, 774–797.

Kwon, Y.D., Pancera, M., Acharya, P., Georgiev, I.S., Crooks, E.T., Gorman, J., Joyce, M.G., Guttman, M., Ma, X., Narpala, S., et al. (2015). Crystal structure, conformational fixation and entry-related interactions of mature ligand-free HIV-1 Env. Nat. Struct. Mol. Biol. *22*, 522–531.

Kwon, Y.D., Chuang, G.Y., Zhang, B., Bailer, R.T., Doria-Rose, N.A., Gindin, T.S., Lin, B., Louder, M.K., McKee, K., O'Dell, S., et al. (2018). Surface-Matrix Screening Identifies Semi-specific Interactions that Improve Potency of a Near Pan-reactive HIV-1-Neutralizing Antibody. Cell Rep. *22*, 1798–1809.

Lennemann, N.J., Rhein, B.A., Ndungo, E., Chandran, K., Qiu, X., and Maury, W. (2014). Comprehensive functional analysis of N-linked glycans on Ebola virus GP1. MBio *5*, e00862-13.

Li, Y., Cleveland, B., Klots, I., Travis, B., Richardson, B.A., Anderson, D., Montefiori, D., Polacino, P., and Hu, S.L. (2008). Removal of a single N-linked glycan in human immunodeficiency virus type 1 gp120 results in an enhanced ability to induce neutralizing antibody responses. J. Virol. *82*, 638–651.

Liao, H.X., Lynch, R., Zhou, T., Gao, F., Alam, S.M., Boyd, S.D., Fire, A.Z., Roskin, K.M., Schramm, C.A., Zhang, Z., et al.; NISC Comparative Sequencing Program (2013). Co-evolution of a broadly neutralizing HIV-1 antibody and founder virus. Nature *496*, 469–476.

Lütteke, T., and von der Lieth, C.W. (2004). pdb-care (PDB carbohydrate residue check): a program to support annotation of complex carbohydrate structures in PDB files. BMC Bioinformatics *5*, 69.

Lyskov, S., Chou, F.C., Conchúir, S.O., Der, B.S., Drew, K., Kuroda, D., Xu, J., Weitzner, B.D., Renfrew, P.D., Sripakdeevong, P., et al. (2013). Serverification of molecular modeling applications: the Rosetta Online Server that Includes Everyone (ROSIE). PLoS ONE *8*, e63906.

Martin, J.T., Cottrell, C.A., Antanasijevic, A., Carnathan, D.G., Cossette, B.J., Enemuo, C.A., Gebru, E.H., Choe, Y., Viviano, F., Fischinger, S., et al. (2020). Targeting HIV Env immunogens to B cell follicles in nonhuman primates through immune complex or protein nanoparticle formulations. NPJ Vaccines *5*, 72.

McCoy, A.J., Grosse-Kunstleve, R.W., Adams, P.D., Winn, M.D., Storoni, L.C., and Read, R.J. (2007). Phaser crystallographic software. J. Appl. Cryst. *40*, 658–674.

McGuire, A.T., Hoot, S., Dreyer, A.M., Lippy, A., Stuart, A., Cohen, K.W., Jardine, J., Menis, S., Scheid, J.F., West, A.P., et al. (2013). Engineering HIV envelope protein to activate germline B cell receptors of broadly neutralizing anti-CD4 binding site antibodies. J. Exp. Med. *210*, 655–663.

Medina-Ramírez, M., Garces, F., Escolano, A., Skog, P., de Taeye, S.W., Del Moral-Sanchez, I., McGuire, A.T., Yasmeen, A., Behrens, A.J., Ozorowski, G., et al. (2017). Design and crystal structure of a native-like HIV-1 envelope trimer that engages multiple broadly neutralizing antibody precursors in vivo. J. Exp. Med. *214*, 2573–2590.

Otwinowski, Z., and Minor, W. (1997). Processing of X-ray diffraction data collected in oscillation mode. Methods Enzymol. *276*, 307–326.

Pancera, M., Zhou, T., Druz, A., Georgiev, I.S., Soto, C., Gorman, J., Huang, J., Acharya, P., Chuang, G.Y., Ofek, G., et al. (2014). Structure and immune recognition of trimeric pre-fusion HIV-1 Env. Nature *514*, 455–461.

Parks, K.R., MacCamy, A.J., Trichka, J., Gray, M., Weidle, C., Borst, A.J., Khechaduri, A., Takushi, B., Agrawal, P., Guenaga, J., et al. (2019). Overcoming Steric Restrictions of VRC01 HIV-1 Neutralizing Antibodies through Immunization. Cell Rep. *29*, 3060–3072.e7.

Pettersen, E.F., Goddard, T.D., Huang, C.C., Couch, G.S., Greenblatt, D.M., Meng, E.C., and Ferrin, T.E. (2004). UCSF Chimera—a visualization system for exploratory research and analysis. J. Comput. Chem. *25*, 1605–1612.

Pettersen, E.F., Goddard, T.D., Huang, C.C., Meng, E.C., Couch, G.S., Croll, T.I., Morris, J.H., and Ferrin, T.E. (2021). UCSF ChimeraX: Structure visualization for researchers, educators, and developers. Protein Sci. *30*, 70–82.

Punjani, A., Rubinstein, J.L., Fleet, D.J., and Brubaker, M.A. (2017). cryoSPARC: algorithms for rapid unsupervised cryo-EM structure determination. Nat. Methods *14*, 290–296.

Rawi, R., Rutten, L., Lai, Y.T., Olia, A.S., Blokland, S., Juraszek, J., Shen, C.H., Tsybovsky, Y., Verardi, R., Yang, Y., et al. (2020). Automated Design by Structure-Based Stabilization and Consensus Repair to Achieve Prefusion-Closed Envelope Trimers in a Wide Variety of HIV Strains. Cell Rep. *33*, 108432.

Sanders, R.W., Derking, R., Cupo, A., Julien, J.P., Yasmeen, A., de Val, N., Kim, H.J., Blattner, C., de la Peña, A.T., Korzun, J., et al. (2013). A next-generation cleaved, soluble HIV-1 Env trimer, BG505 SOSIP.664 gp140, expresses multiple epitopes for broadly neutralizing but not non-neutralizing antibodies. PLoS Pathog. *9*, e1003618.

Scharf, L., Scheid, J.F., Lee, J.H., West, A.P., Jr., Chen, C., Gao, H., Gnanapragasam, P.N., Mares, R., Seaman, M.S., Ward, A.B., et al. (2014). Antibody 8ANC195 reveals a site of broad vulnerability on the HIV-1 envelope spike. Cell Rep. *7*, 785–795.

Scheid, J.F., Mouquet, H., Ueberheide, B., Diskin, R., Klein, F., Oliveira, T.Y., Pietzsch, J., Fenyo, D., Abadir, A., Velinzon, K., et al. (2011). Sequence and structural convergence of broad and potent HIV antibodies that mimic CD4 binding. Science *333*, 1633–1637.

Schommers, P., Gruell, H., Abernathy, M.E., Tran, M.K., Dingens, A.S., Gristick, H.B., Barnes, C.O., Schoofs, T., Schlotz, M., Vanshylla, K., et al. (2020). Restriction of HIV-1 Escape by a Highly Broad and Potent Neutralizing Antibody. Cell *180*, 471–489.e22.

Seaman, M.S., Janes, H., Hawkins, N., Grandpre, L.E., Devoy, C., Giri, A., Coffey, R.T., Harris, L., Wood, B., Daniels, M.G., et al. (2010). Tiered categorization of a diverse panel of HIV-1 Env pseudoviruses for assessment of neutralizing antibodies. J. Virol. *84*, 1439–1452.

Sethna, Z., Elhanati, Y., Callan, C.G., Jr., Walczak, A.M., and Mora, T. (2019). OLGA: fast computation of generation probabilities of B- and T-cell receptor amino acid sequences and motifs. Bioinformatics *35*, 2974–2981.

Sommerstein, R., Flatz, L., Remy, M.M., Malinge, P., Magistrelli, G., Fischer, N., Sahin, M., Bergthaler, A., Igonet, S., Ter Meulen, J., et al. (2015). Arenavirus Glycan Shield Promotes Neutralizing Antibody Evasion and Protracted Infection. PLoS Pathog. *11*, e1005276.

Suloway, C., Pulokas, J., Fellmann, D., Cheng, A., Guerra, F., Quispe, J., Stagg, S., Potter, C.S., and Carragher, B. (2005). Automated molecular microscopy: the new Leginon system. J. Struct. Biol. *151*, 41–60.

Terwilliger, T.C., Ludtke, S.J., and Read, R.J. (2020). Improvement of cryo-EM maps by density modification. Nat Methods *17*, 923–927. https://doi.org/10.1038/s41592-020-0914-9.

Tian, M., Cheng, C., Chen, X., Duan, H., Cheng, H.L., Dao, M., Sheng, Z., Kimble, M., Wang, L., Lin, S., et al. (2016). Induction of HIV Neutralizing Antibody Lineages in Mice with Diverse Precursor Repertoires. Cell *166*, 1471–1484.e18.

Torrents de la Peña, A., Julien, J.P., de Taeye, S.W., Garces, F., Guttman, M., Ozorowski, G., Pritchard, L.K., Behrens, A.J., Go, E.P., Burger, J.A., et al. (2017). Improving the Immunogenicity of Native-like HIV-1 Envelope Trimers by Hyperstabilization. Cell Rep. *20*, 1805–1817.

Umotoy, J., Bagaya, B.S., Joyce, C., Schiffner, T., Menis, S., Saye-Francisco, K.L., Biddle, T., Mohan, S., Vollbrecht, T., Kalyuzhniy, O., et al.; IAVI Protocol C Investigators; IAVI African HIV Research Network (2019). Rapid and Focused Maturation of a VRC01-Class HIV Broadly Neutralizing Antibody Lineage Involves Both Binding and Accommodation of the N276-Glycan. Immunity *51*, 141–154.e6.

van Beusekom, B., Wezel, N., Hekkelman, M.L., Perrakis, A., Emsley, P., and Joosten, R.P. (2019). Building and rebuilding N-glycans in protein structure models. Acta Crystallogr. D Struct. Biol. *75*, 416–425.

Vu, H.L., Kwon, B., Yoon, K.J., Laegreid, W.W., Pattnaik, A.K., and Osorio, F.A. (2011). Immune evasion of porcine reproductive and respiratory syndrome virus through glycan shielding involves both glycoprotein 5 as well as glycoprotein 3. J. Virol. *85*, 5555–5564.

Weitzner, B.D., Jeliazkov, J.R., Lyskov, S., Marze, N., Kuroda, D., Frick, R., Adolf-Bryfogle, J., Biswas, N., Dunbrack, R.L., Jr., and Gray, J.J. (2017). Modeling and docking of antibody structures with Rosetta. Nat. Protoc. *12*, 401–416.

West, A.P., Jr., Diskin, R., Nussenzweig, M.C., and Bjorkman, P.J. (2012). Structural basis for germ-line gene usage of a potent class of antibodies targeting the CD4-binding site of HIV-1 gp120. Proc. Natl. Acad. Sci. USA *109*, E2083–E2090.

Wibmer, C.K., Gorman, J., Anthony, C.S., Mkhize, N.N., Druz, A., York, T., Schmidt, S.D., Labuschagne, P., Louder, M.K., Bailer, R.T., et al. (2016). Structure of an N276-Dependent HIV-1 Neutralizing Antibody Targeting a Rare V5 Glycan Hole Adjacent to the CD4 Binding Site. J. Virol. *90*, 10220–10235.

Williams, C.J., Headd, J.J., Moriarty, N.W., Prisant, M.G., Videau, L.L., Deis, L.N., Verma, V., Keedy, D.A., Hintze, B.J., Chen, V.B., et al. (2018). MolProbity: More and better reference data for improved all-atom structure validation. Protein Sci. *27*, 293–315.

Wu, X., Parast, A.B., Richardson, B.A., Nduati, R., John-Stewart, G., Mbori-Ngacha, D., Rainwater, S.M., and Overbaugh, J. (2006). Neutralization escape variants of human immunodeficiency virus type 1 are transmitted from mother to infant. J. Virol. *80*, 835–844.

Wu, X., Yang, Z.Y., Li, Y., Hogerkorp, C.M., Schief, W.R., Seaman, M.S., Zhou, T., Schmidt, S.D., Wu, L., Xu, L., et al. (2010). Rational design of envelope identifies broadly neutralizing human monoclonal antibodies to HIV-1. Science *329*, 856–861.

Wu, X., Zhou, T., Zhu, J., Zhang, B., Georgiev, I., Wang, C., Chen, X., Longo, N.S., Louder, M., McKee, K., et al.; NISC Comparative Sequencing Program (2011). Focused evolution of HIV-1 neutralizing antibodies revealed by structures and deep sequencing. Science *333*, 1593–1602.

Ye, J., Ma, N., Madden, T.L., and Ostell, J.M. (2013). IgBLAST: an immunoglobulin variable domain sequence analysis tool. Nucleic Acids Res. *41*, W34–W40.

Zhang, K. (2016). Gctf: Real-time CTF determination and correction. J. Struct. Biol. *193*, 1–12.

Zheng, S.Q., Palovcak, E., Armache, J.P., Verba, K.A., Cheng, Y., and Agard, D.A. (2017). MotionCor2: anisotropic correction of beam-induced motion for improved cryo-electron microscopy. Nat. Methods *14*, 331–332.

Zhou, T., Xu, L., Dey, B., Hessell, A.J., Van Ryk, D., Xiang, S.H., Yang, X., Zhang, M.Y., Zwick, M.B., Arthos, J., et al. (2007). Structural definition of a conserved neutralization epitope on HIV-1 gp120. Nature *445*, 732–737.

Zhou, T., Georgiev, I., Wu, X., Yang, Z.Y., Dai, K., Finzi, A., Kwon, Y.D., Scheid, J.F., Shi, W., Xu, L., et al. (2010). Structural basis for broad and potent neutralization of HIV-1 by antibody VRC01. Science *329*, 811–817.

Zhou, T., Zhu, J., Wu, X., Moquin, S., Zhang, B., Acharya, P., Georgiev, I.S., Altae-Tran, H.R., Chuang, G.Y., Joyce, M.G., et al.; NISC Comparative Sequencing Program (2013). Multidonor analysis reveals structural elements, genetic determinants, and maturation pathway for HIV-1 neutralization by VRC01-class antibodies. Immunity *39*, 245–258.

Zhou, T., Lynch, R.M., Chen, L., Acharya, P., Wu, X., Doria-Rose, N.A., Joyce, M.G., Lingwood, D., Soto, C., Bailer, R.T., et al.; NISC Comparative Sequencing Program (2015). Structural Repertoire of HIV-1-Neutralizing Antibodies Targeting the CD4 Supersite in 14 Donors. Cell *161*, 1280–1292.

Zhou, T., Doria-Rose, N.A., Cheng, C., Stewart-Jones, G.B.E., Chuang, G.Y., Chambers, M., Druz, A., Geng, H., McKee, K., Kwon, Y.D., et al. (2017). Quantification of the Impact of the HIV-1-Glycan Shield on Antibody Elicitation. Cell Rep. *19*, 719–732.

Zhou, T., Zheng, A., Baxa, U., Chuang, G.Y., Georgiev, I.S., Kong, R., O'Dell, S., Shahzad-Ul-Hussan, S., Shen, C.H., Tsybovsky, Y., et al.; NISC Comparative Sequencing Program (2018). A Neutralizing Antibody Recognizing Primarily N-Linked Glycan Targets the Silent Face of the HIV Envelope. Immunity *48*, 500–513.e6.

Zhou, T., Teng, I.T., Olia, A.S., Cerutti, G., Gorman, J., Nazzari, A., Shi, W., Tsybovsky, Y., Wang, L., Wang, S., et al. (2020). Structure-Based Design with Tag-Based Purification and In-Process Biotinylation Enable Streamlined Development of SARS-CoV-2 Spike Molecular Probes. Cell Rep. *33*, 108322.

Zhu, J., Wu, X., Zhang, B., McKee, K., O'Dell, S., Soto, C., Zhou, T., Casazza, J.P., Mullikin, J.C., Kwong, P.D., et al.; NISC Comparative Sequencing Program (2013). De novo identification of VRC01 class HIV-1-neutralizing antibodies by next-generation sequencing of B-cell transcripts. Proc. Natl. Acad. Sci. USA *110*, E4088–E4097.

STAR★METHODS

KEY RESOURCES TABLE

REAGENT or RESOURCE	SOURCE	IDENTIFIER
Antibodies		
1-18	(Schommers et al., 2020)	N/A
3BNC117	(Scheid et al., 2011)	N/A
8ANC195	(Scharf et al., 2014)	N/A
179NC75	(Freund et al., 2015)	N/A
CH103	(Liao et al., 2013)	N/A
HJ16	(Balla-Jhagjhoorsingh et al., 2013)	N/A
IOMA	(Gristick et al., 2016)	N/A
N6	(Huang et al., 2016)	N/A
RM19R	(Martin et al., 2020)	N/A
VRC01	(Wu et al., 2010)	RRID: AB_2491019
VRC13	(Zhou et al., 2015)	N/A
VRC16	(Zhou et al., 2015)	N/A
VRC33.01	This paper	N/A
VRC40.01	This paper	N/A
VRC40.02	This paper	N/A
VRC40.03	This paper	N/A
VRC40.04	This paper	N/A
Bacterial and virus strains		
208-strain panel for neutralization assessments	(Chuang et al., 2019)	N/A
Bal.01	NIH/VRC	N/A
BG505	(Wu et al., 2006)	N/A
BG505 T278A	This paper	N/A
Q168.a2	NIH/VRC	N/A
RWO20.2	NHI/VRC	N/A
TRJO.58	NIH/VRC	N/A
Chemicals, peptides, and recombinant proteins		
Superdex200 10/300GL Column	GE Healthcare Life Sciences	Cat# 28990944
BG505 DS-SOSIP	(Kwon et al., 2015)	N/A
BG505 SOSIPv5.2	(Havenar-Daughton et al., 2016)	N/A
BG505 T332N.SOSIP	(Derking et al., 2015)	N/A
Q23.17 RnS SOSIP	(Rawi et al., 2020)	N/A
HxB2 gp120 2CC and mutants	(Zhou et al., 2007)	N/A
Critical commercial assays		
Turbo293 Transfection Kit	ThermoFisher Scientific Inc.	Cat# A14525
BirA biotin-protein ligase bulk reaction kit	Avidity	BirA500
Deposited data		
Crystal structure VRC40.01 Fab	Protein Data Bank	PDB: 7L79
Crystal structure VRC33.01 Fab	Protein Data Bank	PDB: 7L77
Cryo-EM structure VRC40.01-BG505-RM19R	Protein Data Bank	PDB: 7LG6

(*Continued on next page*)

CellPress
OPEN ACCESS

Continued

REAGENT or RESOURCE	SOURCE	IDENTIFIER
Cryo-EM electron density map VRC40.01-BG505-RM19R	EMDB	EMDB: EMD-23312
Cryo-EM structure VRC40.01-BG505	Protein Data Bank	PDB: 7LL1
Cryo-EM electron density map VRC40.01-BG505	EMDB	EMDB: EMD-23411
Cryo-EM structure VRC33.01-BG505	Protein Data Bank	PDB: 7LL2
Cryo-EM electron density map VRC33.01-BG505	EMDB	EMDB: EMD-23412
Cryo-EM structure 179NC75-Q23.17	Protein Data Bank	PDB: 7LLK
Cryo-EM electron density map 179NC75-Q23.17	EMDB	EMDB: EMD-23424
Experimental models: Cell lines		
Expi293F cells	ThermoFisher Scientific Inc	Cat# A14527
FreeStyle 293-F cells	ThermoFisher Scientific Inc	Cat# R79007
HEK293F cells	ThermoFisher Scientific Inc	Cat# K1663
Experimental models: Organisms/strains		
None		
Recombinant DNA		
pVRC8400 vector	https://www.addgene.org	Cat# 63160
pVRC8400-VRC40.01 plasmid	This paper	N/A
pVRC8400-VRC40.02 plasmid	This paper	N/A
pVRC8400-VRC33.01 plasmid	This paper	N/A
pVRC8400-179NC75 plasmid	This paper	N/A
Software and algorithms		
Coot	(Emsley and Cowtan, 2004; Pettersen et al., 2004)	https://sbgrid.org/software/
cryoSPARC	(Punjani et al., 2017)	https://cryosparc.com
EMRinger	(Barad et al., 2015)	http://emringer.com/
EPU	ThermoFisher Scientific	https://www.thermofisher.com/us/en/home/electron-microscopy/products/software-em-3d-vis/epu-software.html
GCTF	(Zhang, 2016)	https://www2.mrc-lmb.cam.ac.uk/download/gctf/
GraphPad Prism Software	GraphPad Prism Software, Inc.	N/A
HKL2000	(Otwinowski and Minor, 1997)	https://hkl-xray.com
IgBlast	(Ye et al., 2013)	https://www.ncbi.nlm.nih.gov/igblast/
MolProbity	(Williams et al., 2018)	http://molprobity.biochem.duke.edu
OLGA	(Sethna et al., 2019)	https://github.com/statbiophys/OLGA
Pdb-care	(Lütteke and von der Lieth, 2004)	http://www.glycosciences.de/tools/pdb-care/
Phenix	(Adams et al., 2010)	https://sbgrid.org/software/
Phaser	(McCoy et al., 2007)	https://www.phaser.cimr.cam.ac.uk/index.php/Phaser_Crystallographic_Software
PISA	(Krissinel and Henrick, 2007)	https://www.ebi.ac.uk/pdbe/pisa/
Privateer	(Agirre et al., 2015)	https://smb.slac.stanford.edu/facilities/software/ccp4/html/privateer.html
The PyMOL Molecular Graphics System	Schrödinger, LLC	https://pymol.org/2/
Rosetta	(Lyskov et al., 2013; Weitzner et al., 2017)	https://rosie.graylab.jhu.edu
UCSF Chimera	(Pettersen et al., 2004)	https://www.cgl.ucsf.edu/chimera/

RESOURCE AVAILABILITY

Lead contact

Further information and requests for resources and reagents should be directed to and will be fulfilled by lead contact Peter D. Kwong (pdkwong@nih.gov).

Materials availability

Plasmids generated in this study are available upon request.

Data and code availability

- Cryo-EM maps have been deposited to the EMDB with accession codes EMD-23312, EMD-23411, EMD-23412, and EMD-23424, and fitted coordinates have been deposited to PDB with accession codes 7LG6, 7LL1, 7LL2, 7LLK. Crystal structures of VRC33.01 and VRC40.01 have been deposited to PDB with accession codes 7L77 and 7L99.
- This paper does not report original code.
- Any additional information required to reanalyze the data reported in this paper is available from the Lead Contact upon request.

EXPERIMENTAL MODEL AND SUBJECT DETAILS

Serum Samples

Serum samples were collected from HIV-infected donors CH540 and CH314 of the Center for HIV/AIDS Vaccine Immunology (CHAVI) cohort, after obtaining informed consent and appropriate Institutional Review Board approval.

Cell Lines

HEK293F, Expi293F and FreeStyle 293-F cells were purchased from Thermo Fisher Scientific. The cells were used directly from the commercial sources following manufacturer suggestions as described in detail below.

METHOD DETAILS

Fluorescence Activated Cell Sorting

The PBMCs from CHAVI donors CH540 and CH314 were sorted for IgG$^+$ B cells as described previously (Wu et al., 2010). Briefly, PBMCs from donor CHAVI CH540 (time point 1/24/08) and from donor CHAVI CH314 (time point 5/12/08) were used for antigen specific B cell sorting. CD3$^-$CD8$^-$CD14$^-$CD19$^+$IgG$^+$ memory B cells were stained with fluorescent conjugated probes 2CC-D368R, 2CC-D368R-N276D, and BG505 SOSIP.664. B cells that were 2CC-D368R positive and 2CC-D368R-N276D negative were sorted into 96-well plate at a single cell per well, while B cells that were BG505 SOSIP.664 positive were also sorted.

Single B Cell RT-PCR and Cloning

The variable regions of the heavy and light chains were recovered from single B cells deposited into 96-well microplate by reverse transcription followed by nested PCR as described previously (Wu et al., 2011). The PCR amplicons were sequenced and then cloned into the CMV/R IgG expression vectors. Fab expression vectors were made by introducing two stop codons following residue D234 (Kabat numbering) (Kabat, 1991) in the IgG heavy chain vectors using the QuikChange® Lightning Site-Directed Mutagenesis kit (Agilent). Sequences were verified by Sanger sequencing (Genewiz).

Env Protein Production and Purification

BG505 SOSIP.664 and BG505 SOSIP.v5.2 Env proteins were expressed in HEK293F cells and purified with either PGT145 or 2G12 affinity chromatography followed by size exclusion chromatography (SEC) using a HiLoad 16/600 Superdex pg200 (GE Healthcare) as described previously (Sanders et al., 2013; Torrents de la Peña et al., 2017). Q23.17 Env was stabilized in prefusion-closed conformation by structure-based stabilization and consensus repair (Q23.17 RnS-SOSIP) (Rawi et al., 2020) and expressed in 293Freestyle cells as a fusion protein with an N-terminal single chain Fc tag cleavable by HRV3C digestion (Zhou et al., 2020). The expressed fusion protein was secreted to the cell culture media was captured with Protein A resin. The tag was removed by on-column digestion with HRV3C protease, and the Env protein was eluted and then applied to a Superdex 200 gel filtration column equilibrated with PBS for final purification.

Antibody Preparation

DNA encoding antibody variable regions were synthesized and subcloned into the pVRC8400 vector, in which a HRV3C cleavage site was inserted in the heavy-chain hinge region. Plasmids of heavy and light chain pairs were co-transfected in Expi293F cells (Thermo

Fisher) using Turbo293 transfection reagent (Speed BioSystems) as described previously (Kwon et al., 2018). On day 6 post transfection, the culture supernatant was harvested and loaded on a protein A column. The column was washed with PBS, and IgG proteins were eluted with a low pH buffer. The eluted IgG proteins were cleaved by HRV3C, and the cleavage mixture was passed through a protein A column to separate the Fab fragments. The Fabs were further purified from a Superdex 200 column (GE) in a buffer containing 5 mM HEPES pH7.5 and 150 mM NaCl. Antibodies RM19R and VRC40.01 that were used for the cryoEM structure determination of the VRC40.01-RM19R-BG505 SOSIP.v5.2 complex were expressed in HEK293F cells. Briefly, HEK293F cells (Invitrogen) were co-transfected with heavy and light chain plasmids (1:1 ratio) using PEImax. Transfections were performed according to the manufacturer's protocol. Supernatants were harvested 4-6 days following transfection and passed through a 0.45 μm filter. Antibodies were purified using Protein A or MAbSelect (GE Healthcare) affinity chromatography. Fabs were purified using CaptureSelect CH1-XL (ThermoFisher) affinity chromatography.

Env-Pseudovirus Neutralization Assays

Serum and monoclonal antibody neutralization were assessed based on the single-round infection assay of TZM-bl cells with HIV-1 Env-pseudoviruses as described previously (Seaman et al., 2010). Serum samples were tested for neutralization against wild-type HIV-1 strains and their glycan-knockout mutants, as well as single alanine mutants.

Neutralization of monoclonal antibodies using a panel of 208 diverse Env-pseudoviruses representing the major subtypes and circulating recombinant forms was as previously described (Chuang et al., 2019). The data were calculated as half-maximum inhibitory concentration (IC_{50}) by comparison with control wells in the absence of antibodies.

Neutralization Depletion Assay

CH540 serum neutralization activity was depleted by pre-incubating with either the HxB2 gp120 core protein 2CC-D368R or the glycan276 knockout mutant protein 2CC-D368R/N276D. A media control was used as a mock depletion. Depleted serum was then tested for neutralization against a panel of 4 strains (Q168.a2, RWO20.2, Bal.01, TRJO.58) using the TZM-bl assay described above.

Neutralization Fingerprinting Analysis

Serum neutralization fingerprinting analysis was carried out using a panel of 21 diverse HIV-1 strains, as described previously (Georgiev et al., 2013), and the neutralization fingerprint of each serum was compared with those of the representative antibodies. Neutralization data of 208 HIV-1 viral strains were used in the fingerprint analysis for the glycan276- dependent antibodies in comparison with the representative categories of HIV-1 neutralizing antibodies. Antibodies with less than 10% breadth were excluded from this analysis.

Enzyme-Linked Immunosorbent Assay (ELISA)

ELISA was conducted as described previously (Zhou et al., 2018). HxB2 gp120 core protein (2CC) variants or YU2 gp120 protein were coated on the ELISA plate, and the binding of biotinylated antibodies was measured. For competition ELISA, antibodies were serially diluted and tested for competition with the biotinylated antibodies.

Bio-Layer Interferometry (BLI)

An Octet RED instrument (FortéBio) was used to assess antibody-antigen interactions by Biolayer Interferometry. VRC40.01 or VRC40.01_FR-H3 deletion Fabs were loaded onto anti-human Fab-CH1 (FAB2G) biosensors (FortéBio) at a concentration of 5 μg/mL in kinetics buffer (PBS, pH 7.4, 0.01% [w/v] BSA, and 0.002% [v/v] Tween 20) until a response of 0.5 nm shift was reached. Loaded biosensors were dipped into kinetics buffer for 1 min to acquire a baseline and then moved to wells containing 4 μM BG505 SOSIP.664 in kinetics buffer. The trimers were allowed to associate for 180 s before the biosensor were move back to the wells containing kinetics buffer where the baseline was acquired. Disassociation of the trimers from the Fab-loaded biosensors was recorded for 300 s. All BLI experiments were conducted at 37°C. Background subtracted results were plotted using Prism version 8.4.2 (GraphPad). For the VRC40.01 germline revertant binding assays, the biotinylated BG505 DS-SOSIP trimer (at 30 μg/ml) was captured on Streptavidin sensor tips and then dipped into the IgG antibody solution at a series of concentrations from 0.05 to 6.5 μM. The data were fitted with the Octet software Forte Data Analysis 11.

Crystallization and Structural Determination of Antibody Fab

Screening of initial crystallization conditions was carried out with 576 conditions using a Mosquito crystallization robot in vapor diffusion format with sitting drops containing 0.1 μl of protein and 0.1 μl of reservoir solution at 20 °C. Crystallizations hits were manually reproduced in hanging drops by mixing 0.5 μl protein with 0.5 μl reservoir solution. Crystals of VRC33.01 Fab were obtained in a condition containing 13% PEG3350, 10% Iso-propanol, 200 mM Ammonium Citrate, pH 4.5. Crystals were flash frozen in liquid nitrogen in the presence of 30% ethylene glycol before data collection. Crystals of VRC40.01 Fab were obtained in a condition containing 10.6% PEG 8000, 200 mM Zinc Acetate, Sodium Cacodylate, pH 6.5. 2R, 3R-butanediol at 15% was added as cryoprotectant to freeze crystals for data collection.

Diffraction data were collected at the SER-CAT beamline ID-22 (Advanced Photon Source, Argonne National Laboratory) with 1.0000 Å X-ray and processed with the HKL2000 suite (Otwinowski and Minor, 1997). Molecular replacement procedures were performed by using PHASER (McCoy et al., 2007) with PDB: 3SE8 as the initial model, and iterative model building and refinement were carried out in COOT (Emsley and Cowtan, 2004) and PHENIX (Adams et al., 2010), respectively. A cross validation (Rfree) test set consisting of 5% of the data was used throughout the refinement processes.

Cryo-EM Sample Preparation and Data Collection

For preparing the VRC40-BG505 SOSIP.v5.2 complex, 300 μg of BG505 SOSIPv5.2 was mixed with 1.2 mg VRC40.01 Fab and 1.2 mg RM19R Fab (added to improve particle tumbling) and incubated at RT overnight. The complex was SEC purified using a Hi-Load® 16/600 Superdex® pg200 (GE Healthcare) column in TBS. Fractions containing the complex were concentrated to 5 mg/mL using a 10 kDa Amicon® spin concentrator (Millipore). 3.5 μL of the complex was mixed with 0.57 μL of 0.04 mM lauryl maltose neopentyl glycol (LMNG) and applied to a C-Flat grid (CF-1.2/1.3-2C, Protochips, Inc.), which had been plasma-cleaned for 5 s using a mixture of N_2/O_2 (Gatan Solarus 950 Plasma system). The grid was blotted and plunged into liquid ethane using a Vitrobot Mark IV (ThermoFisher). The sample was imaged on an FEI Titan Krios electron microscope (ThermoFisher) operating at 300 keV equipped with Gatan K2 Summit direct electron director operating in counting mode. Automated data collection was performed using the Leginon software suite (Suloway et al., 2005). Micrograph movie frames were aligned and dose-weighted and CTF models were determined using cryoSPARCv2 (Punjani et al., 2017). Particle picking, 2D classification, Ab-initio reconstruction, and 3D refinement were conducted using cryoSPARCv2. Data collection and processing parameters are reported in Table S3.

For preparing the VRC40.01-BG505 DS-SOSIP, VRC33.01-BG505BG505 DS-SOSIP and 179NC75-Q23.17_RnS-SOSIP complexes, Env at a final concentration of 2-3 mg/mL, was incubated with 5-6-fold molar excess of the antibody Fab fragments. A Quantifoil-1.2/1.3 holey carbon grid was glow discharged for 15 s in a PELCO easiGlow Glow Discharge Cleaning System before depositing 2.5 μL of protein complex. Following a 30 s incubation in > 95% humid chamber and blotting away excess protein for 2.5 s on a blotting paper, the grid was plunge frozen into liquid ethane using a Leica EM GP2 plunge freezer (Leica Microsystems). Cryo-EM data were collected on a Titan Krios equipped with a K3 detector (Gatan) operating in counting mode. Data were acquired using the Latitude system (Gatan). The dose was fractionated over 60 raw frames; individual frames were aligned and dose-weighted (Zheng et al., 2017). CTF estimation, particle picking, 2D classifications, *ab initio* model generation, heterogeneous refinements, homogeneous 3D refinements and local resolution calculations were carried out in cryoSPARC. Data collection and processing parameters are reported in Table S3.

Atomic Model Building and Refinement

For determination of the VRC40.01-BG505 SOSIP.v5.2 complex structure, a homology model for the Fv region of VRC40.01 was generated using the Rosetta antibody protocol available on the ROSIE server (Lyskov et al., 2013; Weitzner et al., 2017). An initial molecular model of the BG505 SOSIP.v5.2 with VRC40.01 and RM19R bound was built by docking the PDB: 6VKN (Martin et al., 2020) and the VRC40.01 homology model into the EM density map using UCSF Chimera (Pettersen et al., 2004). The VRC40.01 Fv was manually rebuilt using Coot (Casañal et al., 2020). The model was iteratively refined into the EM density map using RosettaRelax and Coot (Casañal et al., 2020; DiMaio et al., 2009; van Beusekom et al., 2019). Glycan structures were validated using Privateer and pdb-care (Agirre et al., 2015; Lütteke and von der Lieth, 2004). Overall structures were evaluated using EMRinger and MolProbity (Barad et al., 2015; Williams et al., 2018). Protein interface calculations were performed using Protein Interfaces, Surfaces, and Assemblies (PISA) service of the European Bioinformatics Institute (EBI) (Krissinel and Henrick, 2007). Final model statistics are summarized in Table S3.

An initial molecular model of the BG505 DS-SOSIP with VRC40.01 bound was built by docking the PDB: 6VKN (Martin et al., 2020) and the high-resolution VRC40.01 crystal structure into the EM density map using UCSF Chimera (Pettersen et al., 2004). Similarly, an initial molecular model of BG505 DS-SOSIP with VRC33 was built by docking Env and high-resolution VRC33.01 crystal structures into the EM density map. The 179NC75 complex cryo-EM map was improved from their half maps using density modification program ResolveCryoEM (Terwilliger et al., 2020). A homology model for the Fv region of 179NC75 was generated using the Rosetta antibody protocol available on the ROSIE server (Lyskov et al., 2013; Weitzner et al., 2017). This model was used with Q23.17_DS-SOSIP Env to build the initial model for 179NC75 complex. The Fv regions of 179NC75 were manually rebuilt using Coot (Casañal et al., 2020). The models were iteratively refined into the EM density map using RosettaRelax, ISOLDE and Coot (Casañal et al., 2020; Croll, 2018; DiMaio et al., 2009; van Beusekom et al., 2019). Overall structures were evaluated using EMRinger and MolProbity (Barad et al., 2015; Williams et al., 2018). Figures were generated using PyMOL (Schrodinger; https://pymol.org/2/) and UCSF ChimeraX (Pettersen et al., 2021). Final model statistics are summarized in Table S3.

Analysis of Antibody Approach Vectors and Angles

To analyze the binding interactions between CD4bs antibodies and Env trimer, we superimposed one gp120 subunit of the structures with that of the VRC40.01-Env complex to have a common reference frame for comparison. We defined the antibody approach vector as a line from the centroid of the antibody epitope, calculated using PyMOL as Env atoms within 5.5 Å of the antibody excluding the glycan atoms, to the center of the variable domain of antibody Fab, defined as the center between the two Cα-atoms of the conserved cystine in the variable domain. To quantify the antibody approach angles, we calculated two angles for each

approach vector: the latitudinal angle, which explored the freedom perpendicular to the viral membrane along the trimer axis, and the longitudinal angle, which explored the freedom parallel to the viral membrane between protomers. The latitudinal angle was calculated as the angle between the antibody approach vector and the trimer three-fold axis, and the longitudinal angle was calculated as the angle between the antibody approach vector and the protomer axis, which is defined as a line passing through the centroid of CD4bs, perpendicular and intercepting the trimer axis.

Analysis of the HIV-1 Env Trimer-Antibody Interface

The buried surface area of the Env trimer in complex with antibodies was determined using PISA (Krissinel and Henrick, 2007) from the cryo-EM structures. The buried surface areas between the antibody heavy and light chains and the Env protein surface or the glycan276 were calculated separately from the PISA output.

Sequence Signature and Recombination Frequency

Antibody sequence signatures were determined based on the antibody-Env complex structures as described previously (Zhu et al., 2013). The buried surface area at the antibody-Env interface was analyzed with PISA, and residues on CDR 3 with a buried surface area larger than 20 $Å^2$ were selected as the sequence signature. The recombination frequency of an antibody was calculated as the product of the probability of heavy-chain recombination frequency, the probability of using kappa or lambda chain, and the probability of light-chain recombination frequency. The germline genes of heavy and light chains were assigned by IgBlast (Ye et al., 2013). The probability of having specific V-D-J recombination was computed using OLGA (Sethna et al., 2019), with heavy and light chain recombination models of 3 healthy donors constructed from next-generation sequencing samples (BioProject: PRJNA511481). The percentage of using kappa and light chain (60:40) (Bräuninger et al., 2001) was used for the probability of using kappa or lambda chain.

QUANTIFICATION AND STATISTICAL ANALYSIS

Cryo-EM data were processed and analyzed using CryoSparc and Relion. Cryo-EM structural statistics were analyzed with Phenix and Molprobity. Statistical details of experiments are described in Method Details or figure legends.

Cell Reports, Volume *37*

Supplemental information

Structural basis of glycan276-dependent recognition by HIV-1 broadly neutralizing antibodies

Christopher A. Cottrell, Kartik Manne, Rui Kong, Shuishu Wang, Tongqing Zhou, Gwo-Yu Chuang, Robert J. Edwards, Rory Henderson, Katarzyna Janowska, Megan Kopp, Bob C. Lin, Mark K. Louder, Adam S. Olia, Reda Rawi, Chen-Hsiang Shen, Justin D. Taft, Jonathan L. Torres, Nelson R. Wu, Baoshan Zhang, Nicole A. Doria-Rose, Myron S. Cohen, Barton F. Haynes, Lawrence Shapiro, Andrew B. Ward, Priyamvada Acharya, John R. Mascola, and Peter D. Kwong

Cell Reports, Volume *37*

Supplemental information

Structural basis of glycan276-dependent recognition by HIV-1 broadly neutralizing antibodies

Christopher A. Cottrell, Kartik Manne, Rui Kong, Shuishu Wang, Tongqing Zhou, Gwo-Yu Chuang, Robert J. Edwards, Rory Henderson, Katarzyna Janowska, Megan Kopp, Bob C. Lin, Mark K. Louder, Adam S. Olia, Reda Rawi, Chen-Hsiang Shen, Justin D. Taft, Jonathan L. Torres, Nelson R. Wu, Baoshan Zhang, Nicole A. Doria-Rose, Myron S. Cohen, Barton F. Haynes, Lawrence Shapiro, Andrew B. Ward, Priyamvada Acharya, John R. Mascola, and Peter D. Kwong

Table S1. Heavy and light chain DNA sequences of CH540-VRC33 and CH314-VRC40 antibodies, related to Figure 1. For heavy chain, the sequence of VDJ region was shown. For light chain, the sequence of VJ region was shown.

Antibody	Heavy chain	Light chain
VRC33.01	CAGGTGCAGCTACAACAGTGGGGCGCAGGACTGTTGAAGCC<br>TTCGGAGACCCTGTCCCTCACGTGCGCTCTTTATGGTCGGT<br>CCCTCAATGGCAACTATTGGAGTTGGATCCGCCAGTCCCCA<br>GGGAAGGGGCTGGAGTGGATTGGGGAGATCAATCATAGTGG<br>AAGCACCTACTTCAACCCGTCCTTCAAGAGTCGCGTCGCCA<br>TGTCAGTGGACACGTCTAAGAGTCAATTCTCCCTGAAGCTG<br>AATTCTGTGACCGCCGCGGACACGGGTATTTATTTCTGTGC<br>GCGAGGCAAAAGATACAGCGCCAGCTACTCTAATTACTTTG<br>GCGTCTGGGGCCAGGGAACCCAGGTCACCGTCTCCTCAG	GACATCCAGATGACCCAGTCTCCTTCCACCCTGT<br>CTGCATCTGTAGGAGACAGAGTCGACATCACTTG<br>CCGGGCCAGTCAGAGTATTAGTCGCTGGTTGGCC<br>TGGTATCAGCAGAAGCCAGGAAAAGCCCCTAAAG<br>TCCTCATCTATGAGGCGTCTCTTTTAGCAAATGG<br>AGTCCCATCCAGGTTCAGCGGACACTTCAACGGC<br>AGAGAGTCTGCGACAGACTTCACACTCACCATCA<br>GCAGCCTGCAGCCTGACGATGTTGCAACTTATTA<br>CTGCCAACATTATATGGCTGATCCCCGTTTTGGC<br>CAGGGGACCAAGCTGGAGATCAAAC
VRC40.01	CAGGTGCAGTTGATACAGTCCGGGCCTCAGTTTAAGACGCC<br>TGGGGCCTCAGTGACGGTCTCGTGCAAGGCCTCAGGATACA<br>TCTTCACCGACTACCTCATACACTGGGTGCGGCTGGTCCCC<br>GGAAAGGGCCTTGAGTGGCTGGGCCGAATCAACACCAATGC<br>TGGTCTTATGTATCTTTCACATAAATTTGAGGGTCGCCTCA<br>TTCTGAGGAGAGTAGTTGACTGGAGGACACCGTCCCTGGGT<br>ACAGTCAACATGGAATTAAGGAACGTCAGATCTGACGACTC<br>GGCCATATATTTTTGTGGGCGGGTCGTCGACGGCTTTAACG<br>CGGCGGGCCCCCTTGAGTTTTGGGGCCAGGGAAGTCCAGTC<br>ATCGTTTCTTCA	CAAGTGGTGATGACGCAGTCTCCAGCCACCCTGT<br>CTCTGTCTCCGGGGGAGACGGCCGCCGTCTCCTG<br>CAGGGCCAGCCAATATGTTGACCGCTCTATATCT<br>TGGTATCAACTAAAAACTGGCCGGGCTCCCAGAC<br>TCCTCGTCTATGCTGCATCGTCCAGGTCCATTGG<br>TGTCCCAGACAGGTTCAGTGGCAGTGGGTCTGGG<br>AGAGACTTCACTCTCACCATCAGAGGCGTCCAGT<br>CTGACGACTTTGCACTTTATTACTGTCAACAAGA<br>TTACTACTGGCCGGTCACCTTCGGCCAAGGGACA<br>CGCCTGGACATGAAA
VRC40.02	CAGGTGCGATTGATGCAATCCGGGCCTCAACTTAAGACGCC<br>TGGGGCCTCAGTGACGGTCTCGTGCAAGGCCTCAGGATACA<br>TCTTCACCGACTACCTCATACACTGGGTGCGGCTAGTCCCC<br>GGAAAGGGCCTTGAGTGGCTGGGCCGAATCAACACCAATGG<br>TGGTCTTATGTACCTTTCATATAAATTTGAGGGTCGCCTCA<br>TTCTGAGGAGAGACGTTGACTGGAGGACACCGTCCCTGGGC<br>ACAGTCTACATGGAATTAAAGAACCTCAGATCTGACGACTC<br>GGCCATATATTTTTGTGGGCGAGTCGTCGACGGCTTTAACG<br>CGGCGGGCCCCCTTGAGTTTTGGGGCCAGGGAAGTCCAGTC<br>ATCGTTTCTTCA	CAAGTGGTGATGACGCAGTCTCCAGTCACCCTGT<br>CTGTGTCTCCGGGGGAGACGGCCGCCGTCTCCTG<br>CAGGGCCAGCCAATATGTTGACCGCTCTATATCT<br>TGGTATCAACTAAAAACTGGCCGGGCTCCCAGAC<br>TCCTCGTCTATGCTGCATCGTCCAGGTCCATTGG<br>TGTCCCAGACAGGTTCAGTGGCAGTGGGTCTGGG<br>AGAGACTTCACTCTCACCATCAGAGGCGTCCAGT<br>CTGACGACTTTGCAGTTTATTACTGTCAACAAGA<br>TTACTACTGGCCGGTCACCTTCGGCCAAGGGACA<br>CGCCTGGACATGAAA
VRC40.03	CAGGTGCAGTTGATACAGTCTGGGCCTCAACTTAAGACGCC<br>TGGGGCCTCAGTGACTGTCTCGTGCAAGGCCTCAGGATACG<br>TGTTCGCCGACTACCTCATACACTGGGTGCGACTGGTCCCC<br>GGAAAGGGCCTTGAGTGGCTGGGCCGAATCAACACCAATGC<br>TGGTCTTATGTACCTTTCACATAAATTTGAAGGTCGCCTCA<br>TTTTGAGGAGAGACCGTGACTGGAGGACACCGTCCCTGGGC<br>ACACTCTACATGGAATTAAGGAACCTAAAATCTGACGATTC<br>GGCCATATATTTTTGTGGGCGGGTCGTCGACGGCTTTAACG<br>CGGCGGGCCCCCTTGAGTTTTGGGGCCAGGGAAGTCCGGTC<br>ATCGTTTCTTCAG	CAAGTGTTGATGACGCAGTCTCCAGCCACCCTGT<br>CTGTGTCTCCGGGGGAGACGGCCGCCGTCTCCTG<br>CAGGGCCAGCCAGTATGTTGACCGCTCTATATCT<br>TGGTATCAGGTAAAAAGTGGCCGGGCTCCCAGAC<br>TCCTCGTCTATGCTGCATCGTCCAGGTCCATTGG<br>TGTCCCGGACAGGTTCAGTGGCAGTGGGTCTGGG<br>ACAGACTTCACTCTCACCATCAGAGGCGTCCAGT<br>CTGACGACTTTGCACTTTATTACTGTCAACAAGA<br>TTACGGCTGGCCGGTCACCTTCGGCCAAGGGACA<br>CGCCTGGACATGAAAG
VRC40.04	CAAGTGCGGTTGATGCAGTCTGGGACTGAATTTAAGACGCC<br>TGGGGCCTCAGTGAAGGTCTCGTGCAAGACCTCAGGATACA<br>TCTTCAGCGACTACTTAATACACTGGGTGCGACTAGTCCCC<br>GGAAAGGGCCTTGAGTGGCTGGGGCGGATCAACACTAACGC<br>TGGTCTTATGTACCTTTCACCGAGATTTGAGGGTCGCGTCA<br>TTCTGAGGAGAGAGAGTTCCTTCAGGACACCATCCCTGGGC<br>ACAGTCTACATGGAATTAAGGAACCTAAAATTTGACGACTC<br>GGCCGTCTACTTTTGTGGACGAGTCGTCGACGGATTTAACG<br>CGGCGGGCCCCCTTGAATTTTGGGGCCAGGGGAGCCTGGTC<br>ATCGTCTCCTCCG	GAAGTGGTGATGACGCAGTCTCCAGCCACCCTGT<br>CTGTGTCTCCGGGGGAAGAAGCCGCCCTCTCCTG<br>TGGGGCCAGCGACTATATTGACCGGTCTGTGTCC<br>TGGTATCAACTAAAACCTGGCCGGGCTCCCAGAC<br>TCCTCGTCTATGCTGCGTCCTCCAGGTCCATTGG<br>TATCCCAGACAGGTTCAGTGGCAGTGGGTCTGGG<br>ACAGCCTTCACTCTCACCATCAGAGGCGTCCAGT<br>CTGACGACTTTGCCCTCTATTACTGTCAACAAGA<br>CAAATACTGGCCGGTCACCTTCGGCCAAGGGACA<br>CGCCTGGACATGAAAC

Table S3. Cryo-EM data collection, refinement and validation statistics, related to Figures 2, 3 and 5.

	VRC40.01 and RM19R with BG505 SOSIPv5.2	VRC40.01 with BG505 DS-SOSIP	VRC33.01 with BG505 DS-SOSIP	179NC75 with Q23.17 RnS-SOSIP
Access codes	PDB: 7LG6<br>EMD-23312	PDB: 7LL1<br>EMD-23411	PDB: 7LL2<br>EMD-23412	PDB: 7LLK<br>EMD-23424
**Data collection and processing**				
Magnification	29,000	92,000	92,000	92,000
Voltage (kV)	300	300	300	300
Electron exposure (e^{-}/Å^2)	51.5	58.5	58.5	45
Defocus range (μm)	-0.8 to -3.6	-0.75 to -2.5	-0.75 to -2.5	-0.75 to -2.5
Pixel size (Å)	1.03	1.08	1.08	1.00
Symmetry imposed	C3	C3	C3	C3
Final particle number	32,186	327,299	220,262	41,476
Map resolution (Å)	3.28	3.73	3.73	4.8
FSC threshold	0.143	0.143	0.143	0.143
**Refinement**				
Map sharpening B factor (Å^2)	-81.8	-74.1	-61.9	-77.8
Model composition				
Non-hydrogen atoms	26565	25320	24852	20163
Protein residues	3153	3135	3060	2400
Ligands BMA	9	9	6	21
FUC	3	0	0	0
MAN	24	12	15	18
NAG	99	51	78	66
B factors (Å^2) (min/max/mean)				
Protein	0.0/321.3/44.5	1.0/255.1/13.8	0.5/204.4/4.4	0.0/113.4/9.4
Ligand	21.3/119.8/66.6	30.0/30.0/30.0	30.0/30.0/30.0	30.0/30.0/30.0
R.m.s. deviations				
Bond lengths (Å)	0.022	0.013	0.013	0.003
Bond angles (°)	1.75	1.90	1.79	0.713
Validation				
MolProbity score	1.00	1.25	1.07	2.13
Clash score	0.61	0.53	0.38	1.76
Rotamer outliers (%)	0.33	1.43	1.14	5.09
Ramachandran plot				
Favored (%)	95.75	93.02	93.43	85.51
Allowed (%)	3.57	6.62	6.11	11.54
Disallowed (%)	0.68	0.36	0.46	2.95
EMRinger score	3.99	2.56	2.70	1.15

Table S4. Crystallographic data and refinement statistics, related to Figures 2 and 3.

**Protein complex**	VRC33.01 Fab	VRC40.01 Fab
**PDB accession code**	7L77	7L79
**Data collection**		
Growth condition		
Space group	P 1 2_1 1	P2_1 2_1 2_1
Cell constants		
a, b, c (Å)	49.2, 70.8, 65.4	69.8, 136.0, 136.9
α, β, γ (°)	90.0, 99.8, 90.0	90, 90, 90
Unique reflections	62495 (5581)	27995 (2392)
Wavelength (Å)	1.0000	1.0000
Resolution (Å)	28.59 - 1.54 (1.60 - 1.54)	35.63 - 2.83 (2.93 - 2.83)
R_{sym}	7.9 (52.0)	20.6 (79.6)
R_{pim}	5.0 (36.0)	12.4 (50.8)
$CC_{1/2}$	0.966 (0.674)	0.970 (0.895)
I / σI	27.8 (2.5)	10.3 (1.5)
Completeness (%)	96.2 (86.0)	97.0 (84.7)
Redundancy	3.5 (2.8)	3.7 (3.3)
Wilson B-Factor (Å^2)	17	55
**Refinement**		
Resolution (Å)	29.21 - 1.54 (1.58 -1.54)	36.92 - 2.83 (2.93-2.83)
Reflections used in refinement	62487 (5579)	27954 (2388)
R_{work} / R_{free} (%)	15.2 (23.2) / 18.7 (25.4)	22.0 (32.7) / 28.0 (37.8)
No. atoms		
Protein	3380	6776
Ligand/ion	0	9
Water	516	100
B-factors (Å^2)		
Protein	24	50
Ligand/ion	-	62
Water	37.6	42
R.m.s. deviations		
Bond lengths (Å)	0.010	0.007
Bond angles (°)	1.19	0.90
Ramachandran		
Favored regions (%)	97.94	92.37
Allowed regions (%)	2.06	6.95
Disallowed regions (%)	0.00	0.68

Values in parentheses are for the highest-resolution shells.
$R_{sym} = \Sigma|I - \langle I\rangle|/\Sigma\langle I\rangle$, where I is the observed intensity, and <I> is the average intensity of multiple observations of symmetry-related reflections.
$R = \Sigma_{hkl}||F_{obs}| - |F_{calc}||/\Sigma_{hkl}|F_{obs}|$.
R_{free} is calculated from 5% of the reflections excluded from refinement.

A

Serum	Date	VRC01-like	b12-like	CD4-like	HJ16-like	8ANC195-like	PG9-like	PGT128-like	2G12-like	2F5-like	10E8-like	Target function
CH314	5/12/08	0.00	0.00	0.00	0.46	0.31	0.00	0.00	0.23	0.00	0.00	24.8
CH540	1/24/08	0.23	0.21	0.00	0.36	0.20	0.00	0.00	0.00	0.00	0.00	21.1

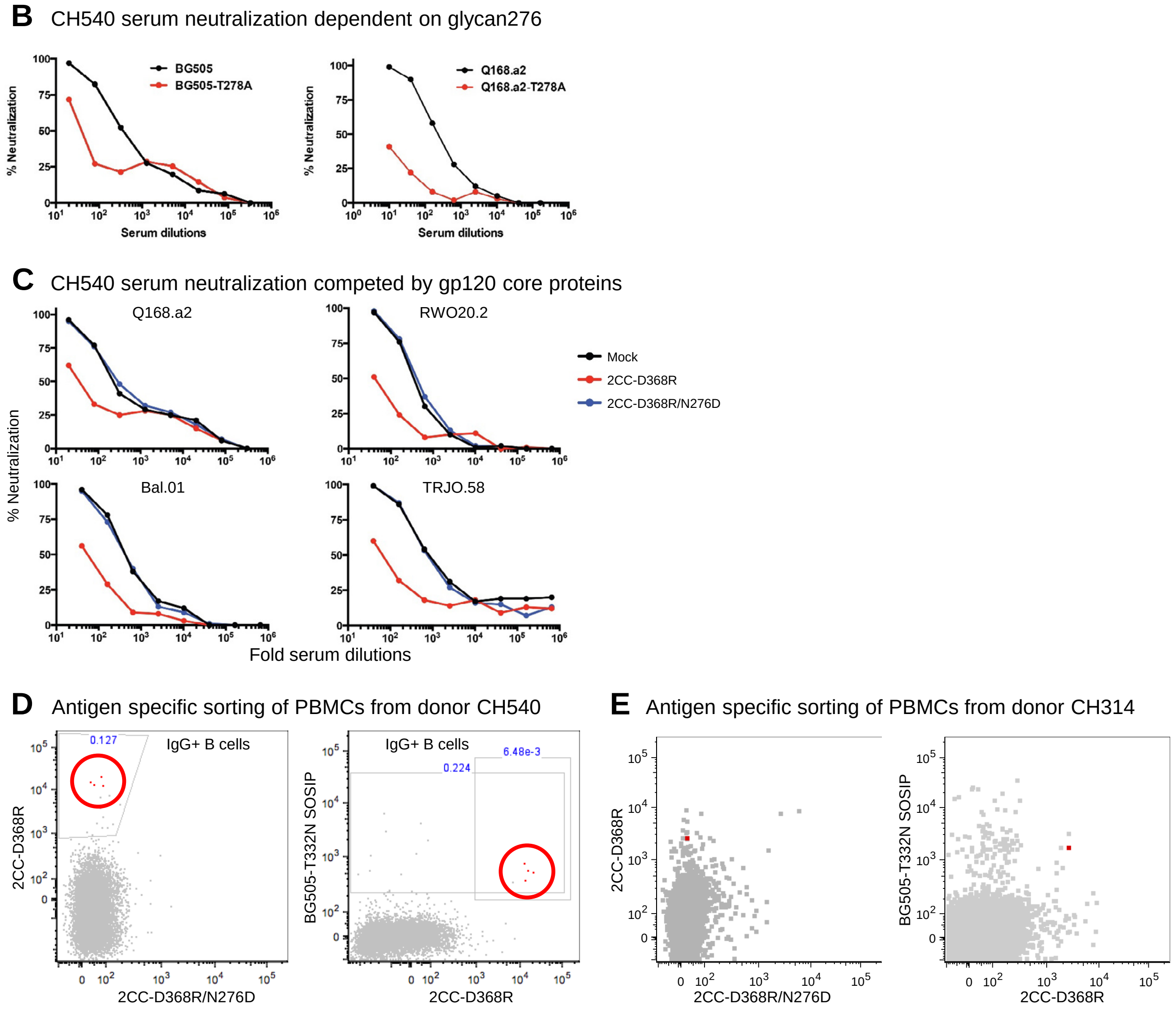

Figure S1. Serum neutralization analyses indicate Donor CH540 and CH314 sera to contain CD4bs-targeting antibodies that are glycan276 dependent, related to Figure 1.

(**A**) Neutralization fingerprint analysis of serum samples from donors CH540 indicated presence of neutralizing antibodies similar to VRC01 and b12, as well as glycan276-dependent antibodies HJ16 and 8ANC195. (**B**) CH540 serum neutralization of HIV-1 strains is dependent on the presence of glycan276. (**C**) CH540 serum neutralization of strains Q168.a2, RWO20.2, Bal.01, and TRJO.58 was competed by gp120 core protein 2CC-D368R but not by the glycan276-knockout mutant 2CC-D368R/N276D. (**D**) Antigen-specific B cell sorting identified four PBMCs from donor CH540 that bound to gp120 core only when glycan276 was present. $CD3^-CD8^-CD14^-CD19^+IgG^+$ memory B cells were stained with probes 2CC-D368R, 2CC-D368R/N276D, and gp140 trimer BG505 T332N.SOSIP. (**E**) Antigen-specific B cell sorting identified one PBMC from donor CH314 that bound to gp120 core only when glycan276 was present.

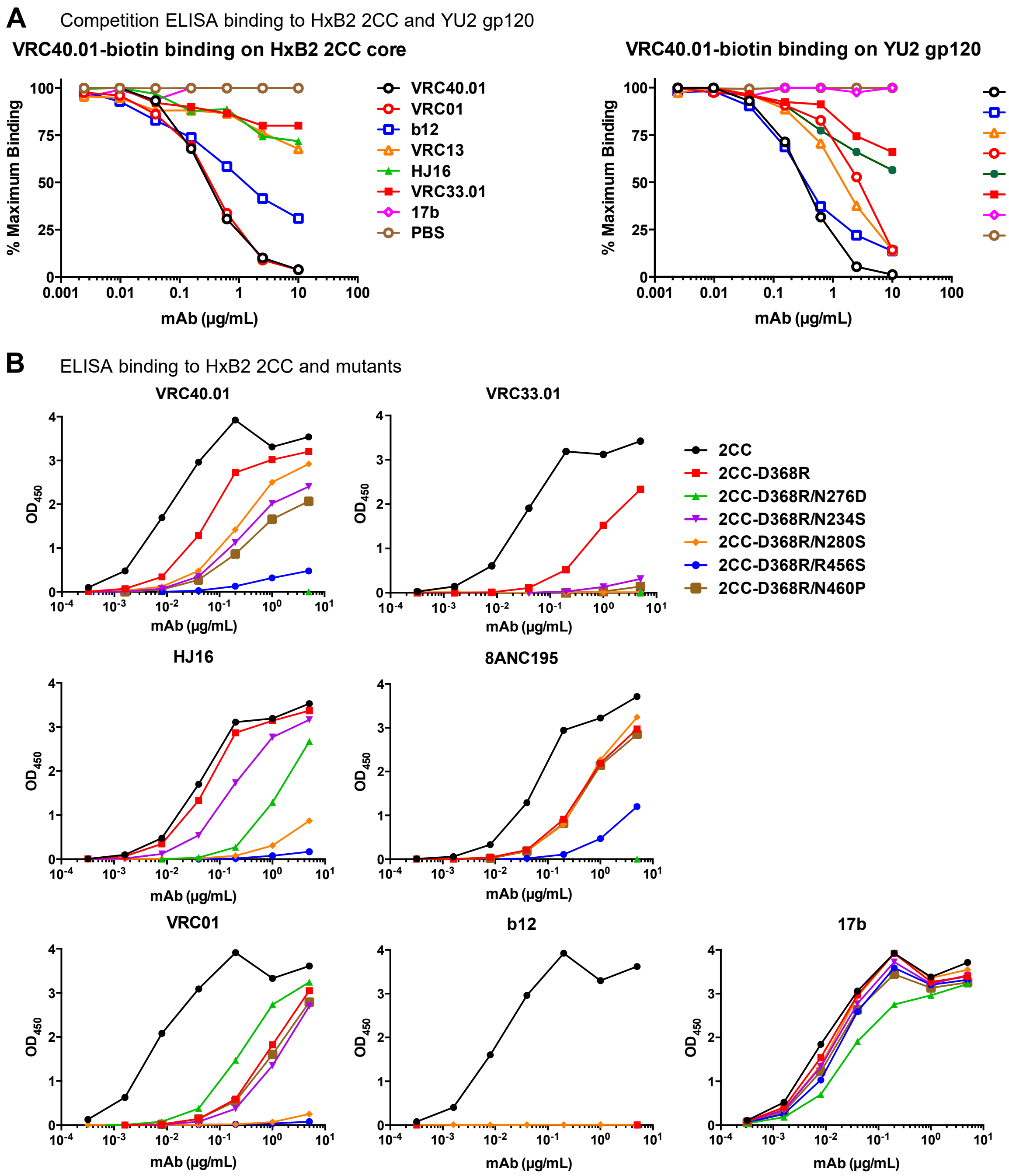

Figure S2. Binding analyses indicate CH540-VRC40.01 and CH314-VRC33.01 to target the CD4bs and glycan276, related to Figure 1.

(**A**) Competition ELISA for binding of biotinylated CH540-VRC40.01 to HxB2 2CC core (left) or YU2 gp120 (right) by CH314-VRC33.01 and various CD4bs antibodies. Percent maximum binding is plotted against competing antibody concentration. (**B**) ELISA binding of VRC40.01and VRC33.01 to HxB2 gp120 2CC core and its various mutants. Parallel assays were performed for glycan276-dependent antibodies HJ16 and 8ANC195, CD4bs Abs VRC01 and b12, and CD4i antibody 17b for comparison.

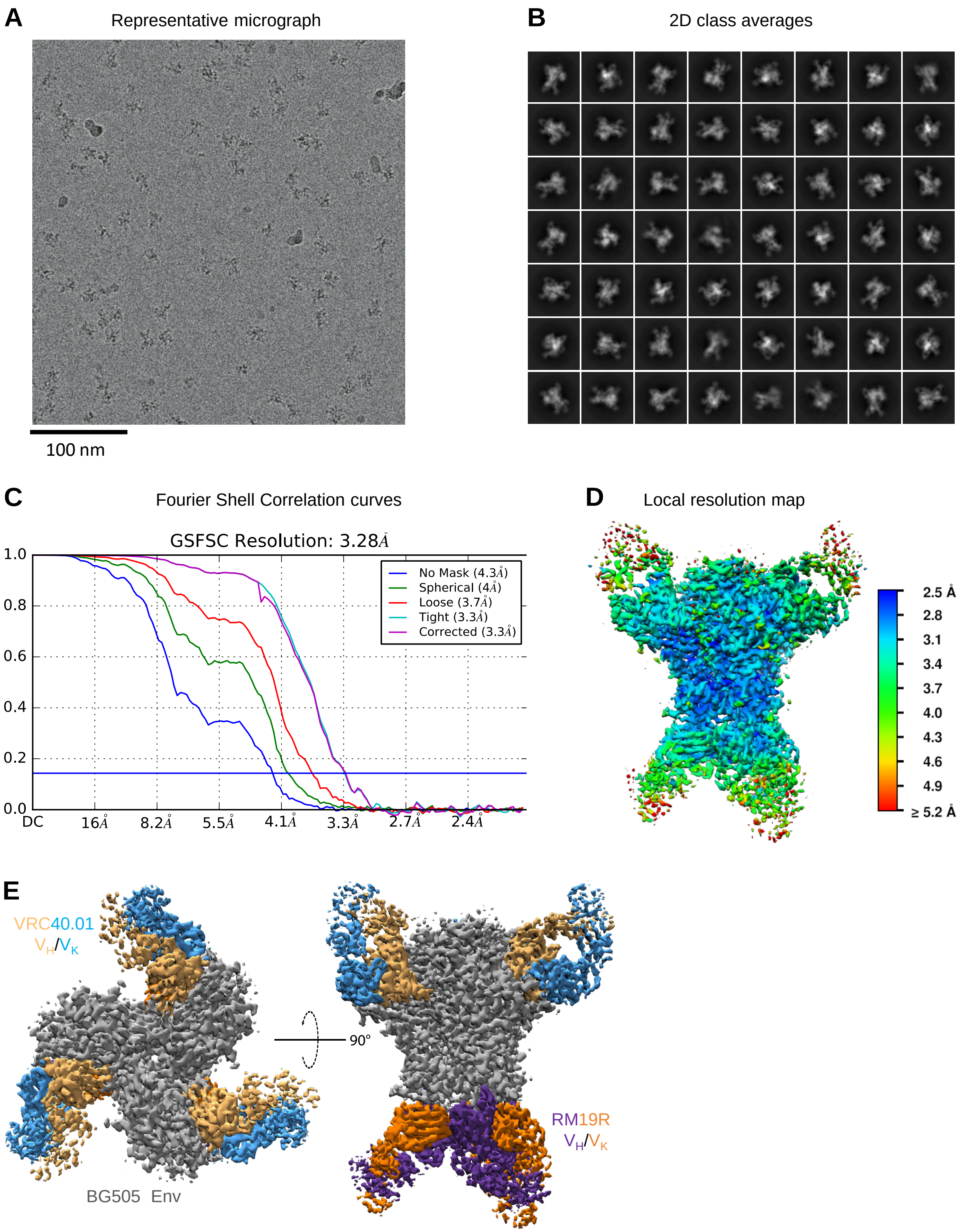

Figure S3. Cryo-EM data and structure validation for VRC40.01 and RM19R Fabs in complex with BG505 SOSIP.v5.2, related to Figure 2.
(**A**) Representative aligned and dose weighted micrograph collected on a Gatan K2 Summit. (**B**) Representative 2D class averages calculated from selected particles. (**C**) Gold-standard FSC curves of the final 3D reconstruction. (**D**) Refined 3D density map colored by local resolution. (**E**) 3.3-Å resolution cryo-EM reconstruction of VRC40.01 bound to BG505 SOSIP.v5.2. A base-binding monoclonal Fab RM19R was added to the complex to improve particle orientations.

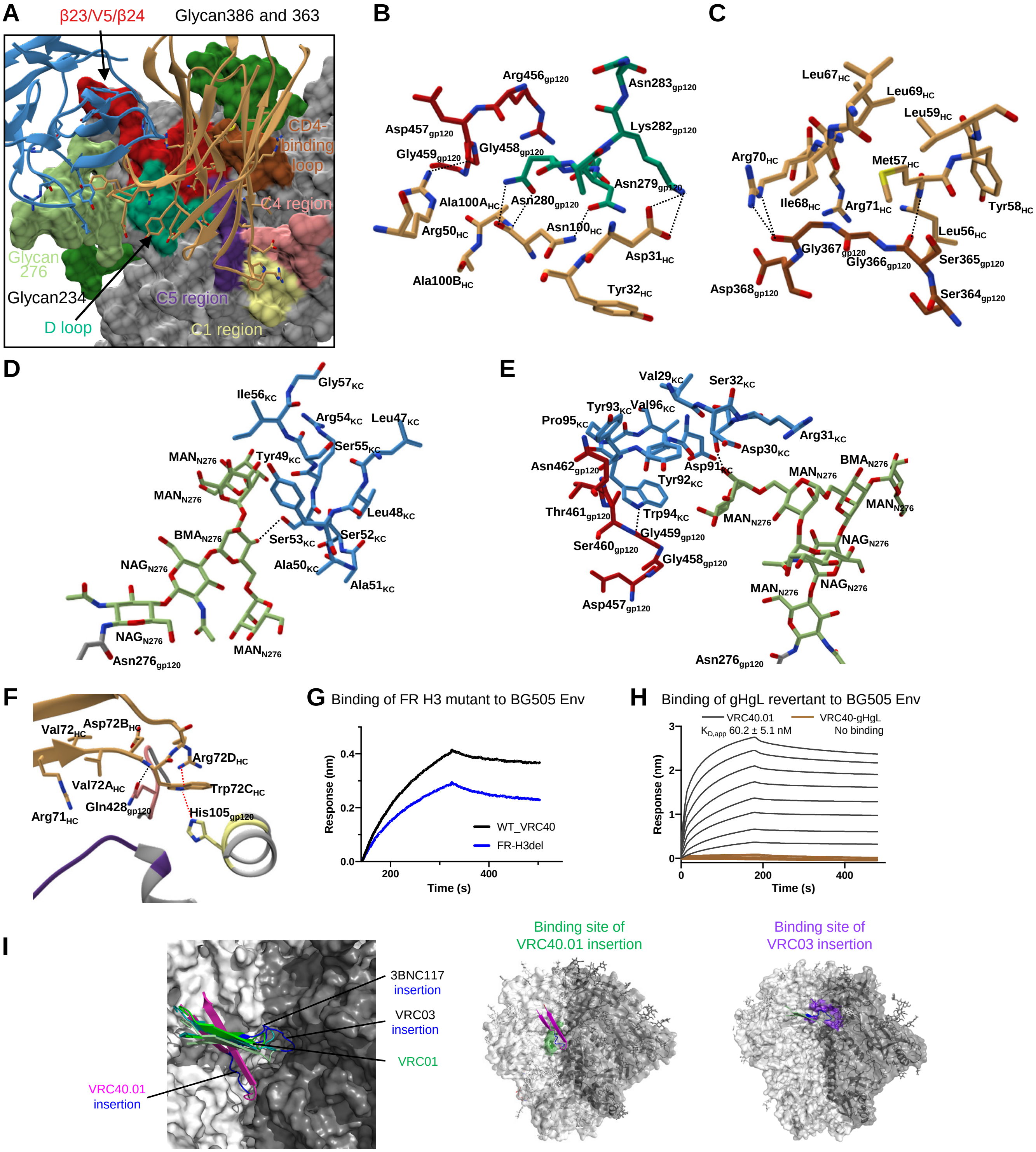

Figure S4. Structural and biochemical analyses of VRC40.01 binding to BG505 Env, related to Figure 2.
(**A**) Env gp120 regions at or near VRC40.01 epitope: glycan276 (light green), D loop (turquoise), *β*23/V5/*β*24 (red), CD4-binding loop (brown), C1 region (yellow), C4 region (pink), and C5 (purple). Glycans flanking the VRC40.01 epitope are shown in dark green. (**B**) Molecular interactions between VRC40.01 and the D loop and *β*23/V5/*β*24 regions. (**C**) Molecular interactions between VRC40.01 and the CD4-binding loop. (**D-E**) Interactions of the light chain of VRC40.01 with glycan276 and the *β*23/V5/*β*24 region of BG505. (**F**) VRC40.01 FR-H3 insertion makes contact with the C1, C4, C5 regions of BG505. Hydrogen bonds are shown as black dotted lines and π-π interactions are depicted as red dotted lines in (B) through (F). (**G**) BLI assays of BG505 SOSIP.664 binding to either the wildtype VRC40.01 Fab or a mutant of VRC40.01 Fab with the 5-residue FR-H3 insertion removed. (**H**) BLI assays of BG505 Env binding to either the wildtype or gHgL-reverted VRC40.01. BG505 Env trimer was captured on Streptavidin sensor tips and dipped into the antibody solutions at 0.05-6.5 μM. (**I**) Comparison of binding mode of FR-H3 insertions in VRC40.01 and VRC01-class antibodies. Contact surface of FR-H3 insertions are colored green and purple blue for VRC40.01 and VRC03, respectively.

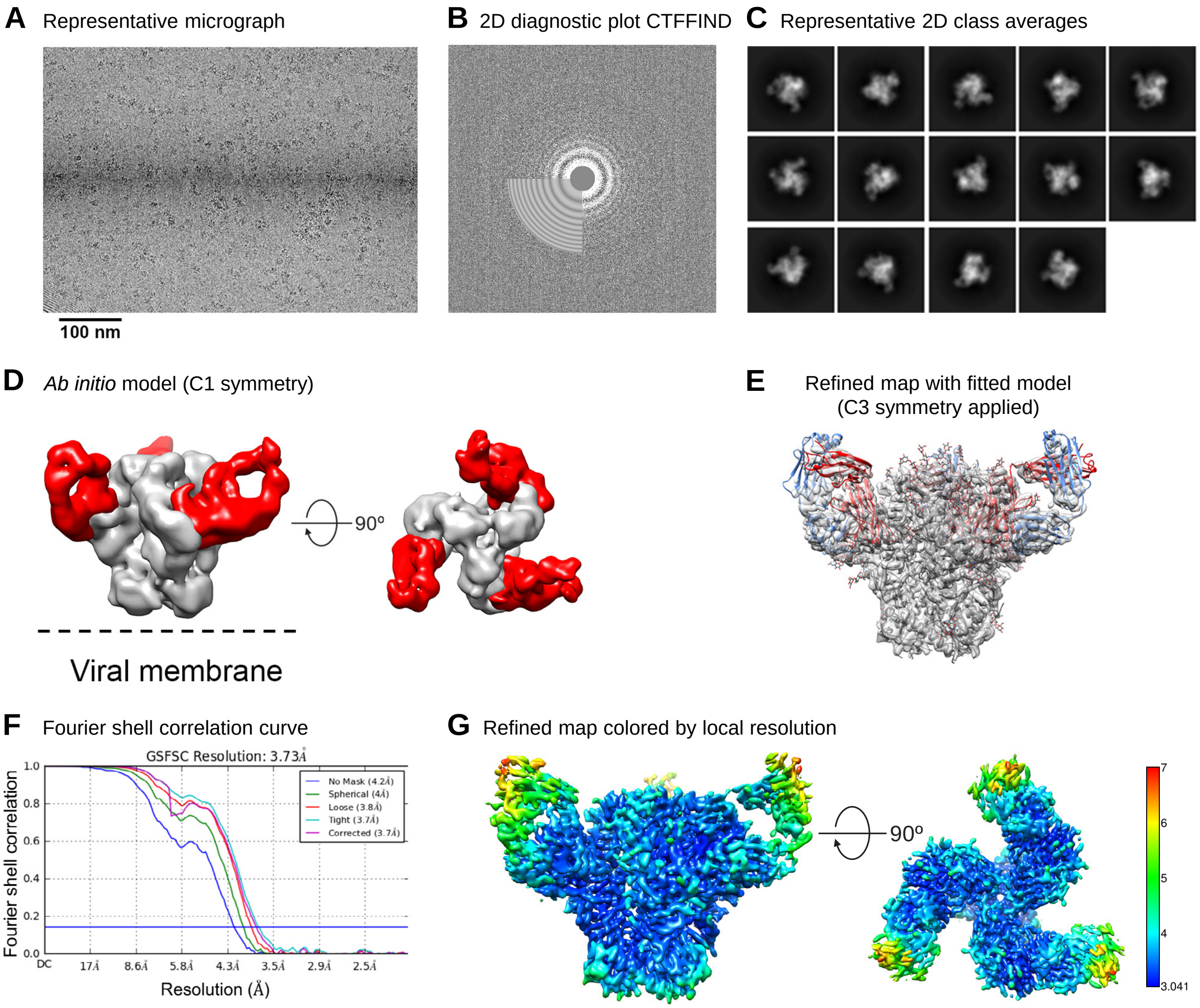

Figure S5. Cryo-EM data and structure validation for VRC40.01 Fab in complex with BG505 DS-SOSIP Env trimer, related to Figure 2.

(**A**) Representative micrograph of VRC40.01 Fab in complex with BG505 DS-SOSIP Env trimer. (**B**) 2D diagnostic plot of the micrographs using CTFFIND4. (**C**) Representative 2D class averages calculated from selected particles. (**D**) *Ab initio* model showing BG505 Env (grey) and VRC40.01 Fab (red). (**E**) C3 symmetry applied refined map of *ab initio* generated model refined against cleaned-up particles. (**F**) FSC curves of the 3D reconstructions with horizontal blue line indicating $FSC_{0.143}$. (**G**) Refined 3D density map colored by local resolution with side and top views.

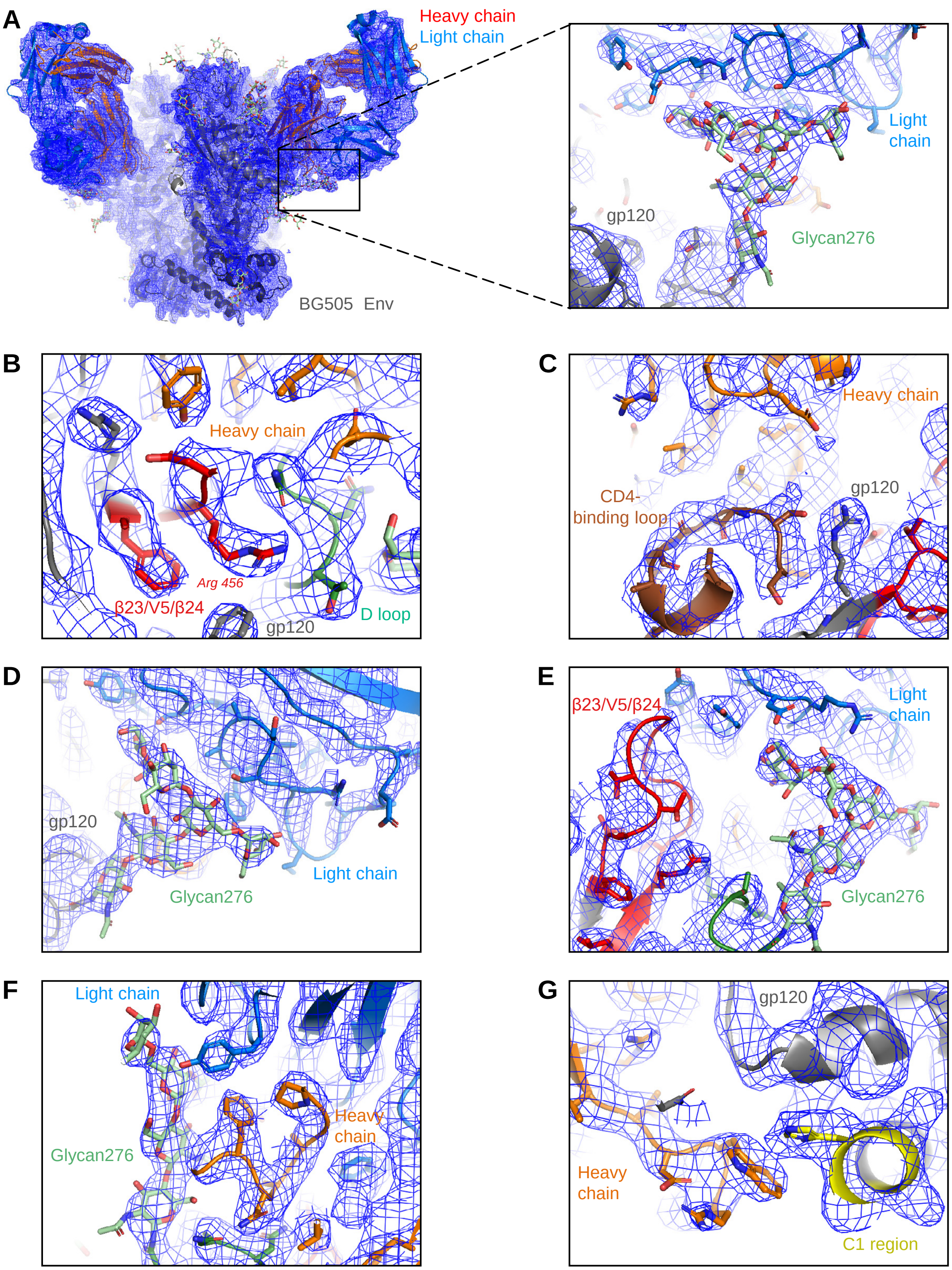

Figure S6. Cryo-EM density of VRC40.01 bound to BG505 DS-SOSIP Env trimer, related to Figure 2.
(**A**) Side view of VRC40.01 bound to BG505 DS-SOSIP structure fitted in cryo-EM density (blue mesh). Right panel shows a zoomed-in view of glycan276 fitted into the density. (**B-G**) Zoomed-in views of interactions between key regions of gp120 at or near the VRC40.01 epitope. Blue mesh indicates experimental cryo-EM density.

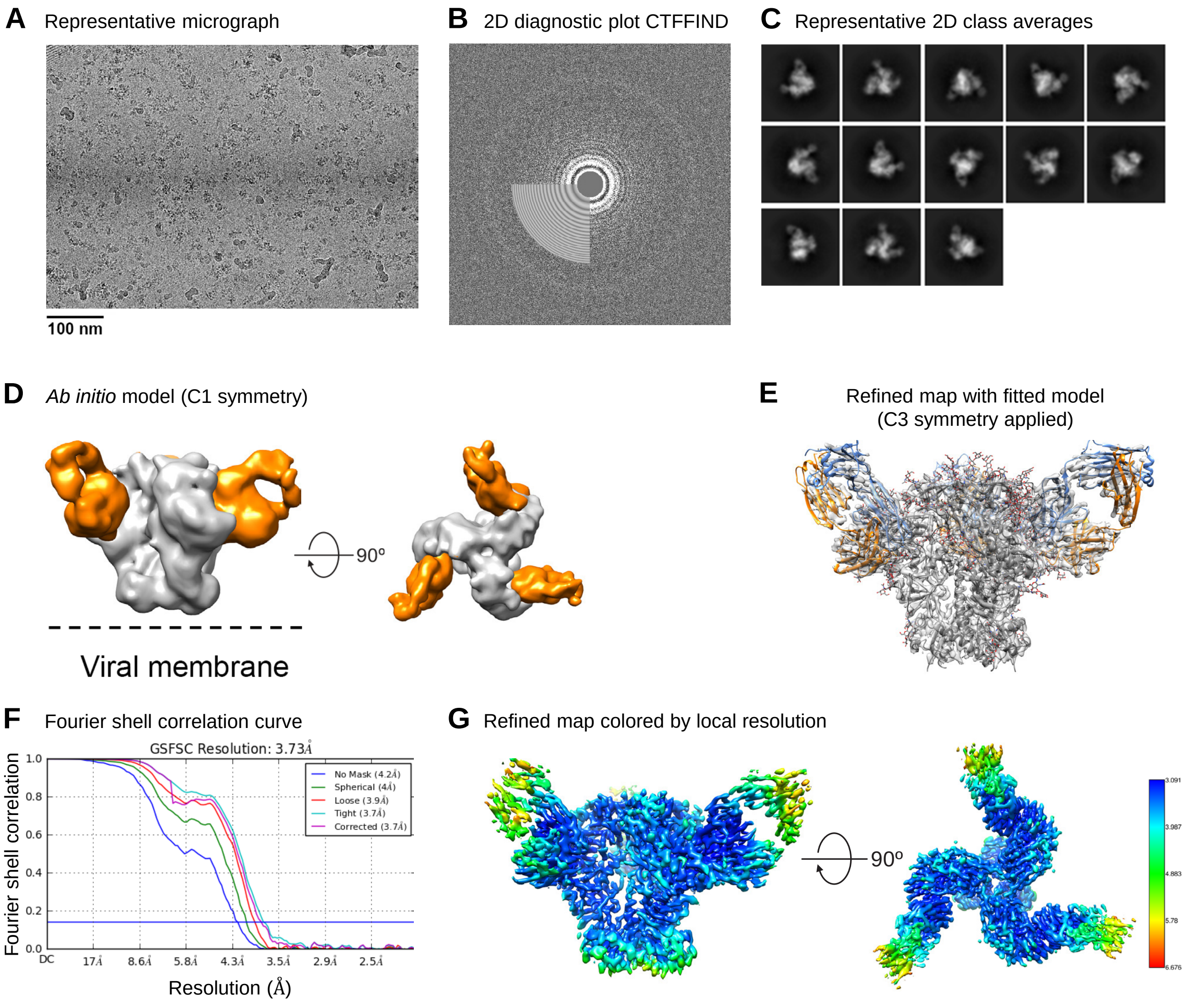

Figure S7. Cryo-EM data and structure validation for VRC33.01 Fab in complex with BG505 DS-SOSIP Env trimer, related to Figure 3.

(**A**) Representative micrograph of VRC33.01 Fab in complex with BG505 DS-SOSIP Env trimer. (**B**) 2D diagnostic plot of the micrographs using CTFFIND4. (**C**) Representative 2D class averages calculated from selected particles. (**D**) *Ab initio* model showing BG505 Env (grey) and VRC33.01 Fab (orange). (**E**) C3 symmetry applied refined map of *ab initio* generated model refined against cleaned-up particles. (**F**) FSC curves of the 3D reconstructions with horizontal blue line indicating $FSC_{0.143}$. (**G**) Refined 3D density map colored by local resolution with side and top views.

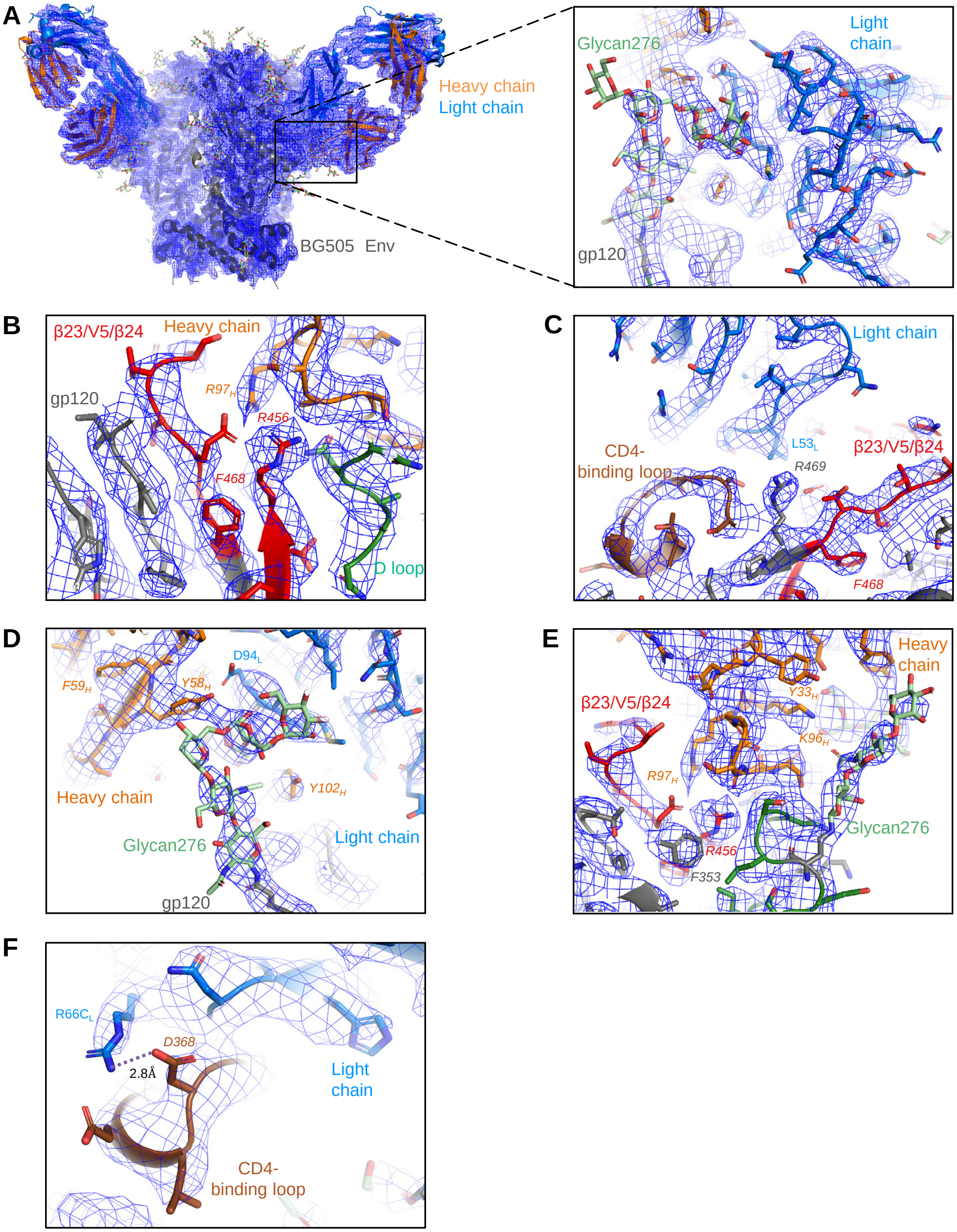

Figure S8. Cryo-EM density of VRC33.01 bound to BG505 DS-SOSIP Env trimer, related to Figure 3.
(**A**) Side view of VRC33.01 bound to BG505 DS-SOSIP structure fitted in cryo-EM density (blue mesh). Right panel shows a zoomed-in view of glycan276 fitting into the density. (**B-E**) Zoomed-in views of interactions between key regions of gp120 at or near the VRC33.01 epitope. Blue mesh indicates experimental cryo-EM density. (**F**) Asp368 of BG505 DS-SOSIP makes a salt bridge with Arg66C of VRC33.01 light chain.

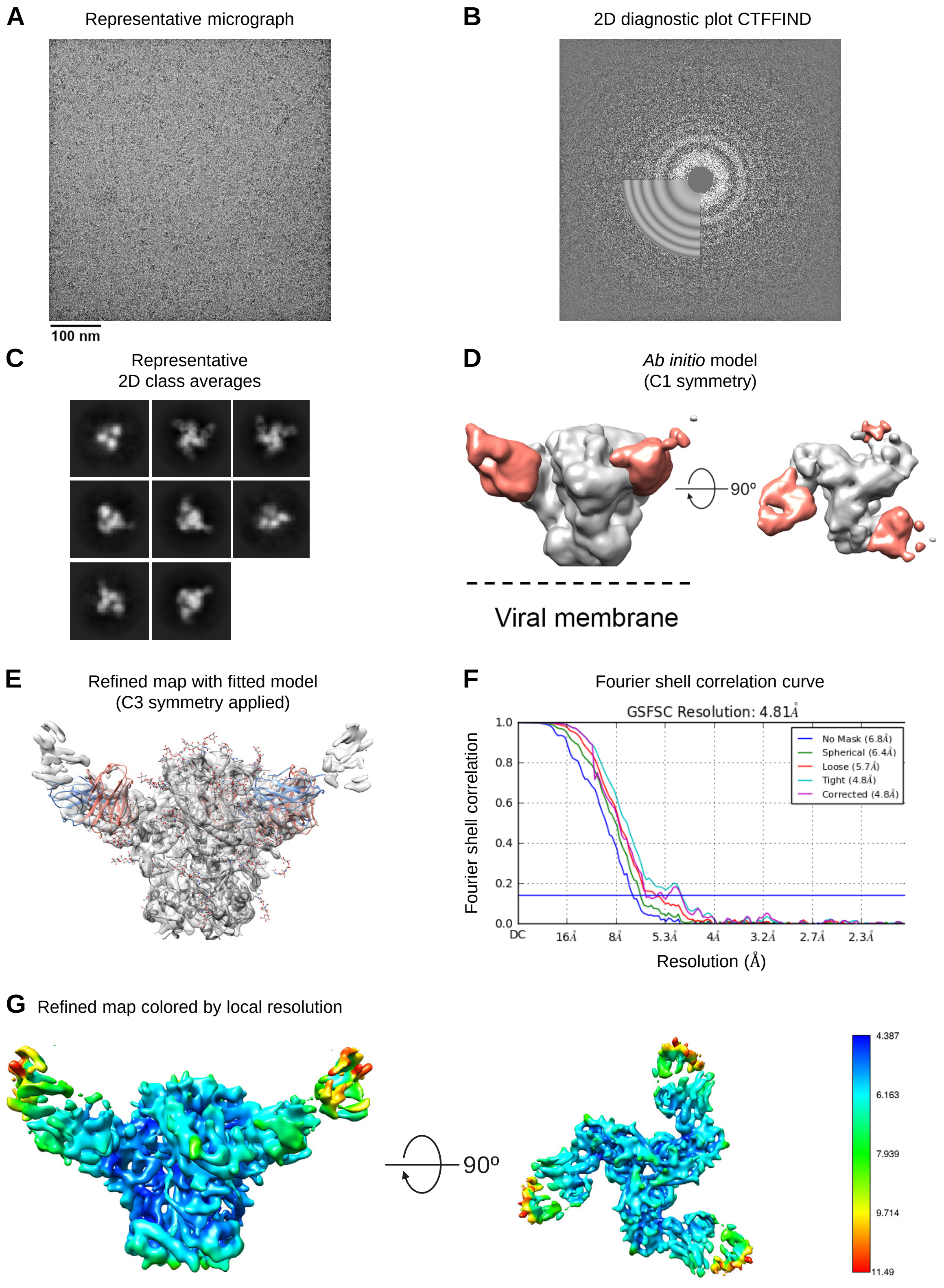

Figure S9. Cryo-EM data and structure validation for 179NC75 Fab in complex with Q23.17_DS-SOSIP_RnS_V1V2-V3-fixed Env trimer, related to Figure 5.

(**A**) Representative micrograph of 179NC75 Fab in complex with Q23.17_DS-SOSIP_RnS_V1V2-V3-fixed Env trimer. (**B**) 2D diagnostic plot of the micrographs using CTFFIND4. (**C**) Representative 2D class averages calculated from selected particles. (**D**) *Ab initio* model showing Q23.17_DS-SOSIP_RnS_V1V2-V3-fixed Env (grey) and 179NC751 Fab (salmon). (**E**) C3 symmetry applied refined map of *ab initio* generated model refined against cleaned-up particles. (**F**) FSC curves of the 3D reconstructions with horizontal blue line indicating $FSC_{0.143}$. (**G**) Refined 3D density map colored by local resolution with side and top views.

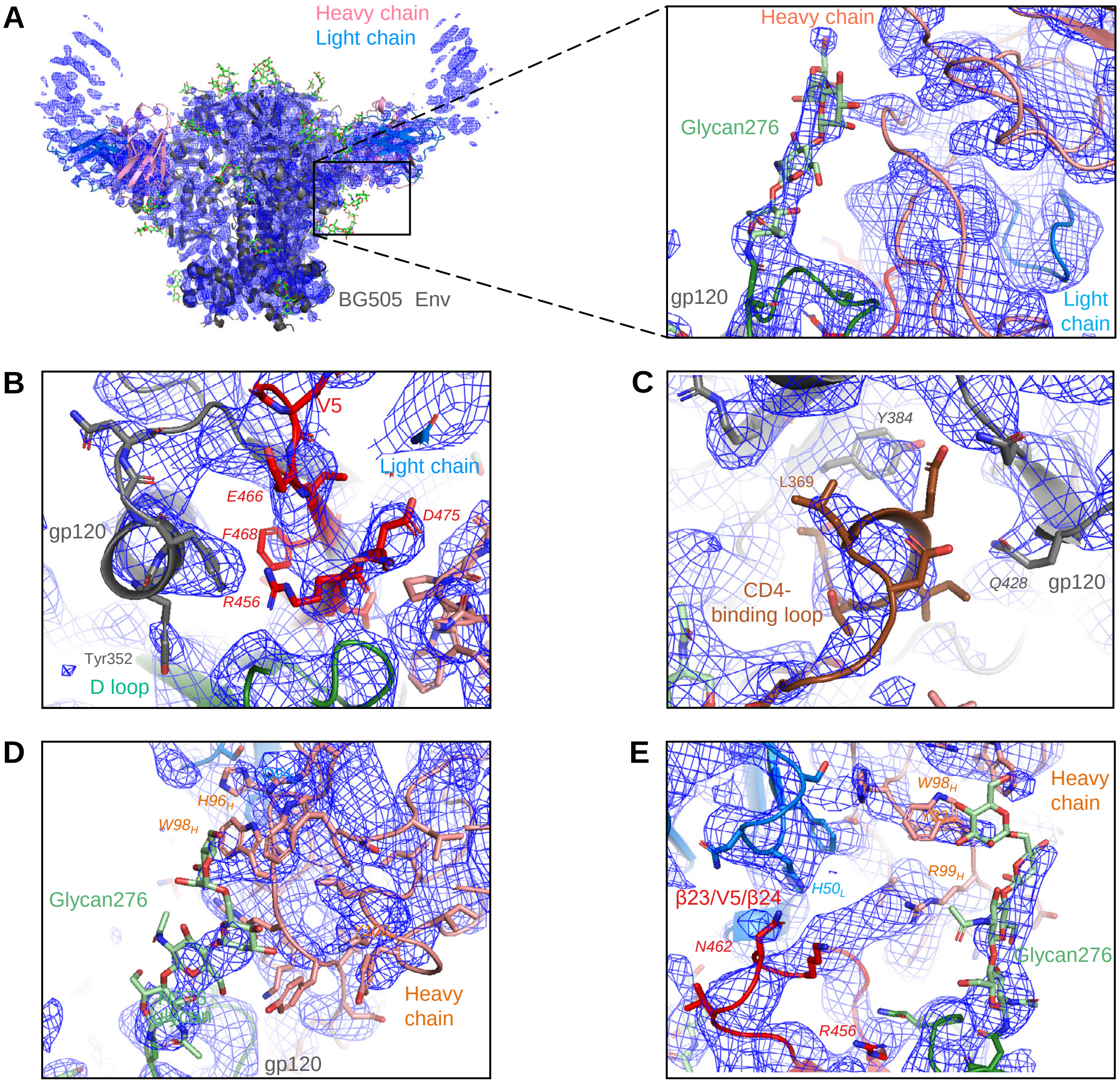

Figure S10. Cryo-EM density of 179NC75 Fab in complex with Q23.17_DS-SOSIP_RnS_V1V2-V3-fixed Env trimer, related to Figure 5.

(**A**) Side view of 179NC75 bound to Q23.17_DS-SOSIP_RnS_V1V2-V3-fixed structure fitted in cryo-EM density (blue mesh). Right panel shows a zoomed-in view of glycan276 fit into the density. (**B-E**) Zoomed-in views of interactions between key regions of Env gp120 regions at or near 179NC75 epitope. Blue mesh indicates experimental cryo-EM density.

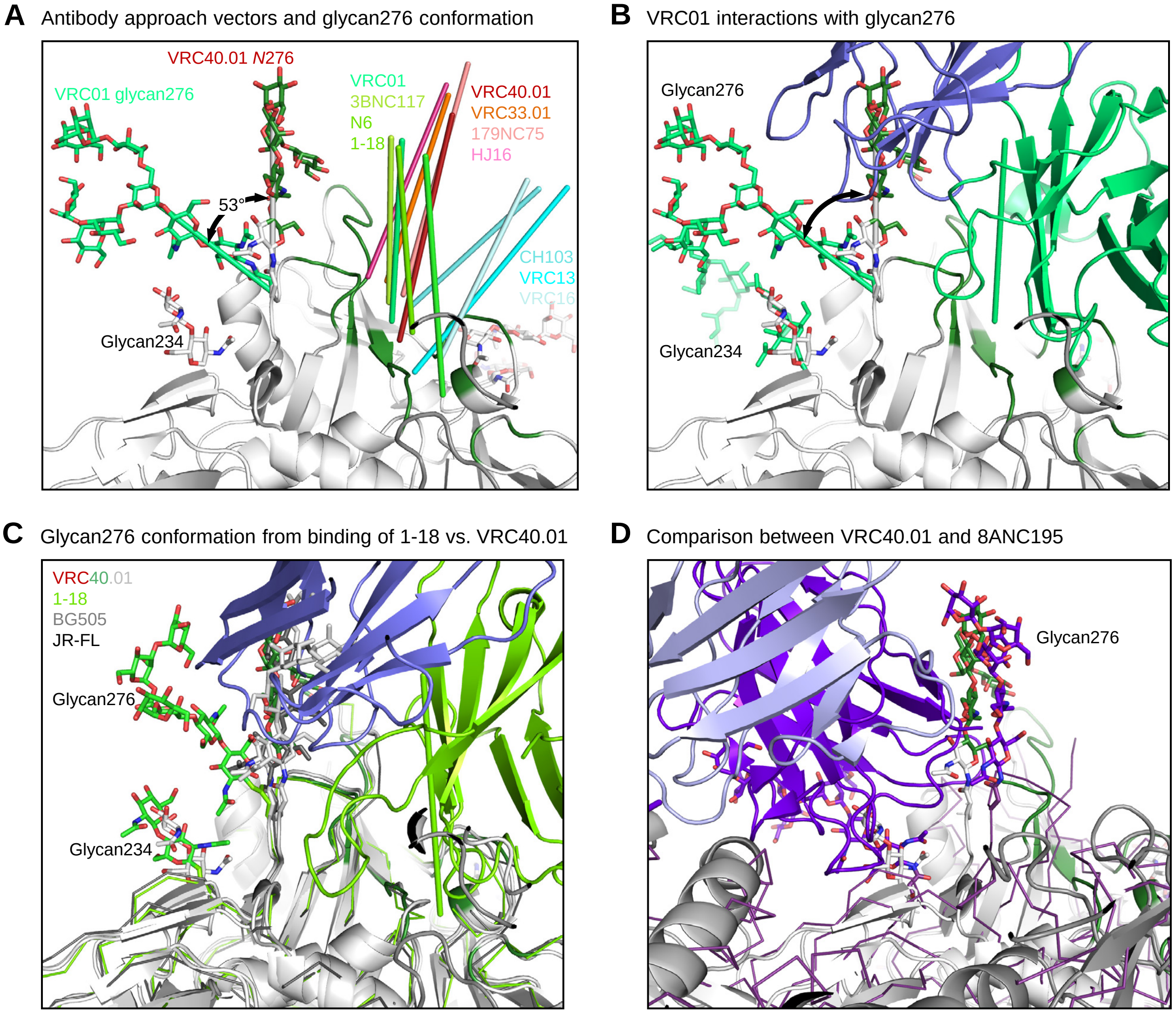

Figure S11. Antibody approach angles and glycan276 conformations, related to Figure 6.
(**A**) The angles and positions that antibodies approach Env trimer affect their interaction with glycan276. VRC01 binding tilts toward glycan276 relative to VRC40.01, and VRC01 binding pushes glycan276 53º away from CD4bs. BG505 Env is shown in light gray with VRC40.01 epitope residues in forest green. Glycan276 of the VRC01-Env complex is shown in lime green. Antibody approach vectors are shown as in Figure 6B. (**B**) VRC01 light chain interacts strongly with glycan276, which tilts away from the CD4bs to avoid clashes. (**C**) Effect on glycan276 conformation by binding of 1-18 in comparison with binding of VRC40.01 and glycan276 conformation free of glycan276-interacting antibodies. 1-18 (PDB: 6UDJ) is shown in yellow green with light chain in light blue. Only glycans at N276 and N234 are shown for other structures for clarity. Env structure without glycan276-binding antibodies (PDB: 5FYL, 5FUU) are colored as labeled. Env trimer in the VRC40.01 complex is shown as in (A). Binding of 1-18 pushes glycan276 away by 35º from that in VRC40.01, which maintains the orientation of glycan276 free of interacting antibody. (**D**) Glycan276 maintains the same conformation in 8ANC195 as in VRC40.01, despite their different binding locations on Env and binding glycan276 on opposite sides. 1-18 has strong interactions with glycan234.
